# Supplementary material for: Prediagnostic loss to follow-up in an active case finding tuberculosis programme: a mixed-methods study from rural Bihar, India
Source: BMJ Open. 2020 May 15;10(5):e033706. doi: 10.1136/bmjopen-2019-033706 (PMC7232626; doi:10.1136/bmjopen-2019-033706)
Supplement: Supplementary data [file bmjopen-2019-033706supp001.pdf]

SUPPLEMENTARY TABLE 1

Sensitivity analysis on including patient with negative diagnostic delay as zero day delay, after excluding patients with diagnostic evaluation before screening visit, and classifying patients with over 30 days delay in diagnostic evaluation as non-PDLFU

	**Including patients with negative diagnostic delay as 0 days diagnostic delay[$]**						**Excluding patients with negative diagnostic delay[$]**						**Including patients with diagnostic delay of over 30 days as non-PDLFU**					
Characteristics	Univariate analysis			Multivariate analysis			Univariate analysis			Multivariate analysis			Univariate analysis			Multivariate analysis		
	RR	95%CI	p-Value	aRR[^]	95%CI	p-Value	RR	95%CI	p-Value	aRR[^]	95%CI	p-Value	RR	95%CI	p-Value	aRR^	95%CI	p-Value
**Block**																		
Ujiarpur	1.0	(1.0-1.1)	0.022	-			1.1	(1.0-1.1)	0.004	-			1.0	(1.0-1.1)	0.04	-		
Bibhutipur	1.0	(1.0-1.1)	0.033	-			1.0	(1.0-1.1)	0.033	-			1.0	(1.0-1.1)	0.08	-		
Sarairanjan		Ref		-				Ref		-				Ref		-		
**Age**																		
<15	1.4	(1.3-1.4)	<0.001	1.2	(1.2-1.3)	<0.001	1.3	(1.3-1.4)	<0.001	1.3	(1.2-1.3)	<0.001	1.3	(1.2-1.4)	<0.001	1.2	(1.1-1.3)	<0.001
15-44	1.0	(1.0-1.1)	0.139	1.0	(1.0-1.1)	0.366	1.1	(1.0-1.1)	0.06	1.0	(1.0-1.1)	0.180	1.0	(1.0-1.1)	0.216	1.0	(1.0-1.1)	0.539
45-64	1.0	(1.0-1.1)	0.212	1.0	(1.0-1.1)	0.164	1.1	(1.0-1.1)	0.143	1.1	(1.0-1.1)	0.099	1.0	(1.0-1.1)	0.295	1.0	(1.0-1.1)	0.242
≥65		Ref			Ref			Ref			Ref			Ref			Ref	
**Gender**																		
Female		Ref			Ref			Ref			Ref			Ref			Ref	
Male	1.1	(1.0-1.1)	<0.001	1.1	(1.0-1.1)	0.001	1.1	(1.0-1.1)	<0.001	1.1	(1.0-1.1)	0.003	1.1	(1.0-1.1)	<0.001	1.1	(1.0-1.1)	<0.001
**Source of referral**																		
RMP[3]		Ref			Ref			Ref			Ref			Ref			Ref	
ASHA[1]	1.3	(1.2-1.4)	<0.001	1.2	(1.1-1.3)	<0.001	1.3	(1.2-1.4)	<0.001	1.2	(1.1-1.3)	<0.001	1.3	(1.2-1.4)	<0.001	1.2	(1.1-1.4)	<0.001
AWW[2]	1.3	(1.1-1.6)	0.003	1.2	(1.0-1.5)	0.025	1.3	(1.1-1.6)	0.005	1.2	(1.1-1.5)	0.030	1.4	(1.1-1.7)	<0.001	1.3	(1.1-1.6)	0.012
Community	1.4	(1.3-1.5)	<0.001	1.3	(1.2-1.4)	<0.001	1.4	(1.3-1.5)	<0.001	1.3	(1.2-1.4)	<0.001	1.4	(1.3-1.6)	<0.001	1.2	(1.2-1.5)	<0.001
**Signs and symptoms**																		
Haemoptysis in last 6 months	0.6	(0.6-0.7)	<0.001	0.7	(0.6-0.7)	<0.001	0.6	(0.6-0.7)	<0.001	0.8	(0.7-0.8)	<0.001	0.6	(0.6-0.7)	<0.001	0.7	(0.6-0.7)	<0.001
Cough ≥ 2 weeks	0.7	(0.6-0.7)	<0.001	-			0.7	(0.7-0.7)	<0.001	-			0.7	(0.6-0.7)	<0.001	-		
Sputum	0.6	(0.6-0.7)	<0.001	-			**0.7**	**(0.6-0.7)**	<0.001	-			0.6	(0.6-0.7)	<0.001	-		
Chest pain in last 1 month	0.6	(0.6-0.7)	<0.001	-			**0.7**	**(0.6-0.7)**	<0.001	-			0.6	(0.6-0.7)	<0.001	-		
Fever ≥ 2 weeks	0.8	(0.8-0.8)	<0.001	-			0.8	(0.8-0.8)	<0.001	-			0.8	(0.7-0.8)	<0.001	-		
Night sweats ≥ 2 weeks	0.8	(0.7-0.8)	<0.001	-			0.8	(0.7-0.8)	<0.001	-			0.8	(0.7-0.8)	<0.001	-		
Severe weight loss in last 3 months	0.7	(0.7-0.8)	<0.001	-			0.7	(0.7-0.8)	<0.001	-			0.7	(0.7-0.8)	<0.001	-		
Swelling in a lymph node	1.4	(1.4-1.5)	<0.001	-			1.4	(1.3-1.4)	<0.001	-			1.4	(1.3-1.4)	<0.001	-		

Garg T, *et al. BMJ Open* 2020; 10:e033706. doi: 10.1136/bmjopen-2019-033706

**Other factors**																		
Previous history of anti-TB treatment	0.7	(0.6-0.7)	<0.001	0.7	(0.7-0.8)	<0.001	0.7	(0.7-0.7)	<0.001	0.8	(0.7-0.8)	<0.001	0.7	(0.6-0.7)	<0.001	0.7	(0.7-0.8)	<0.001
Alcohol user	1.0	(0.9-1.2)	0.862	-			1.0	(0.9-1.1)	0.980	-			1.0	(0.9-1.1)	0.882	-		
Tobacco user	0.8	(0.8-0.9)	<0.001	-			0.9	(0.8-0.9)	<0.001	-			0.8	(0.8-0.9)	<0.001	-		

Bold variables indicate difference in RR

[$] Diagnostic delay is calculated as difference between date of first diagnostic evaluation and date of screening (Figure 1)

^aRR: Adjusted Risk Ratio

[1]ASHA: Accredited Social Health Activist

[2]AWW: Anganwadi Worker

[3]RMP: Registered Medical Practitioner, an informal provider

p-value ≤ 0.05 considered significant

* While building the model for multivariate Poisson’s regression analysis, all the signs and symptoms except hemoptysis were dropped because of high collinearity assessed on the basis of high VIF (Variance Inflation Factor). In addition, block and other factors like alcohol and tobacco use depicted no significant improvement in the model on the basis of likelihood ratio testing, hence, were also dropped.

Garg T, *et al. BMJ Open* 2020; 10:e033706. doi: 10.1136/bmjopen-2019-033706
