# Supplementary material for: Prediagnostic loss to follow-up in an active case finding tuberculosis programme: a mixed-methods study from rural Bihar, India
Source: BMJ Open. 2020 May 15;10(5):e033706. doi: 10.1136/bmjopen-2019-033706 (PMC7232626; doi:10.1136/bmjopen-2019-033706)
Supplement: Author's manuscript [file bmjopen-2019-033706.draft_revisions.pdf]

BMJ Open is committed to open peer review. As part of this commitment we make the peer review history of every article we publish publicly available.

When an article is published we post the peer reviewers' comments and the authors' responses online. We also post the versions of the paper that were used during peer review. These are the versions that the peer review comments apply to.

The versions of the paper that follow are the versions that were submitted during the peer review process. They are not the versions of record or the final published versions. They should not be cited or distributed as the published version of this manuscript.

BMJ Open is an open access journal and the full, final, typeset and author-corrected version of record of the manuscript is available on our site with no access controls, subscription charges or pay-per-view fees (http://bmjopen.bmj.com).

If you have any questions on BMJ Open's open peer review process please email info.bmjopen@bmj.com

BMJ Open

Pre-diagnostic loss to follow-up in an active case-finding TB program: a mixed-methods study from rural Bihar, India

Journal:	*BMJ Open*
Manuscript ID	bmjopen-2019-033706
Article Type:	Research
Date Submitted by the Author:	20-Aug-2019
Complete List of Authors:	Garg, Tushar; Innovators In Health, Research<br>Gupta, Vivek; All India Institute of Medical Sciences, Dr. R.P Centre for Ophthalmic Sciences<br>Sen, Dyuti; Innovators In Health, Operations<br>Verma, Madhur ; Kalpana Chawla Government Medical College, Community Medicine<br>Brouwer, Miranda; PHTB Consult, Consulting<br>Mishra, Rajeshwar; Innovators In Health, Research; Centre for Development of Human Initiatives<br>Bhardwaj, Manish; Innovators In Health, Operations
Keywords:	pre-diagnostic loss to follow-up, cascade of care, ASHA, Accredited Social Health Activist, Community health worker, screening

SCHOLARONE™
Manuscripts

I, the Submitting Author has the right to grant and does grant on behalf of all authors of the Work (as defined in the below author licence), an exclusive licence and/or a non-exclusive licence for contributions from authors who are: i) UK Crown employees; ii) where BMJ has agreed a CC-BY licence shall apply, and/or iii) in accordance with the terms applicable for US Federal Government officers or employees acting as part of their official duties; on a worldwide, perpetual, irrevocable, royalty-free basis to BMJ Publishing Group Ltd ("BMJ") its licensees and where the relevant Journal is co-owned by BMJ to the co-owners of the Journal, to publish the Work in this journal and any other BMJ products and to exploit all rights, as set out in our licence.

The Submitting Author accepts and understands that any supply made under these terms is made by BMJ to the Submitting Author unless you are acting as an employee on behalf of your employer or a postgraduate student of an affiliated institution which is paying any applicable article publishing charge ("APC") for Open Access articles. Where the Submitting Author wishes to make the Work available on an Open Access basis (and intends to pay the relevant APC), the terms of reuse of such Open Access shall be governed by a Creative Commons licence – details of these licences and which Creative Commons licence will apply to this Work are set out in our licence referred to above.

Other than as permitted in any relevant BMJ Author's Self Archiving Policies, I confirm this Work has not been accepted for publication elsewhere, is not being considered for publication elsewhere and does not duplicate material already published. I confirm all authors consent to publication of this Work and authorise the granting of this licence.

For peer review only

TITLE

Pre-diagnostic loss to follow-up in an active case-finding TB program: a mixed-methods study from rural Bihar, India

MANUSCRIPT TYPE

Research article

For peer review only

AUTHORS

Tushar Garg[1], Vivek Gupta[2], Dyuti Sen[1], Madhur Verma[3], Miranda Brouwer[4], Rajeshwar Mishra[1,5], Manish Bhardwaj[1]

1. Innovators In Health, Patna, India
2. Dr R P Centre for Ophthalmic Sciences, All India Institute of Medical Sciences, New Delhi, India
3. Kalpana Chawla Government Medical College, Karnal, India
4. PHTB Consult, Tilburg, The Netherlands
5. Centre for Development of Human Initiatives, New Jalpaiguri, India

ADDRESS FOR CORRESPONDENCE

Tushar Garg,
Innovators In Health, #176, Ground Floor, Road #2,
New Pataliputra Colony, Patna – 800013. Bihar. India.
Email: tgarg@innovatorsinhealth.org
Phone: +91 9828609562

MANUSCRIPT DETAILS

Abstract: 274 words

Manuscript: 4563 words

Tables: 2

For peer review only - http://bmjopen.bmj.com/site/about/guidelines.xhtml

Figures: 3

KEYWORDS

Pre-diagnostic loss to follow-up; cascade of care; screening; ASHA; Accredited Social Health Activist; community health worker

For peer review only

For peer review only - http://bmjopen.bmj.com/site/about/guidelines.xhtml

ABSTRACT

objective

Our objective was to quantify the pre-diagnostic loss to follow-up (PDLFU), and identify the barriers and enablers in undergoing a diagnostic evaluation in an active case-finding TB program.

design

Explanatory mixed-methods design.

setting

A rural population of 1.02 million in Samastipur district of Bihar, India.

participant

During their routine work, Accredited Social Health Activists (ASHA, a community health worker or CHW), informal providers, and community laypersons identified people at risk of TB, and referred them to the program. We included 13395 such community referrals received between January 2018 to December 2018.

Program's field coordinator (FC) screened them for TB symptoms. Identified presumptive TB cases were accompanied by the CHW for diagnostics and supported throughout treatment. We conducted in-depth interviews with patients who were evaluated (n = 3) and those who were not evaluated (n = 4), and focus group discussions with the CHWs (n = 2) and FC (n = 1).

outcome measures

Proportion and characteristics of PDLFU, and association of demographic and symptom characteristics with diagnostic evaluation

results

A total of 11146 presumptive TB cases were identified from January 2018 to December 2018, out of which 4912 (44.1%) underwent a diagnostic evaluation. The key enablers were CHW accompaniment and support in addition to the free TB services in the public sector. The major

For peer review only - http://bmjopen.bmj.com/site/about/guidelines.xhtml

barriers identified were transport challenges, deficient family and health provider support, and poor services in the public system.

conclusion

If we are to find missing cases, the health system needs urgent reform, and diagnostic services need to be patient-centric. A strong patient support system engaging all stakeholders and involvement of CHWs in routine TB care is an effective solution.

STRENGTHS AND LIMITATIONS OF THIS STUDY

1. First such study to explore the reasons for pre-diagnostic loss to follow-up
2. A mixed-method design including the views of both patients and community health workers
3. Uses operational data from a routine programmatic setting at an NGO site
4. No record of the actual number of people screened intuitively before being referred to the program.
5. No record of patients accessing diagnostics in private sector and those completing the diagnostic process.

For peer review only

For peer review only - http://bmjopen.bmj.com/site/about/guidelines.xhtml

INTRODUCTION

The End TB strategy targets an 80% reduction in TB incidence by 2030.[1] However, as many as 4.1 million (39%) patients globally are not notified, indicating a mix of under-reporting and under-diagnosis.[2] Of these, India alone has about a million.[3,4] Analysis of the cascade of care can effectively quantify the gaps in successive steps of TB care pathway and informs the TB programs of potential interventions.[4] The Indian public sector cascade and the South African cascade estimate a loss to follow up (LFU) at test access of 28% and 5% respectively out of incident cases.[5,6] Similar approaches can be used to evaluate case-finding programs involving systematic TB screening.[5]

Historically, most TB patients were identified through passive case-finding in which patients self-referred to a provider for a diagnostic evaluation.[7] It may lead to delays in diagnosis and continued transmission in the community.[8] The known major challenges in diagnosis are poor geographical and financial access to healthcare, fewer symptoms delay seeking healthcare, and failure to test when people do present at the facility.[9]

Diverse active case finding (ACF) strategies have demonstrated increased TB case detection with early diagnosis.[7,10,11] Nonetheless, multiple ACF interventions have reported high PDLFU. A study from India reported only 22% of the people with presumptive TB reaching a microscopy center, while an ACF program in Myanmar reported only 51.4% of patients with abnormal chest X-ray (CXR) getting their sputum examined.[12,13] A recent study of a TB program in a district of Zimbabwe reported a PDLFU of 25% with patient's residence, HIV status and type of health facility emerging as significant risk factors.[14] Although these studies rely on routinely collected programmatic data, there has been no study exploring the reasons for the high PDLFU and the perspectives of associated stakeholders.

We aimed to identify the PDLFU, and assess the enablers and barriers for diagnostic evaluation in a community-based ACF program during 2018 in Bihar, India. The specific objectives were: 1) to assess proportion of people screened among community referrals, 2) to assess number of individuals with presumptive TB and their characteristics, 3) to assess proportion and

For peer review only - http://bmjopen.bmj.com/site/about/guidelines.xhtml

characteristics of PDLFU patients, and identify risk factors, and 4) to understand the reasons for the PDLFU, including barriers and enablers to access the diagnostic evaluation.

METHODS

study design

We used an explanatory mixed-methods study design, where the quantitative phase (cohort analysis) was followed by a qualitative phase (descriptive design).[15]

study setting

General setting

The study was conducted in the Sarairanjan, Bibhutipur, and Ujiarpur blocks (equivalent to TB Unit or TU) of Samastipur district in Bihar with a combined population of 1,021,483. Located in the Indo-Gangetic plain, agriculture was the main occupation of the population. The highest earning member in 69.8% of the households earned less than INR 60,000 annually (~USD 852) and 63.5% of the population was reported literate.[16] Females had 20% lesser literacy rate compared to males, and infant mortality rate (IMR) was 60 for females against that of 48 for males.[17] The public TB case notification rate in the district was 55 per 100,000 population (2017) and the pre-treatment LFU was 25% (2017).[18]

The population was serviced by 12 primary health centres (PHC) under the Public Health System (PHS).[19] Within the Revised National TB Control Program (RNTCP), 4 designated microscopy centres (DMC) run by laboratory technicians provided sputum microscopy. A Senior Treatment Supervisor (STS) at the TU level managed the program and a Senior Treatment Laboratory Supervisor (STLS) oversaw a group of DMCs.

The last-mile TB care services were provided by a Community Health Worker (called ASHA or Accredited Social Health Activist). Initiated under the National Health Mission (NHM) in 2005, an ASHA is a literate woman of the village and assigned for every 1000 population. Her key responsibility is to be a facilitator, mobilizer, and community service provider for various public

For peer review only - http://bmjopen.bmj.com/site/about/guidelines.xhtml

health programs.[20] Under the RNTCP, ASHA received activity-based remuneration: INR 1000 (~ USD 14) for DS-TB treatment support, and INR 5000 (~ USD 72) for DR-TB treatment support.[21]

Anganwadi workers (AWW) are community health workers under the Integrated Child Development Services Scheme and receive a fixed monthly honorarium. Through Angadwadi Centres, they provide early childhood care and education for children up to 6 years.[22]

Specific setting

We implemented a TB REACH Wave 5-funded novel, community-based ACF project in collaboration with the RNTCP in the above population. The key aspects were: 1) community referral of people at risk of TB, 2) symptom-based screening at patient's doorstep, 3) ASHA-accompanied diagnostic evaluation, 4) training to ASHA, 5) extensive stakeholder engagement, and 6) engaging multiple community members, including AWW and informal providers (also known as RMP or Registered Medical Practitioner).

After an initial dialogue, ASHA, AWW, RMPs and the community were asked to refer people who may have TB. They relied on their existing knowledge of the community and looked for people at risk of TB during their routine work and social activities. These community referrals were reported to a field coordinator (FC) of the project, who screened them at their home for TB symptoms systematically. All presumptive TB cases were referred for a sputum microscopy and chest x-ray (CXR). All people with a positive smear, or people with a negative smear but abnormal CXR, or people with a negative smear but clinically suspect were offered a GeneXpert test using sputum collection and transport. ASHAs assisted presumptive TB patients in reaching the diagnostic centres and accompanied them through the process. Patients received a transport allowance and diagnostic tests were free of cost. If the PHS physician confirmed TB diagnosis, treatment was initiated under ASHA's supervision. The ASHA delivered drugs to the patient, and supported them till treatment completion.[Figure 1]

A conditional incentive of INR 200 (~USD 3) for community referral and INR 300 (~ USD 5) for each confirmed diagnosis of TB was given by the project. These incentives to the ASHA were in addition to the RNTCP treatment support incentives. The project's FCs received a fixed monthly salary.

For peer review only - http://bmjopen.bmj.com/site/about/guidelines.xhtml

The project used a Management Information System (MIS) developed in Microsoft Excel which maintained individual patient data electronically. On receipt of a community referral, the FC enlisted them in a referral register with a unique ID and a separate form was completed for screening. The status of each community referral was tracked using the referral register. A patient-wise folder with various forms and diagnostic reports was maintained for all presumptive TB cases undergoing diagnostic evaluation. Data were entered into the MIS weekly and at least 20% entries were verified monthly in a two-person formation to assess data entry quality.

study population and study period

Quantitative

All community referrals in the 3 selected blocks in Samastipur district between January 01, 2018 to December 31, 2018 were included in the study. There was no sampling involved.

Qualitative

Presumptive TB cases referred for diagnostic evaluation were interviewed. A total of 7 in-depth interviews were conducted with 3 presumptive TB cases who were evaluated (2 females, 1 male, age range: 17 – 30 years) and 4 presumptive cases who were not evaluated (2 females, 2 males, age range: 6 – 65 years). A purposive sample of ASHAs and field coordinators was selected for the focus group discussions (FGD). Three FGDs were conducted with a group of 8 ASHAs (all females, age range: 24 – 49), 11 ASHAs (all females, age range: 22 – 54), and 9 field coordinators (6 females, 3 males, age range: 20 – 38). The average duration was 36 minutes (range 23 – 51 minutes). A diverse sample of males and females across age groups was purposively selected for diversity and interviewed till saturation was achieved.

data variables, sources of data and data collection

Quantitative

Data on the community referrals' characteristics (location, referral source, age, gender), screening criteria (date of screening, symptoms, outcome), and diagnostic evaluation (date of

For peer review only - http://bmjopen.bmj.com/site/about/guidelines.xhtml

test, type of test) were extracted from the MIS into a structured proforma. The data extracted from the MIS was de-identified before being exported for analysis. [Figure 1]

Qualitative

Presumptive TB cases were interviewed by TG (a male medical doctor trained in qualitative research) and DS (a female economist trained in qualitative research), and FGDs were conducted by TG and RM (a male professor of social psychology). All the three investigators have long and adequate experience of the socio-cultural context of the region, and understanding of TB programs. After obtaining a participant's consent, the interviews were conducted at a time and place convenient for the participant and privacy was ensured. The regional vernacular was used for interactions, and audio-recorded using voice recorder after obtaining consent. The objectives of the study were explained to the participants. No participant denied permission for an interview and there were no repeat interviews. A topic guide was used for FGDs and an interview guide for in-depth interviews to explore the enabling factors and barriers for diagnostic evaluation. Appropriate probes were used for clarity and to elicit information. The information was debriefed for participant validation after the interview, but the transcripts were not returned to them.

analysis and statistics

Quantitative

The data were analysed using EpiData Analysis (version 2.2.2.183, EpiData Association, Odense, Denmark) and Stata (version 15.1, StataCorp LLC, College Station, Texas, USA). Patients not undergoing a diagnostic test for TB within 30 days of referral were considered PDLFU. Patients with diagnostic test after 30 days (n = 140) were excluded from the diagnostic evaluation category. In case patients had diagnostic test performed prior to community referral, the time to diagnostic visit was set as 0 days (n = 48). Age was missing for 242 community referrals.[Figure 1] As applicable, variables were summarized with mean (and standard deviation or SD) or median (and inter-quartile range or IQR) based on statistical distribution of data, or frequencies and percentages. Association of demographic and symptom characteristics with diagnostic evaluation was analyzed using χ2-square test and unadjusted relative risk (RR) with 95%

For peer review only

For peer review only - http://bmjopen.bmj.com/site/about/guidelines.xhtml

confidence interval was calculated. A multivariate analysis using Poisson regression was used to calculate adjusted relative risk (aRR) with 95% confidence interval. P-value ≤ 0.05 was considered statistically significant.

Qualitative

The transcripts were prepared on the same day using audio recording and field notes by TG. Manual descriptive content analysis was performed by two independent, trained researchers (TG, DS) to generate categories and themes.[23] Any discrepancies between the two were resolved through discussion. These were discussed and reviewed by RM to avoid subjective bias. The codes and themes were related back to the original data to ensure that the results reflect the data.[24]

For peer review only

patient and public involvement

Patients were neither involved in the study design nor in the interpretation of patient relevant outcomes. Nonetheless, patient's views were sought in the qualitative interviews and included in the results. The results of this study will be communicated to the patients and the public through a newsletter in the vernacular.

RESULTS

care cascade and characteristics of presumptive TB cases

We received a total of 13395 community referrals, out of which 90.9% (n=12180) were screened for symptoms. Of those screened, 91.5% (n=11146) were presumptive TB cases, and referred for a diagnostic evaluation. (Figure 2)

There was nearly equal representation of presumptive TB cases from all the 3 blocks, and ASHAs identified most of them (75.6%). The mean age of presumptive TB cases was 35 years with majority in the 15-44 age group (41.8%). There were more males (52.2%) than females. (Table 1)

Characteristics	Community referrals, n(%)	Screened for TB, n(%)	Presumptive TB cases, n(%)

For peer review only - http://bmjopen.bmj.com/site/about/guidelines.xhtml

**Total**	13395		12180		11146	
**Block**						
Ujiarpur	4275	(31.9%)	3926	(32.2%)	3620	(32.5%)
Bibhutipur	4609	(34.4%)	3994	(32.8%)	3673	(33%)
Sarairanjan	4511	(33.7%)	4260	(35.0%)	3853	(34.5%)
**Age**						
<15	2847	(21.3%)	2611	(21.4%)	2385	(21.4%)
15-44	5641	(42.1%)	5147	(42.3%)	4661	(41.8%)
45-64	3330	(24.9%)	3032	(24.9%)	2789	(25.0%)
≥65	1335	(10.0%)	1243	(10.2%)	1169	(10.5%)
Missing	242	(1.8%)	147	(1.2%)	142	(1.3%)
**Gender**						
Male	6973	(52.1%)	6391	(52.5%)	5827	(52.2%)
Female	6422	(47.9%)	5789	(47.5%)	5319	(47.7%)
**Source of referral**						
ASHA[1]	10091	(75.3%)	9210	(75.6%)	8428	(75.6%)
AWW[2]	105	(0.8%)	101	(0.8%)	88	(0.8%)
RMP[3]	724	(5.4%)	661	(5.4%)	617	(5.5%)
Community	2475	(18.5%)	2208	(18.1%)	2013	(18.1%)

[1]ASHA: Accredited Social Health Activist

[2]AWW: Anganwadi Worker

[3]RMP: Registered Medical Practitioner, an informal provider

The most common symptoms among presumptive TB cases were cough ≥ 2 weeks (79.8%%), severe weight loss in 3 months (74.6%), fever ≥ 2 weeks (73.9%), and chest pain in the last 1 month (63.4%), while hemoptysis in the last 6 months (12.1%) was the least common. Nearly one-fifth of the presumptive TB cases reported a previous history of anti-TB treatment (22.4%), and 20.5% were tobacco users. (Table 2)

Characteristics	Presumptive TB cases, n	Not evaluated, n(%)		Unadjusted RR (95% CI)		p-value	Adjusted RR (95% CI)		p-value
**Total**	11146	6234	(55.9%)						
**Block**									
Ujiarpur	3620	2060	(56.9%)	1.0	(1.0 - 1.0)	0.87	1.1	(1.0 - 1.1)	0.00
Bibhutipur	3673	2083	(56.7%)	Ref			Ref		
Sarairanjan	3853	2091	(54.3%)	1.0	(0.9 - 1.1)	0.03	1.0	(1.0 - 1.1)	0.14

For peer review only - http://bmjopen.bmj.com/site/about/guidelines.xhtml

**Age**									
<15	2385	1625	(68.1%)	1.3	(1.2 - 1.4)	0.00	1.0	(0.9 - 1.1)	0.37
15-44	4661	2459	(52.8%)	1.0	(1.0 - 1.1)	0.83	0.9	(0.9 - 1.0)	0.00
45-64	2789	1464	(52.5%)	Ref			Ref		
≥65	1169	588	(50.3%)	1.0	(0.9- 1.0)	0.21	1.0	(0.9 - 1.1)	0.65
**Gender**									
Male	5319	2859	(53.8%)	Ref			Ref		
Female	5827	3375	(57.9%)	0.9	(0.9 - 1.0)	0.00	1.0	(0.9 - 1.0)	0.00
**Source of referral**									
ASHA[1]	8428	4690	(55.7%)	0.9	(0.8 - 1.1)	0.50	1.0	(0.8 - 1.1)	0.70
AWW[2]	88	52	(59.1%)	Ref			Ref		
RMP[3]	617	271	(43.9%)	0.7	(0.6 - 0.9)	0.00	0.8	(0.7 - 1.0)	0.08
Community	2013	1221	(60.7%)	1.0	(0.9 - 1.2)	0.77	1.0	(0.9 - 1.2)	0.91
**Signs and symptoms**									
Cough ≥ 2 weeks	8895	4514	(50.8%)	0.7	(0.6 - 0.7)	0.00	0.9	(0.9 - 1.0)	0.00
Sputum	6184	2779	(44.9%)	0.6	(0.6 - 0.7)	0.00	0.8	(0.8 - 0.8)	0.00
Hemoptysis in last 6 months	1346	494	(36.7%)	0.6	(0.6 - 0.7)	0.00	0.8	(0.8 - 0.9)	0.00
Chest pain in last 1 month	7061	3260	(46.2%)	0.6	(0.6 - 0.7)	0.00	0.8	(0.8 - 0.8)	0.00
Fever ≥ 2 weeks	8242	4289	(52.0%)	0.8	(0.8 - 0.8)	0.00	0.9	(0.9 - 1.0)	0.00
Night sweats ≥ 2 weeks	3730	1724	(46.2%)	0.8	(0.7 - 0.8)	0.00	0.9	(0.8 - 0.9)	0.00
Severe weight loss in last 3 months	8318	4254	(51.1%)	0.7	(0.7 - 0.8)	0.00	0.9	(0.9 - 0.9)	0.00
Swelling in a lymph node	2067	1517	(73.4%)	1.4	(1.4 - 1.5)	0.00	1.0	(1.0 - 1.0)	0.96
**Other factors**									
Previous history of anti-TB treatment	2501	1034	(41.3%)	0.7	(0.7 - 0.7)	0.00	0.8	(0.8 - 0.9)	0.00
Alcohol user	159	90	(56.6%)	1.0	(0.9 - 1.2)	0.86	1.2	(1.1 - 1.4)	0.01
Tobacco user	2280	1109	(48.6%)	0.8	(0.8 - 0.9)	0.00	1.0	(0.9 - 1.1)	0.99

[1]ASHA: Accredited Social Health Activist

[2]AWW: Anganwadi Worker

[3]RMP: Registered Medical Practitioner, an informal provider

Age missing for 142 presumptive cases and 98 not evaluated

All % are row percentages.

p-value ≤ 0.05 considered significant

characteristics of pre-diagnostic LFU and associated risk factors

Nearly 44% (n=4912) of the presumptive TB cases received a diagnostic evaluation. The pre-diagnostic LFU was highest among younger (< 15 year) presumptive TB cases (68.1%), while lesser among those who were referred by an RMP (43.9%) and complained of hemoptysis in the last 6

For peer review only

For peer review only - http://bmjopen.bmj.com/site/about/guidelines.xhtml

months (36.7%). On multivariate analysis, presumptive TB cases who were between 15 to 44 years of age (aRR 0.9, 95% CI: 0.9 – 1.0, p = 0) were more likely to be LFU in the pre-diagnostic stage. All of the clinical features of presumptive TB were also associated with lower LFU except lymph node swelling. Alcohol use was an independent risk factor for LFU (aRR 1.2, 95% CI: 1.1 – 1.4, p = 0.01). The median time to diagnosis was 1 day (IQR = 3). (Table 2)

Enablers to access the first diagnostic evaluation

In the interviews, patients reported the following enablers in getting a diagnostic evaluation: transport allowance for travel to the hospital, free services in the PHS and knowledge of the PHS procedures, accompaniment of ASHA and her assistance in the diagnostic process, and ASHA's understanding of the PHS functioning. (Figure 3)

> *"I started facing financial problems after going to the private hospital… Had I known the quality of care in the government hospital earlier, I would have come here. It helped that I didn't have to spend any money. I went by a vehicle to the PHC and consulted the doctor for free of cost."* (23 years old male patient)

> *"I didn't have to go to the hospital after submitting the sample for diagnostics… ASHA took me to the hospital and now sends the medicine… I was bony and couldn't even move a step earlier… I became better only from these government drugs; rest were useless. Between RMP and private clinic, we spent nearly □20,000* (~ USD 292).*"* (17 years old female TB patient on medication)

> *"When I had chest pain, ASHA told me that we'll have to go to the PHC, tests will be free, and I'll get return fare from my home. I thought I'll get better and decided to go. I got a blood test and x-ray done. Reports were normal and I got some medicines. I felt better… If you know the hospital and workers there, it is easier to get things done. Else, you have to spend a lot of time."* (30 years old female patient)

The ASHAs and the Field Coordinators (FCs) expressed transport allowance, incentive to ASHA, positive patient experience, and patient accompaniment as enablers. (Figure 3)

For peer review only

For peer review only - http://bmjopen.bmj.com/site/about/guidelines.xhtml

"If one patient becomes better, she also advises others. Even if we don't know, the patient who becomes better asks their ill neighbor to call the ASHA for everything. Our mobile number is available in every household." (ASHA)

"Sometimes, patients do not have any shared conveyance from their home. In such cases, patients are unable to come, and transport allowance or hiring an autorickshaw for, say 10 patients of the village, really helps. That way, they get consultation and lab tests at PHC, then chest x-ray at another diagnostic facility, and get dropped at their home." (Field coordinator)

"ASHA's income is from incentives, and if she gets incentives in time, she takes up those activities." (Field coordinator)

barriers to access the first diagnostic evaluation

Barriers were coded from the interview and FGD transcripts into 5 categories and 27 codes. These barriers are listed in the Figure 3 and described below.

Category 1: Logistics-related

As per patients, public transport was not available in certain locations and some patients mentioned their inability to pay for it even where it was available. They also reported not knowing the location of the diagnostic center.

"I don't know where the diagnostic center is. From my village, I have to walk for 3 – 4 km before I find any transport." (65 years old woman)

The ASHA and FC corroborated that transport and insufficient support for certain patient population like disabled people was a key logistics-related challenge.

"Travel options are also limited for some areas. Either it is not readily available or you have to walk before anything can be found. They don't have the money to reserve an entire shared vehicle. If the patient is disabled or elderly, they face even more problems." (Field coordinator)

For peer review only - http://bmjopen.bmj.com/site/about/guidelines.xhtml

Category 2: Health system-related

According to patients, the care at PHC was slow and took a lot of time. They said either the PHC remained closed or they didn't reach the PHC in working hours. At other times when they reached, the providers were not available at the facility.

> *"I had to go 2-3 times to the PHC before all the tests were completed and reports arrived. Only then I could get the medicines."* (17 years old female patient)

> *"We have to run-around a lot in the government hospital. There are huge buildings and it is difficult to figure out what happens where alone."* (30 years old male patient)

The health workers reported unavailability of some diagnostic services in the PHC and the high cost of getting these in the private sector. ASHA's remuneration for providing TB care services was irregular and unpaid. They indicated that poor patient experience at the PHC also deterred others and that the public put a stronger faith in the private sector.

> *"Patient doesn't know where the registration desk is, where the doctor sits, where the labs and x-ray are done. We have to go with them to get everything completed quickly."* (ASHA)

> *"I have provided treatment to 20 patients, but I haven't been paid for it. It is only in the recent 1-2 years that there has been some improvement. We are not told properly how to fill the document and what to submit for release of payments. All our papers were taken and nothing happened. Earlier we used to only counsel the patient to go for diagnostics, but now we come with them if we get money for helping in diagnosis."* (ASHA)

> *"If patient come on a given day and their work doesn't get done, they won't come again. Coming again will mean foregoing another day's wage. They will say that ASHA cheated them and nothing happened at the hospital and nothing is available at the PHC ever. If they go to a private clinic, everything is done in one day and they get the medicine… They also prefer private." (ASHA)*

For peer review only

Category 3: Health provider-related

Patients described health worker strikes in the field and at the PHC as a barrier. One patient also reported that ASHA doesn't come to their home.

> *"I took the sputum to the PHC, but nothing was done. PHC was on strike for 15 days. The unabated cough and fever didn't let me sleep. When I felt that I would die, I took an injection from the RMP and drank 5 cough syrups. Only then was there any relief."* (65 years old male patient)

> *"What will happen after going to the PHC? I go to the hospital and come back with nothing. Even ASHA doesn't come. I am going to lose everything in this hustle and bustle."* (35 years old female patient)

The health workers reported RMPs misguiding the patients, low morale of the workers, and poor health provider behavior at the PHC as barriers.

> *"RMPs misguide the patients a lot. They give medications and injections without any concern. They are not at risk even if the patient dies. If patient becomes serious and they have made money, they will refer them to hospital. They roam through the villages and people get medicine at their doorstep. Someone who is a laborer, their income for the day is saved."* (ASHA)

> *"When we take the patient to the PHC, lab technician will return the sample and asks to come on another day... Doctor will also not write any medication and don't know the updated guidelines… We have to then listen from our patient."* (ASHA)

Category 4: Family-related

Patients indicated not knowing the extent of available services in the PHS. Some described severe indebtedness of the family and not having any money for healthcare. According to them, insufficient family support and absence of a caretaker in the family were also barriers.

> *"I live with my two granddaughters and my son works as a watchman in New Delhi. The money is irregular. If I've no money, I won't go. In November, I didn't have money and*

For peer review only - http://bmjopen.bmj.com/site/about/guidelines.xhtml

> *couldn't go… For consulting with the RMP, I had to take a loan of ₹150* (~ USD 2.2). *After a week, I've been able to return only ₹50."* (65 years old female patient)

> *"Both my kids are alone. My husband has migrated for work. He says that take the kids to a senior doctor in a private hospital. It'll cost at least ₹3000* (~ USD 44) *and I am waiting for it… There is only my old mother-in-law in the house besides me. How will I leave them alone at home?"* (Mother of a 6 years old patient)

Health workers mentioned permission from the head of the family to visit facility, restricted mobility of women in the families as compared to men, and family's concern only if symptoms were severe as barriers.

> *"Patients say that we'll ask the head of the family and come only if they assent. Sometimes they are not sure of the quality of care in the PHS. We also counsel the guardian… This happens more often for women. Men are independent; they don't have to ask the head or the wife… It is also difficult for women. They have to complete household chores and care for the kids before leaving home for the facility. Often, by the time they reach, the facility will close."* (ASHA)

> *"If the symptoms are not severe, then patient takes some medicine from RMP at their home and doesn't want to come for a test."* (Field coordinator)

Category 5: Patient-related

Patients indicated denial of suffering from TB.

> *"I don't need any diagnostics. I didn't have TB ever in my family. This is all because of gas."* (65 years old patient)

As per health workers, some patients had a fear of TB. They also reported discrimination by the community against TB patients.

> *"We don't say TB at first. If we say it, people hesitate. Some are offended that we have said TB is a possible disease for them. We've to tell them gently. One sputum positive patient said that she was hurt that I said she has TB."* (ASHA)

For peer review only

For peer review only - http://bmjopen.bmj.com/site/about/guidelines.xhtml

"A lot of people prefer to keep their disease status hidden. TB is considered contagious and people don't want neighbors to know of it. Earlier, even patient's utensils and bedding were kept separate." (ASHA)

DISCUSSION

This is the first study to assess the magnitude of the pre-diagnostic LFU in an ACF program along with the barriers and enablers in undergoing a diagnostic evaluation. In our study, nearly 44% of the presumptive TB cases didn't undergo diagnostic evaluation. Provision of transport allowance for patients to travel for diagnostics, accompaniment and support of ASHA, incentives to ASHA, and word-of-mouth recommendation of satisfied patients emerged as key enablers. The major barriers were challenges in transport despite an allowance, insufficient family and health provider support, inadequate health services, and poor faith in the public sector.

There are strengths of this study. First, we used a mixed-methods design in which the quantitative and qualitative components complemented each other. The qualitative component explored hitherto unresearched reasons for the pre-diagnostic LFU and recorded the perspectives of various stakeholders in the diagnostic process, including patients and CHWs. Second, the study was conducted in an ACF program using routinely collected data and reflects the on-ground reality. Finally, we adhered to the reporting standards: Strengthening the Reporting of OBservational Studies in Epidemiology (STROBE) guidelines for the quantitative part and the COnsolidated criteria for REporting Qualitative research (COREQ) guidelines for qualitative part.[25,26]

There are several limitations as well. First, the study was conducted in a designed ACF program with interventions across the TB care cascade. The site was different than a typical RNTCP implementation and multiple program staff worked on it in addition to the CHWs and RNTCP staff. In addition, the PHS was comparatively weaker in the study region. Second, there was intuitive screening taking place before the patients entered the care cascade. Therefore, the true number of people being screened was not known. Third, we only recorded presumptive TB cases undergoing diagnostic evaluation and there was no recording of presumptive TB cases reaching facility but not undergoing evaluation. Fourth, the yield in terms of confirmed TB diagnosis was

For peer review only

For peer review only - http://bmjopen.bmj.com/site/about/guidelines.xhtml

known, but we didn't record the number of people completing the diagnostic process as per the diagnostic algorithm. Fifth, we missed presumptive TB cases accessing TB diagnostic evaluation in the private sector.

The study from the ACF program in Myanmar reported 96.6% of the presumptive TB cases receiving CXR in the mobile van, while only 51.4% of the cases referred for sputum microscopy to the health facility followed through. Immediate availability of a mobile CXR machine was a key factor behind such high rates, and lack of a one-stop diagnostic service was a disincentive for patients.[12] Project Axshya in India reported a low 22% people going to the DMC out of all those referred, while overall 54% received sputum microscopy through sputum transport and referral to DMC combined.[13] In contrast, 41.1% of the presumptive TB cases in our study went to a facility for a diagnostic evaluation. Our qualitative findings corroborate it and suggest that difficulty in transport and poor patient experience are important barriers. A one-stop care center increases the service uptake and patients don't have to make repeated visits. Similarly, sputum transport mechanisms reduce patient's hardship. Accompaniment and support of ASHA is a likely reason for this difference with the previous Indian finding.

The Zimbabwe study reported place of residence (rural) and type of facility (private) as important risk factor for pre-diagnostic LFU.[14] While we found association of LFU with various risk factors, their importance in public health programs is limited. However, the qualitative findings point towards important reasons. Previously known challenges in TB programs like TB stigma, patient denial, poor patient experience, and health system deficiencies resonated in our findings. Patients found care seeking in public sector cumbersome, and the informal providers and private sector remained a trusted first choice for most. While there were no significant differences in LFU between the genders, women reported additional challenges of restricted mobility, financial dependence, and burden of household work.

The study has implications for various public health programs targeting improved TB care services, particularly in poor and rural populations. In fact, the barriers in accessing a diagnostic evaluation have a bearing on the access to a broader range of primary healthcare services in similar settings. Learnings from enablers and addressing barriers can improve uptake of the diagnostic services and change the perception of the PHS. We recommend strong patient support

For peer review only - http://bmjopen.bmj.com/site/about/guidelines.xhtml

mechanism across the TB care pathway, involvement of families in TB care, and systematic reform to address health system-related challenges. Further research in health system challenges involving the perspectives of health system administrators, doctors, nurses, and other health facility workers will inform the process of such a reform.

In conclusion, about three-fifths of the presumptive TB cases were LFU in pre-diagnostic stage and didn't have a TB diagnostic test. Several enablers and barriers for diagnostic evaluation were identified. If we want to find the missing TB patients, it needs to be easier for presumptive TB patients to navigate the diagnostic process. Health system reforms are rather complex, however, rather simple interventions such as sputum transportation and adequate support from a person knowing the health system may already make big changes.

ACKNOWLEDGEMENT

We are grateful for the support of RNTCP, Government of Bihar, State TB Officer Dr. (Major) K. N. Sahai, Communicable Disease Officer Dr. Sree Ram Prasad, and District Health Society, Samastipur. We thank Sriram Selvaraju, Anthony D. Harries, Dick Menzies, and participants of the TB Research Methods course at McGill Summer Institute 2018 for their comments on the protocol, and Amol Dongre for his feedback on qualitative results. The program couldn't have been implemented without the tireless efforts of ASHAs, health providers, and the team at IIH.

This research was conducted through the Structured Operational Research and Training Initiative (SORT IT), a global partnership led by the Special Programme for Research and Training in Tropical Diseases at the World Health Organization (WHO/TDR). The model is based on a course developed jointly by the International Union Against Tuberculosis and Lung Disease (The Union) and Medécins sans Frontières (MSF/Doctors Without Borders). The specific SORT IT programme which resulted in this publication was jointly developed and implemented by: The Union South-East Asia Office, New Delhi, India; the Centre for Operational Research, The Union, Paris, France; Department of Preventive and Social Medicine, Jawaharlal Institute of Postgraduate Medical Education and Research, Puducherry, India; Deartment of Community Medicine and School of Public Health, Postgraduate Institute of Medical Education and Research, Chandigarh, India; Department of Community Medicine, All India Institute of Medical Sciences, Nagpur, India; Dr.

For peer review only - http://bmjopen.bmj.com/site/about/guidelines.xhtml

Rajendra Prasad Centre for Ophthalmic Sciences, All India Institute of Medical Sciences, New Delhi, India; Department of Community Medicine, Pondicherry Institute of Medical Science, Puducherry, India; Department of Community Medicine, Kalpana Chawla Medical College, Karnal, India; National Centre of Excellence and Advance Research on Anemia Control, All India Institute of Medical Sciences, New Delhi, India; Department of Community Medicine, Sri Manakula Vinayagar Medical College and Hospital, Puducherry, India; Department of Community Medicine, Velammal Medical College Hospital and Research Institute, Madurai, India; Department of Community Medicine, Yenepoya Medical College, Mangalore, India; Karuna Trust, Bangalore, India and National Institute for Research in Tuberculosis, Chennai, India.

COMPETING INTERESTS

None declared

FUNDING

This project was supported by the Stop TB Partnership's TB REACH initiative and was funded by the Government of Canada and the Bill & Melinda Gates Foundation. Manish Bhardwaj and Dyuti Sen are supported by a Grand Challenges Explorations grant number OPP1190735 and OPP1190905 respectively from the Bill & Melinda Gates Foundation. The training program, within which this paper was developed, was funded by the Department for International Development (DFID), UK. No funders had any role in study design, data collection and analysis, decision to publish, or preparation of the manuscript.

DATA AVAILABILITY

The deidentified data used in the study is available from the corresponding author on reasonable request. It contains information on patient demographics and characteristics, screening criteria, diagnostic test used, and final diagnosis. There is no additional data available beyond that.

For peer review only

ETHICS APPROVAL

Ethical approval was obtained from Institutional Ethics Committee, Emmanuel Hospital Association, New Delhi (Order 201) and the Ethics Advisory Group, International Union Against Tuberculosis and Lung Disease, Paris (EAG number 98/18). Informed consent was obtained from participants before the in-depth interviews and FGDs.

AUTHOR CONTRIBUTION

Conceptualization and design: TG, VG, DS, MV, MiB, RM, MaB

Data collection: TG, DS, RM

Data analysis and interpretation: TG, VG, DS, RM

Writing — original draft: TG

Writing — review and edit: TG, VG, DS, MV, MiB, RM, MaB

REFERENCES

1 World Health Organization. Gear up to end TB: introducing the end TB strategy. World Health Organization 2015.

2 Global tuberculosis report. World Health Organization 2017. http://www.who.int/tb/publications/global_report/en/ (accessed 6 Sep 2018).

3 World Health Organization. *Global tuberculosis report 2018*. Geneva: : World Health Organization 2018. http://www.who.int/iris/handle/10665/274453 (accessed 18 Sep 2018).

4 Subbaraman R, Nathavitharana RR, Mayer KH, *et al.* Constructing care cascades for active tuberculosis: A strategy for program monitoring and identifying gaps in quality of care. *PLOS Medicine* 2019;**16**:e1002754. doi:10/gf38sz

5 Subbaraman R, Nathavitharana RR, Satyanarayana S, *et al.* The Tuberculosis Cascade of Care in India's Public Sector: A Systematic Review and Meta-analysis. *PLOS Medicine* 2016;**13**:e1002149. doi:10/f9b29f

6 Naidoo P, Theron G, Rangaka MX, *et al.* The South African Tuberculosis Care Cascade: Estimated Losses and Methodological Challenges. *J Infect Dis* 2017;**216**:S702–13. doi:10/gcjz4v

7 Lönnroth K, Corbett E, Golub J, *et al.* Systematic screening for active tuberculosis: rationale, definitions and key considerations [State of the art series. Active case finding/screening.

For peer review only - http://bmjopen.bmj.com/site/about/guidelines.xhtml

Number 1 in the series]. *The International Journal of Tuberculosis and Lung Disease* 2013;**17**:289–98. doi:10/gd52dz

8 Storla DG, Yimer S, Bjune GA. A systematic review of delay in the diagnosis and treatment of tuberculosis. *BMC Public Health* 2008;**8**:15. doi:10/c6sz7r

9 Wells WA. Onions and prevalence surveys: how to analyze and quantify tuberculosis case-finding gaps. *The International Journal of Tuberculosis and Lung Disease* 2017;**21**:1101–13. doi:10/gb4g9c

10 Creswell J, Sahu S, Blok L, *et al.* A Multi-Site Evaluation of Innovative Approaches to Increase Tuberculosis Case Notification: Summary Results. *PLOS ONE* 2014;**9**:e94465. doi:10/gcz84n

11 Mhimbira FA, Cuevas, LE, Dacombe, R, Mkopi, A, Sinclair D. Interventions to increase tuberculosis case detection at primary healthcare or community-level services. *Cochrane Database of Systematic Reviews* Published Online First: 2017. doi:10/gcmjrv

12 Myint O, Saw S, Isaakidis P, *et al.* Active case-finding for tuberculosis by mobile teams in Myanmar: yield and treatment outcomes. *Infectious Diseases of Poverty* 2017;**6**:77. doi:10/gf38nz

13 Prasad BM, Satyanarayana S, Chadha SS, *et al.* Experience of active tuberculosis case finding in nearly 5 million households in India. *Public Health Action* 2016;**6**:15–8. doi:10/gd4v8q

14 Padingani M, Kumar A, Tripathy JP, *et al.* Does pre-diagnostic loss to follow-up among presumptive TB patients differ by type of health facility? An operational research from Hwange, Zimbabwe in 2017. *Pan African Medical Journal* 2018;**31**. doi:10.11604/pamj.2018.31.196.15848

15 Creswell JW, Clark VLP. *Designing and Conducting Mixed Methods Research*. SAGE 2011.

16 Registrar General I. Census of India 2011: provisional population totals-India data sheet. *Office of the Registrar General Census Commissioner, India Indian Census Bureau* 2011.

17 Annual Health Survey 2011-12, Bihar Factsheet. New Delhi: : Office of the Registrar General & Census Commissioner http://www.censusindia.gov.in/vital_statistics/AHSBulletins/AHS_Factsheets_2011_12/Bihar_Factsheet_2011-12.pdf (accessed 3 Feb 2019).

18 Central TB Division. India TB Report 2018: Revised National Tuberculosis Control Program - Annual Status Report. New Delhi, India: : Directorate General of Health Services, Ministry of Health and Family Welfare, Government of India 2018. https://tbcindia.gov.in/showfile.php?lid=3314

19 National Informatics Centre, Patna. Health Department Geographical Information System, Bihar. http://gis.bih.nic.in/Depts/Health.aspx (accessed 14 Sep 2018).

For peer review only

For peer review only - http://bmjopen.bmj.com/site/about/guidelines.xhtml

20 Update on ASHA Programme. New Delhi: : National Health Mission, Ministy of Health and Family Welfare 2018. http://nhsrcindia.org/sites/default/files/asha%20reduced.pdf (accessed 29 Jul 2019).

21 Revised Technical & Operational Guidelines for Tuberculosis Control in India. New Delhi: : Central TB Division 2016. https://www.tbcindia.gov.in/WriteReadData/Revised%20Technical%20and%20Operational%20Guidelines/files/assets/basic-html/page-1.html# (accessed 4 Feb 2019).

22 Integrated Child Development Services Scheme. Ministry of Women and Child Development, Government of India. https://icds-wcd.nic.in/icds.aspx (accessed 17 Aug 2019).

23 Attride-Stirling J. Thematic networks: an analytic tool for qualitative research. *Qualitative research* 2001;**1**:385–405. doi:10/cg4grf

24 Lincoln Y, Guba E. Naturalistic Inquiry: Designing a naturalistic inquiry. *Beverly Hills: Sage Publications* 1985.

25 von Elm E, Altman DG, Egger M, *et al.* The Strengthening the Reporting of Observational Studies in Epidemiology (STROBE) statement: guidelines for reporting observational studies. *Lancet* 2007;**370**:1453–7.

26 Tong A, Sainsbury P, Craig J. Consolidated criteria for reporting qualitative research (COREQ): a 32-item checklist for interviews and focus groups. *International journal for quality in health care* 2007;**19**:349–357. doi:10/fm8b7v

LEGEND FOR FIGURES

Figure 1: Operational definitions used in the program

CHW: Community Health Worker

ASHA: Accredited Social Health Activist

AWW: Anganwadi Worker

CXR: Chest X-ray

USG: Ultrasonography

For peer review only - http://bmjopen.bmj.com/site/about/guidelines.xhtml

561 **Figure 2: TB care cascade between January 2018 and December 2018 from community referral**
562 **to diagnostic evaluation in a community-based ACF program in Samastipur, India**

563 All percentages are calculated as a proportion of the number of participants entering the previous
564 step of the cascade.

565 * Includes 140 Presumptive TB cases undergoing diagnostic evaluation beyond 30 days from
566 screening.

567 **Figure 3: The enablers and barriers in diagnostic evaluation from patients', ashas', and field**
568 **coordinators' perspective in an ACF TB program in Samastipur, India from January 2018 to**
569 **December 2018**

570 [a]: responses of patient

571 [b]: responses of ASHA

572 [c]: responses of FC

573 ASHA: Accredited Social Health Activist

574 PHS: Public Health System

575 PHC: Primary Health Centre

For peer review only

For peer review only - http://bmjopen.bmj.com/site/about/guidelines.xhtml

Term	Definition
Community referral	A person at risk of TB referred for TB screening to the program by a CHW (ASHA or AWW) or an informal provider or community during their routine work
Presumptive TB patient	A person identified with one or more of the following symptoms of TB in the screening process at their home: cough ≥ 2 weeks; sputum in cough, hemoptysis in last 6 months; chest pain in last 1 month; fever ≥ 2 weeks; night sweats ≥ 2 weeks; severe weight loss in last 3 months; swelling in a lymph node
Diagnostic evaluation	A presumptive TB patient tested using one or more of a sputum microscopy or CXR or GeneXpert or extrapulmonary TB testing (USG or FNAC) within 30 days of screening
Pre-diagnostic loss to follow-up	A presumptive TB patient not undergoing a diagnostic evaluation

For peer review only - http://bmjopen.bmj.com/site/about/guidelines.xhtml

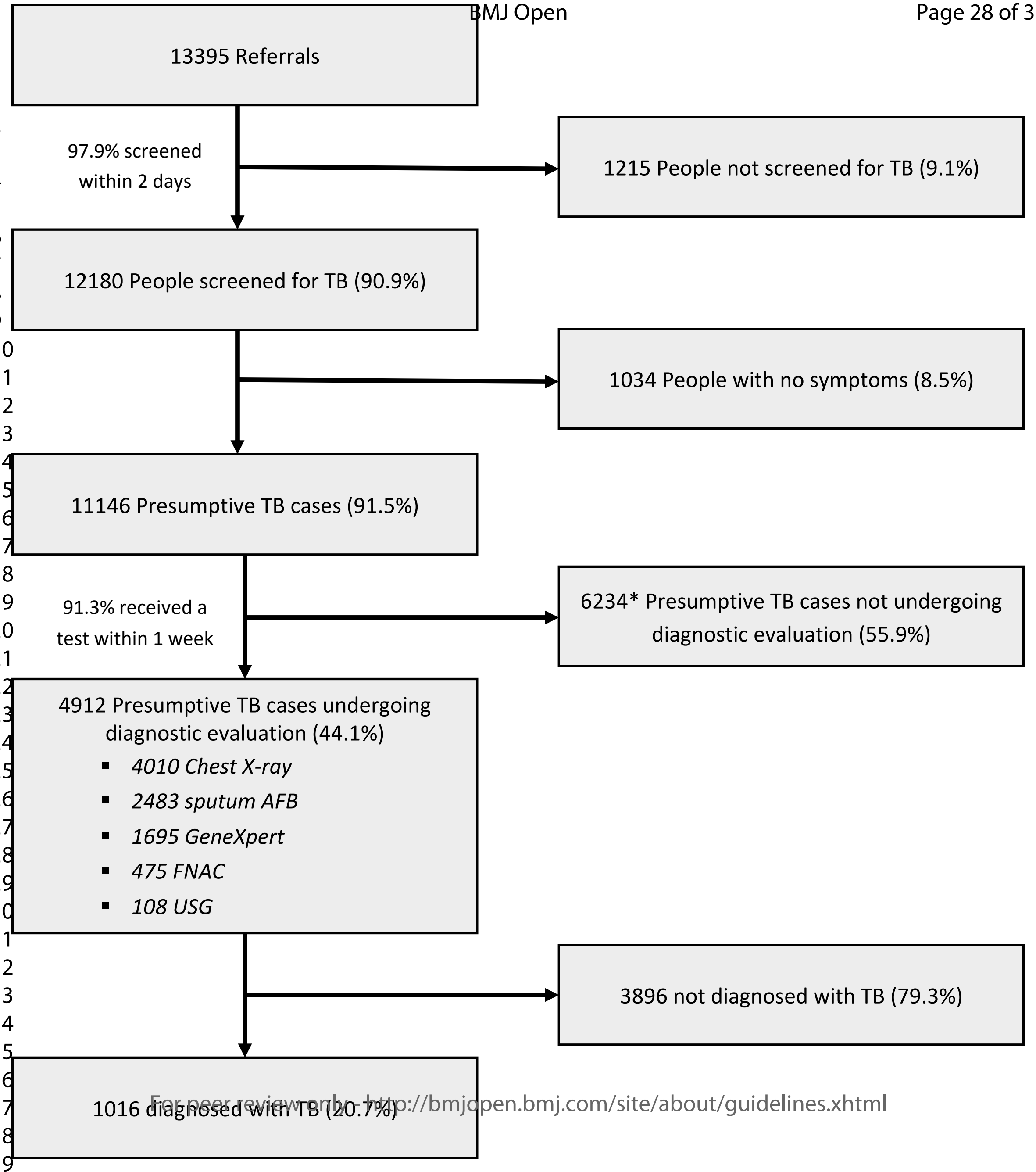

For peer review only - http://bmjopen.bmj.com/site/about/guidelines.xhtml

Enablers

Category 1: Logistics-related
- Transport allowance [a,b,c]

Category 2: Health system-related
- Free services in the PHS [a,b]
- Knowledge of the PHS procedures [a]
- Incentive to ASHA for TB work is vital [b,c]
- Previous positive patient experience in PHS [b,c]

Category 3: Health provider-related
- ASHA's assistance in the diagnostic process [a]
- ASHA's accompaniment to the health facility [a,c]
- ASHA's understanding of the PHS functioning [a]
- Satisfied people referred others to ASHA [b]

Barriers

Category 1: Logistics-related
- Unavailability of public transport from patient's village [a,c]
- No knowledge of the diagnostic center location [a]
- No transport support for the disabled patient [b]

Category 2: Health system-related
- Care at PHC was slow and time-taking [a,b,c]
- Not all diagnostics were available in the PHC [b]
- High cost of services not available in the PHC [b]
- Difficult to understand PHC system [a,b]
- PHC remained closed [a]
- Inability to reach PHC in working hours [a,b]
- Provider was unavailable at the PHC [a]
- Irregular and unpaid ASHA incentives [b]
- Faith in the private sector [b,c]
- Poor patient experience deterred others as well [b]

Category 3: Health provider-related
- Informal providers misguided the patient [b,c]
- Low morale of the health providers [b]
- Health worker strikes [a]
- Poor health provider behavior at PHC [b,c]
- ASHA didn't come to the patient's home [a]

Category 4: Family-related
- Poor awareness of TB care in PHS [a,c]
- Family concerned only when symptoms were severe [b]
- Insufficient family support [a]
- No caretaker in the family to help in reaching hospital [a]
- Family permission was necessary [b]
- Indebted, and no money for healthcare [a]
- Restricted mobility of women [b]

Category 5: Patient-related
- Denial of suffering from TB [a]
- Fear of TB [b]
- Discrimination against diseased patient [b,c]

For peer review only - http://bmjopen.bmj.com/site/about/guidelines.xhtml

COREQ (COnsolidated criteria for REporting Qualitative research) Checklist

A checklist of items that should be included in reports of qualitative research. You must report the page number in your manuscript where you consider each of the items listed in this checklist. If you have not included this information, either revise your manuscript accordingly before submitting or note N/A.

Topic	Item No.	Guide Questions/Description	Reported on Page No.
**Domain 1: Research team and reflexivity**			
Personal characteristics			
Interviewer/facilitator	1	Which author/s conducted the interview or focus group?	
Credentials	2	What were the researcher's credentials? E.g. PhD, MD	
Occupation	3	What was their occupation at the time of the study?	
Gender	4	Was the researcher male or female?	
Experience and training	5	What experience or training did the researcher have?	
Relationship with participants			
Relationship established	6	Was a relationship established prior to study commencement?	
Participant knowledge of the interviewer	7	What did the participants know about the researcher? e.g. personal goals, reasons for doing the research	
Interviewer characteristics	8	What characteristics were reported about the inter viewer/facilitator? e.g. Bias, assumptions, reasons and interests in the research topic	
**Domain 2: Study design**			
Theoretical framework			
Methodological orientation and Theory	9	What methodological orientation was stated to underpin the study? e.g. grounded theory, discourse analysis, ethnography, phenomenology, content analysis	
Participant selection			
Sampling	10	How were participants selected? e.g. purposive, convenience, consecutive, snowball	
Method of approach	11	How were participants approached? e.g. face-to-face, telephone, mail, email	
Sample size	12	How many participants were in the study?	
Non-participation	13	How many people refused to participate or dropped out? Reasons?	
Setting			
Setting of data collection	14	Where was the data collected? e.g. home, clinic, workplace	
Presence of non-participants	15	Was anyone else present besides the participants and researchers?	
Description of sample	16	What are the important characteristics of the sample? e.g. demographic data, date	
Data collection			
Interview guide	17	Were questions, prompts, guides provided by the authors? Was it pilot tested?	
Repeat interviews	18	Were repeat inter views carried out? If yes, how many?	
Audio/visual recording	19	Did the research use audio or visual recording to collect the data?	
Field notes	20	Were field notes made during and/or after the inter view or focus group?	
Duration	21	What was the duration of the inter views or focus group?	
Data saturation	22	Was data saturation discussed?	
Transcripts returned	23	Were transcripts returned to participants for comment and/or	

For peer review only

Topic	Item No.	Guide Questions/Description	Reported on Page No.
		correction?	
**Domain 3: analysis and findings**			
Data analysis			
Number of data coders	24	How many data coders coded the data?	
Description of the coding tree	25	Did authors provide a description of the coding tree?	
Derivation of themes	26	Were themes identified in advance or derived from the data?	
Software	27	What software, if applicable, was used to manage the data?	
Participant checking	28	Did participants provide feedback on the findings?	
Reporting			
Quotations presented	29	Were participant quotations presented to illustrate the themes/findings? Was each quotation identified? e.g. participant number	
Data and findings consistent	30	Was there consistency between the data presented and the findings?	
Clarity of major themes	31	Were major themes clearly presented in the findings?	
Clarity of minor themes	32	Is there a description of diverse cases or discussion of minor themes?	

Developed from: Tong A, Sainsbury P, Craig J. Consolidated criteria for reporting qualitative research (COREQ): a 32-item checklist for interviews and focus groups. *International Journal for Quality in Health Care*. 2007. Volume 19, Number 6: pp. 349 – 357

Once you have completed this checklist, please save a copy and upload it as part of your submission. DO NOT include this checklist as part of the main manuscript document. It must be uploaded as a separate file.

For peer review only

STROBE 2007 (v4) Statement—Checklist of items that should be included in reports of *cohort studies*

Section/Topic	Item #	Recommendation	Reported on page #
**Title and abstract**	1	(*a*) Indicate the study's design with a commonly used term in the title or the abstract	1
		(*b*) Provide in the abstract an informative and balanced summary of what was done and what was found	3, 4
**Introduction**			
Background/rationale	2	Explain the scientific background and rationale for the investigation being reported	5, 6
Objectives	3	State specific objectives, including any prespecified hypotheses	5, 6
**Methods**			
Study design	4	Present key elements of study design early in the paper	6
Setting	5	Describe the setting, locations, and relevant dates, including periods of recruitment, exposure, follow-up, and data collection	6 – 8
Participants	6	(*a*) Give the eligibility criteria, and the sources and methods of selection of participants. Describe methods of follow-up	8
		(*b*) For matched studies, give matching criteria and number of exposed and unexposed	Not applicable
Variables	7	Clearly define all outcomes, exposures, predictors, potential confounders, and effect modifiers. Give diagnostic criteria, if applicable	8, 9
Data sources/ measurement	8*	For each variable of interest, give sources of data and details of methods of assessment (measurement). Describe comparability of assessment methods if there is more than one group	8, 9
Bias	9	Describe any efforts to address potential sources of bias	9, 10
Study size	10	Explain how the study size was arrived at	8
Quantitative variables	11	Explain how quantitative variables were handled in the analyses. If applicable, describe which groupings were chosen and why	9, 10
Statistical methods	12	(*a*) Describe all statistical methods, including those used to control for confounding	9, 10
		(*b*) Describe any methods used to examine subgroups and interactions	9, 10
		(*c*) Explain how missing data were addressed	9
		(*d*) If applicable, explain how loss to follow-up was addressed	5 – 7, 9
		(*e*) Describe any sensitivity analyses	Not Applicable
**Results**			

For peer review only

Participants	13*	(a) Report numbers of individuals at each stage of study—eg numbers potentially eligible, examined for eligibility, confirmed eligible, included in the study, completing follow-up, and analysed	10 – 12
		(b) Give reasons for non-participation at each stage	10 – 17
		(c) Consider use of a flow diagram	Figure 2
Descriptive data	14*	(a) Give characteristics of study participants (eg demographic, clinical, social) and information on exposures and potential confounders	10 – 12
		(b) Indicate number of participants with missing data for each variable of interest	9
		(c) Summarise follow-up time (eg, average and total amount)	Figure 2
Outcome data	15*	Report numbers of outcome events or summary measures over time	Figure 2
Main results	16	(*a*) Give unadjusted estimates and, if applicable, confounder-adjusted estimates and their precision (eg, 95% confidence interval). Make clear which confounders were adjusted for and why they were included	Table 2
		(*b*) Report category boundaries when continuous variables were categorized	Table 2
		(*c*) If relevant, consider translating estimates of relative risk into absolute risk for a meaningful time period	Not Applicable
Other analyses	17	Report other analyses done—eg analyses of subgroups and interactions, and sensitivity analyses	Not Applicable
**Discussion**			
Key results	18	Summarise key results with reference to study objectives	17 – 18
**Limitations**			
Interpretation	20	Give a cautious overall interpretation of results considering objectives, limitations, multiplicity of analyses, results from similar studies, and other relevant evidence	19
Generalisability	21	Discuss the generalisability (external validity) of the study results	19
**Other information**			
Funding	22	Give the source of funding and the role of the funders for the present study and, if applicable, for the original study on which the present article is based	21

*Give information separately for cases and controls in case-control studies and, if applicable, for exposed and unexposed groups in cohort and cross-sectional studies.

Note: An Explanation and Elaboration article discusses each checklist item and gives methodological background and published examples of transparent reporting. The STROBE checklist is best used in conjunction with this article (freely available on the Web sites of PLoS Medicine at http://www.plosmedicine.org/, Annals of Internal Medicine at http://www.annals.org/, and Epidemiology at http://www.epidem.com/). Information on the STROBE Initiative is available at www.strobe-statement.org.

For peer review only

BMJ Open

Pre-diagnostic loss to follow-up in an active case finding TB program: a mixed-methods study from rural Bihar, India

Journal:	*BMJ Open*
Manuscript ID	bmjopen-2019-033706.R1
Article Type:	Original research
Date Submitted by the Author:	09-Dec-2019
Complete List of Authors:	Garg, Tushar; Innovators In Health, Research<br>Gupta, Vivek; All India Institute of Medical Sciences, Dr. R.P Centre for Ophthalmic Sciences<br>Sen, Dyuti; Innovators In Health, Operations<br>Verma, Madhur ; All India Institute of Medical Sciences, Department of Community and Family Medicine<br>Brouwer, Miranda; PHTB Consult, Consulting<br>Mishra, Rajeshwar; Innovators In Health, Research; Centre for Development of Human Initiatives<br>Bhardwaj, Manish; Innovators In Health, Operations
**Primary Subject Heading**:	Public health
Secondary Subject Heading:	Infectious diseases, Global health
Keywords:	pre-diagnostic loss to follow-up, cascade of care, ASHA, Accredited Social Health Activist, Community health worker, screening

SCHOLARONE™
Manuscripts

I, the Submitting Author has the right to grant and does grant on behalf of all authors of the Work (as defined in the below author licence), an exclusive licence and/or a non-exclusive licence for contributions from authors who are: i) UK Crown employees; ii) where BMJ has agreed a CC-BY licence shall apply, and/or iii) in accordance with the terms applicable for US Federal Government officers or employees acting as part of their official duties; on a worldwide, perpetual, irrevocable, royalty-free basis to BMJ Publishing Group Ltd ("BMJ") its licensees and where the relevant Journal is co-owned by BMJ to the co-owners of the Journal, to publish the Work in this journal and any other BMJ products and to exploit all rights, as set out in our licence.

The Submitting Author accepts and understands that any supply made under these terms is made by BMJ to the Submitting Author unless you are acting as an employee on behalf of your employer or a postgraduate student of an affiliated institution which is paying any applicable article publishing charge ("APC") for Open Access articles. Where the Submitting Author wishes to make the Work available on an Open Access basis (and intends to pay the relevant APC), the terms of reuse of such Open Access shall be governed by a Creative Commons licence – details of these licences and which Creative Commons licence will apply to this Work are set out in our licence referred to above.

Other than as permitted in any relevant BMJ Author's Self Archiving Policies, I confirm this Work has not been accepted for publication elsewhere, is not being considered for publication elsewhere and does not duplicate material already published. I confirm all authors consent to publication of this Work and authorise the granting of this licence.

For peer review only

TITLE

Pre-diagnostic loss to follow-up in an active case finding TB program: a mixed-methods study from rural Bihar, India

MANUSCRIPT TYPE

Research article

AUTHORS

Tushar Garg[1], Vivek Gupta[2], Dyuti Sen[1], Madhur Verma[3], Miranda Brouwer[4], Rajeshwar Mishra[1,5], Manish Bhardwaj[1]

1. Innovators In Health, Patna, India
2. Dr R P Centre for Ophthalmic Sciences, All India Institute of Medical Sciences, New Delhi, India
3. All India Institute of Medical Sciences, Bathinda, India
4. PHTB Consult, Tilburg, The Netherlands
5. Centre for Development of Human Initiatives, New Jalpaiguri, India

ADDRESS FOR CORRESPONDENCE

Tushar Garg,
Innovators In Health, #176, Ground Floor, Road #2,
New Pataliputra Colony, Patna – 800013. Bihar. India.
Email: tgarg@innovatorsinhealth.org
Phone: +91 98286 09562

MANUSCRIPT DETAILS

Abstract: 280 words
Manuscript: 4958 words
Tables: 2 + 1 (supplementary)

For peer review only

For peer review only - http://bmjopen.bmj.com/site/about/guidelines.xhtml

Figures: 3

KEYWORDS

Pre-diagnostic loss to follow-up; cascade of care; screening; ASHA; Accredited Social Health Activist; community health worker

For peer review only

For peer review only - http://bmjopen.bmj.com/site/about/guidelines.xhtml

ABSTRACT

objective

To quantify the pre-diagnostic loss to follow-up (PDLFU) in an active case finding (ACF) TB program, and identify the barriers and enablers in undergoing diagnostic evaluation.

design

Explanatory mixed-methods design.

setting

A rural population of 1.02 million in the Samastipur district of Bihar, India.

participants

Based on their knowledge of health status of families, CHW or community health workers (called "ASHA" locally) and informal providers referred people to the program. The field coordinator (FC) in the program screened these referrals for TB symptoms to identify presumptive TB cases. CHWs accompanied the presumptive TB patients to free diagnostic evaluation and a transport allowance was given to the patients. CHWs initiated and supported the treatment of confirmed cases. We included 13395 community referrals received from January 2018 to December 2018. We conducted in-depth interviews with patients who were evaluated (n = 3), patients not evaluated (n = 4), and focus group discussions with the CHWs (n = 2) and FCs (n = 1).

outcome measures

Proportion and characteristics of PDLFU, and association of demographic and symptom characteristics with diagnostic evaluation.

results

A total of 11146 presumptive TB cases were identified from January 2018 to December 2018, out of which 4912 (44.1%) underwent diagnostic evaluation. The key enablers were CHW accompaniment and support in addition to the free TB services in the public sector. The major

For peer review only - http://bmjopen.bmj.com/site/about/guidelines.xhtml

barriers identified were misinformation and stigma, deficient family and health provider support, transport challenges, and poor services in the public health system.

conclusion

Finding the missing cases will require urgent reform in the health system and patient-centric diagnostic services. However, a community-oriented intervention focusing on stigma, misinformation, and patient support will be critical to its success.

STRENGTHS AND LIMITATIONS OF THIS STUDY

1. First such study to explore the reasons for pre-diagnostic loss to follow-up
2. A mixed-method design including the views of both patients and community health workers
3. Uses operational data from a routine programmatic setting at an NGO site
4. Since intervention removed some barriers, all findings are not necessarily generalizable.
5. No record of the actual number of people screened before being referred to the program.

For peer review only

For peer review only - http://bmjopen.bmj.com/site/about/guidelines.xhtml

INTRODUCTION

The End TB strategy targets an 80% reduction in TB incidence by 2030.[1] However, as many as 4.1 million (39%) patients globally are not notified, indicating a mix of under-reporting and under-diagnosis.[2] With an incidence rate of 199 per 100,000 population in 2018, India alone has about a million such "missing" cases.[3]

Most TB patients are identified through passive case-finding in which patients reach out to a provider.[4] However, it leads to delays in diagnosis, and loss to follow-up (LFU) during diagnostic and treatment phases.[5] In fact, the Indian public sector cascade estimates a 28% LFU at test access amongst incident cases.[6] The major challenges in diagnosis are poor geographical and financial access to healthcare, and failure to test when people do present at the facility.[7] Active case-finding (ACF) can solve some of these challenges by proactively taking health services to the people in the community.[2,8]

Diverse ACF strategies have demonstrated increased TB case detection with early diagnosis.[4,9–11] Nonetheless, multiple ACF interventions report a high pre-diagnostic loss to follow-up (PDLFU). An Indian study reported only 22% of the people with presumptive TB reaching a microscopy center, while a study in Myanmar estimated only 51.4% of patients with abnormal chest X-ray (CXR) getting their sputum examined.[12,13] Although the quantum of the PDLFU is known, underlying reasons are yet to be investigated.

We studied the PDLFU in a community-based ACF program implemented in India in 2018. The objectives were to assess proportion and characteristics of PDLFU cases, identify the risk factors, and understand the reasons for the PDLFU, including the barriers and enablers in accessing diagnostic evaluation.

METHODS

study design

We used an explanatory mixed-methods study design, where the quantitative phase (cohort analysis) was followed by a qualitative phase (descriptive design).[14]

For peer review only - http://bmjopen.bmj.com/site/about/guidelines.xhtml

study setting

General setting

The study was conducted in the Sarairanjan, Bibhutipur, and Ujiarpur blocks (equivalent to TB Unit or TU) of Samastipur district in Bihar with a combined population of 1,021,483. The highest earning member in 69.8% of the households earned less than INR 60,000 annually (~USD 852) and 63.5% of the population was reported literate.[15] Females had 20% lesser literacy rate compared to males, and infant mortality rate (IMR) per 1000 live births was 60 for females against that of 48 for males.[16] In 2017, the public TB case notification rate in the district was 55 per 100,000 population and the pre-treatment LFU was 25%.[17] Furthermore, patients preferred availing TB services in the private sector.[18]

The population was serviced by 12 primary health centres (PHC) under the PHS.[19] Within the Revised National TB Control Program (RNTCP), 4 designated microscopy centres (DMC) run by laboratory technicians provided sputum microscopy. A Senior Treatment Supervisor (STS) at the TU managed the program and a Senior Treatment Laboratory Supervisor (STLS) managed a group of DMCs.

The TB care services in the community were provided by Accredited Social Health Activist or ASHA. ASHA is a CHW within the National Health Mission (NHM) assigned for every 1000 population. Her role is to be a facilitator, mobilizer, and community service provider for various public health programs.[20] Under the RNTCP, ASHA received an activity-based remuneration of INR 1000 – 1500 (~USD 14 – 21) for drug-sensitive TB treatment support, and INR 5000 (~USD 72) for drug-resistant treatment support.[21]

Anganwadi workers (AWW) are a CHW under the Integrated Child Development Services Scheme and receive a fixed monthly honorarium. Through Angadwadi Centres, they provide early childhood care and education for children up to 6 years. However, they are not involved in TB care.[22]

For peer review only - http://bmjopen.bmj.com/site/about/guidelines.xhtml

Specific setting

Supported by TB REACH, we implemented a novel, community-based ACF project in collaboration with RNTCP and NHM. The ACF was added to the routine RNTCP program with interventions in the community and public health system. Its key components were community referral, symptom-based screening at patient's home, transport allowance to patients, free diagnostic evaluation, and diagnosis and treatment assisted by ASHAs. There were no consistent ACF campaigns prior to this program in our study population.

We engaged various health workers, including ASHA, AWW and RMP (informal providers known as Registered Medical Practitioner). A TB awareness meeting was organized in the community to kickstart the program. The health workers and layperson were asked to refer people who may have TB to the program. Since these workers are aware of the health status of the families in the community, they are well-placed to identify such people during their routine work. These community referrals were reported to a field coordinator (FC) of the project, who screened them at their home for TB symptoms. The presumptive TB cases were those with one or more of the following symptoms: cough of ≥ 2 weeks, sputum in cough, hemoptysis in last 6 months, chest pain in last 1 month, fever of ≥ 2 weeks, night sweats for ≥ 2 weeks, severe weight loss in the last 3 months, swelling in a lymph node.

All presumptive TB cases were tested using sputum microscopy and CXR. Patients with a positive smear, or those with a negative smear but abnormal CXR, or patients with a negative smear but clinically suspect were offered a GeneXpert test using sputum collection and transport. ASHAs assisted presumptive TB patients in reaching the diagnostic centres and accompanied them through the process. Patients received a transport allowance and diagnostic tests were free of cost. If the PHS physician confirmed TB diagnosis, treatment was initiated under ASHA's supervision. The ASHA delivered drugs to the patient, ensured follow-up tests and treatment adherence, and monitored for adverse effects.[Figure 1]

A conditional incentive of INR 200 (~USD 3) against community referral and INR 300 (~USD 5) against diagnosis was given for each confirmed case of TB. These incentives to the CHW were in

For peer review only - http://bmjopen.bmj.com/site/about/guidelines.xhtml

addition to the RNTCP treatment support incentives. The project's FCs received a fixed monthly salary.

The project used a Management Information System (MIS) developed in Microsoft Excel 2016® which maintained individual patient data electronically. On receipt of a community referral, the FC enlisted them in a referral register with a unique ID and a separate form was completed for screening. The status of each community referral was tracked using the referral register. A patient-wise folder with various forms and diagnostic reports was maintained for all presumptive TB cases undergoing diagnostic evaluation. Data were entered into the MIS weekly and at least 20% entries were verified monthly in a two-person formation to assess data entry quality.

study population and study period

Quantitative

All community referrals in the 3 selected blocks in Samastipur district between January 01, 2018 to December 31, 2018 were included in the study. There was no sampling involved.

Qualitative

Presumptive TB cases referred for diagnostic evaluation were interviewed. A total of 7 in-depth interviews were conducted with 3 presumptive TB cases who were evaluated (2 females, 1 male, age range: 17 – 30 years) and 4 presumptive cases who were not evaluated (2 females, 2 males, age range: 6 – 65 years). A purposive sample of ASHAs and field coordinators was selected for the focus group discussions (FGD). Three FGDs were conducted with a group of 8 ASHAs (all females, age range: 24 – 49), 11 ASHAs (all females, age range: 22 – 54), and 9 field coordinators (6 females, 3 males, age range: 20 – 38). The average duration was 36 minutes (range 23 – 51 minutes). A diverse sample of males and females across age groups was purposively selected for diversity and interviewed till saturation was achieved.

For peer review only - http://bmjopen.bmj.com/site/about/guidelines.xhtml

166 **data variables, sources of data and data collection**

167 Quantitative

168 Data on the community referrals' characteristics (location, referral source, age, gender), 169 screening criteria (date of screening, symptoms, outcome), and diagnostic evaluation (date of 170 test, type of test) were extracted from the MIS into a structured proforma. The data extracted 171 from the MIS was deidentified before being exported for analysis. [Figure 1]

172 Qualitative

173 Presumptive TB cases were interviewed by TG (a male medical doctor trained in qualitative 174 research) and DS (a female economist trained in qualitative research), and FGDs were conducted 175 by TG and RM (a male professor of social psychology). All the three investigators have adequate 176 experience of the socio-cultural context of the region, and understanding of TB programs. After 177 obtaining a participant's consent, the interviews were conducted at a time and place convenient 178 for the participant and their privacy was ensured. The regional vernacular was used for 179 interactions, and audio-recorded using voice recorder after obtaining consent. The objectives of 180 the study were explained to the participants. No participant denied permission for an interview 181 and there were no repeat interviews. A topic guide was used for FGDs and an interview guide for 182 in-depth interviews to explore the enabling factors and barriers for diagnostic evaluation. 183 Appropriate probes were used for clarity and to elicit information. The information was debriefed 184 for participant validation after the interview, but the transcripts were not returned to them.

185 **analysis and statistics**

186 Quantitative

187 The data were analysed using EpiData Analysis (version 2.2.2.183, EpiData Association, Odense, 188 Denmark) and Stata (version 15.1, StataCorp LLC, College Station, Texas, USA). Patients not 189 undergoing a diagnostic test for TB within 30 days of referral were considered PDLFU. Patients 190 with diagnostic test after 30 days (n = 140) were excluded from the diagnostic evaluation 191 category. In case patients had diagnostic test performed prior to community referral, the time to 192 diagnostic visit was set as 0 days (n = 486). Age was missing for 242 community referrals. As

For peer review only - http://bmjopen.bmj.com/site/about/guidelines.xhtml

applicable, variables were summarized with mean (and standard deviation or SD) or median (and inter-quartile range or IQR) based on statistical distribution of data, or frequencies and percentages. Association of demographic and symptom characteristics with diagnostic evaluation was analyzed using χ2-square test and unadjusted relative risk (RR) with 95% confidence interval was calculated.

A multivariate analysis using Poisson's regression was used to calculate adjusted relative risk (aRR) with 95% confidence interval. Collinearity was assessed using variance inflation factors during model building. Likelihood ratio tests were used to identify factors that contributed significantly to the model. P-value ≤ 0.05 was considered statistically significant. A sensitivity analysis (univariate and multivariate) was performed by excluding patients whose diagnostic test was performed prior to screening (n = 486) and also by classifying patients who underwent diagnostic evaluation after 30 days as non-PDLFU (n = 140).

Qualitative

The transcripts were prepared on the same day using audio recording and field notes by TG. Manual descriptive content analysis was performed by two independent, trained researchers (TG, DS) to generate categories and themes.[23] Any discrepancies between the two were resolved through discussion. These were discussed and reviewed by RM to avoid subjective bias. The codes and themes were related back to the original data to ensure that the results reflect the data.[24]

patient and public involvement

Patients were neither involved in the study design nor in the interpretation of patient relevant outcomes. Nonetheless, patient's views were sought in the qualitative interviews and included in the results. The results of this study will be communicated to the patients and the public through a newsletter in the vernacular.

For peer review only

RESULTS

care cascade and characteristics of presumptive TB cases

We received a total of 13395 community referrals, out of which 90.9% (n=12180) were screened for symptoms. Of those screened, 91.5% (n=11146) were presumptive TB cases, and referred for diagnostic evaluation. (Figure 2)

There was nearly equal representation of presumptive TB cases from all the 3 blocks, and ASHAs identified most of them (75.6%). The mean age of presumptive TB cases was 35 years with majority in the 15 – 44 age group (41.8%). There were more males (52.2%) than females. (Table 1)

Table 1. Demographic profile of referrals, screened cases, and presumptive TB cases identified between January 2018 to December 2018 in Samastipur, India.

Characteristics	Community referrals, n(%)		Screened for TB, n(%)		Presumptive TB cases, n(%)	
**Total**	13395		12180		11146	
**Block**						
Ujiarpur	4275	(31.9%)	3926	(32.2%)	3620	(32.5%)
Bibhutipur	4609	(34.4%)	3994	(32.8%)	3673	(33%)
Sarairanjan	4511	(33.7%)	4260	(35.0%)	3853	(34.5%)
**Age**						
<15	2847	(21.3%)	2611	(21.4%)	2385	(21.4%)
15-44	5641	(42.1%)	5147	(42.3%)	4661	(41.8%)
45-64	3330	(24.9%)	3032	(24.9%)	2789	(25.0%)
≥65	1335	(10.0%)	1243	(10.2%)	1169	(10.5%)
Missing	242	(1.8%)	147	(1.2%)	142	(1.3%)
**Gender**						
Male	6973	(52.1%)	6391	(52.5%)	5827	(52.2%)
Female	6422	(47.9%)	5789	(47.5%)	5319	(47.7%)
**Source of referral**						
ASHA[1]	10091	(75.3%)	9210	(75.6%)	8428	(75.6%)
AWW[2]	105	(0.8%)	101	(0.8%)	88	(0.8%)
RMP[3]	724	(5.4%)	661	(5.4%)	617	(5.5%)
Community	2475	(18.5%)	2208	(18.1%)	2013	(18.1%)

[1]ASHA: Accredited Social Health Activist

[2]AWW: Anganwadi Worker

For peer review only

For peer review only - http://bmjopen.bmj.com/site/about/guidelines.xhtml

[3]RMP: Registered Medical Practitioner, an informal provider

The most common symptoms among presumptive TB cases were cough of ≥ 2 weeks (79.8%%), severe weight loss in the last 3 months (74.6%), fever of ≥ 2 weeks (73.9%), and chest pain in the last 1 month (63.4%), while hemoptysis in the last 6 months (12.1%) was the least common. Nearly one-fifth of the presumptive TB cases reported a previous history of anti-TB treatment (22.4%), and 20.5% were tobacco users. (Table 2)

Table 2: Characteristics of and risk factor for pre-diagnostic loss to follow up (PDLFU) among presumptive TB cases referred for diagnostic evaluation between January 2018 to December 2018 in Samastipur, India.

Characteristics	Presumptive TB cases, n	Not evaluated, n(%)	Unadjusted RR (95% CI)	p-value	Adjusted RR*(95% CI)	p-value
**Total**	11146	6234 (55.9%)				
**Block**						
Ujiarpur	3620	2060 (56.9%)	1.0 (1.0-1.1)	0.022	-	
Bibhutipur	3673	2083 (56.7%)	1.0 (1.0-1.1)	0.033	-	
Sarairanjan	3853	2091 (54.3%)	Ref		-	
**Age**						
<15	2385	1625 (68.1%)	1.4 (1.3-1.4)	0.000	1.2 (1.2-1.3)	0.000
15-44	4661	2459 (52.8%)	1.0 (1.0-1.1)	0.139	1.0 (1.0-1.1)	0.366
45-64	2789	1464 (52.5%)	1.0 (1.0-1.1)	0.212	1.0 (1.0-1.1)	0.164
≥65	1169	588 (50.3%)	Ref		Ref	
**Gender**						
Female	5827	3375 (57.9%)	Ref		Ref	
Male	5319	2859 (53.8%)	1.1 (1.0-1.1)	0.000	1.1 (1.0-1.1)	0.001
**Source of referral**						
ASHA[1]	8428	4690 (55.7%)	1.3 (1.2-1.4)	0.000	1.2 (1.1-1.3)	0.000
AWW[2]	88	52 (59.1%)	1.3 (1.1-1.6)	0.003	1.2 (1.0-1.5)	0.025
RMP[3]	617	271 (43.9%)	Ref		Ref	
Community	2013	1221 (60.7%)	1.4 (1.3-1.5)	0.000	1.3 (1.2-1.4)	0.000
**Previous history of anti-TB treatment**						
Yes	2501	1034 (41.3%)	0.7 (0.6-0.7)	0.000	0.7 (0.7-0.8)	0.000
No	8645	5200 (60.2%)	Ref			
**Presence of Signs and symptoms[#]**						
Haemoptysis in last 6 months	1346	494 (36.7%)	0.6 (0.6-0.7)	0.000	0.7 (0.6-0.7)	0.000
Cough ≥ 2 weeks	8895	4514 (50.8%)	0.7 (0.6-0.7)	0.000	-	

For peer review only

For peer review only - http://bmjopen.bmj.com/site/about/guidelines.xhtml

Sputum	6184	2779	(44.9%)	0.6	(0.6-0.7)	0.000	-
Chest pain in last 1 month	7061	3260	(46.2%)	0.6	(0.6-0.7)	0.000	-
Fever ≥ 2 weeks	8242	4289	(52.0%)	0.8	(0.8-0.8)	0.000	-
Night sweats ≥ 2 weeks	3730	1724	(46.2%)	0.8	(0.7-0.8)	0.000	-
Severe weight loss in last 3 months	8318	4254	(51.1%)	0.7	(0.7-0.8)	0.000	-
Swelling in a lymph node	2067	1517	(73.4%)	1.4	(1.4-1.5)	0.000	-
**Other factors[#]**							
Alcohol user	159	90	(56.6%)	1.0	(0.9-1.2)	0.862	-
Tobacco user	2280	1109	(48.6%)	0.8	(0.8-0.9)	0.000	-

[1]ASHA: Accredited Social Health Activist

[2]AWW: Anganwadi Worker

[3]RMP: Registered Medical Practitioner, an informal provider

Age is missing for 142 presumptive cases and 98 not evaluated cases.

All % are row percentages.

p-value ≤ 0.05 considered significant

[*] While building the model for multivariate Poisson's regression analysis, all the signs and symptoms except hemoptysis were dropped because of high collinearity assessed on the basis of high Variance Inflation Factor (VIF). The final variables in the model were selected on the basis of likelihood ratio testing.

[#] For signs, symptoms and other factors, the absence of that characteristic sign and symptoms was considered as the reference category.

characteristics of pre-diagnostic LFU and associated risk factors

Nearly 44% (n=4912) of the presumptive TB cases received diagnostic evaluation. The pre-diagnostic LFU was highest among younger (< 15 year) presumptive TB cases (68.1%), while lesser among those who were referred by an RMP (43.9%) and complained of hemoptysis in the last 6 months (36.7%). The median time to diagnosis was 1 day (IQR = 3).

On multivariate analysis, presumptive TB cases who were < 15 years of age (aRR = 1.2, 95% CI: 1.2-1.3, p = 0.00) were more likely to be PDLFU. Previous history of Tuberculosis treatment (aRR = 0.7, 95% CI: 0.7-0.8, p = 0.00) and hemoptysis in last 6 months (aRR = 0.7, 95% CI: 0.6-0.7, p = 0.00) decreased the chances of PDLFU. (Table 2)

In the sensitivity analysis, no change was observed in association of the risk factors with PDLFU on either excluding patients who were diagnosed prior to screening or classifying patients who underwent diagnostic evaluation after 30 days as non-PDLFU. (Supplementary table 1)

For peer review only - http://bmjopen.bmj.com/site/about/guidelines.xhtml

Enablers to access the first diagnostic evaluation

In the interviews, patients reported the following enablers for diagnostic evaluation: transport allowance for travel to the hospital, free services in the PHS and knowledge of the PHS procedures, accompaniment of ASHA and her assistance in the diagnostic process, and ASHA's understanding of the PHS functioning. (Figure 3)

> *"I started facing financial problems after going to the private hospital… Had I known the quality of care in the government hospital earlier, I would have come here. It helped that I didn't have to spend any money. I went by a vehicle to the PHC and consulted the doctor for free of cost."* (23 years old male patient)

> *"I didn't have to go to the hospital after submitting the sample for diagnostics… ASHA took me to the hospital and now sends the medicine… I was bony and couldn't even move a step earlier… I became better only from these government drugs; rest were useless. Between RMP and private clinic, we spent nearly □20,000* (~ USD 292).*"* (17 years old female TB patient on medication)

> *"When I had chest pain, ASHA told me that we'll have to go to the PHC, tests will be free, and I'll get return fare from my home. I thought I'll get better and decided to go. I got a blood test and x-ray done. Reports were normal and I got some medicines. I felt better… If you know the hospital and workers there, it is easier to get things done. Else, you have to spend a lot of time."* (30 years old female patient)

The ASHAs and the Field Coordinators (FCs) expressed transport allowance, incentive to ASHA, positive patient experience, and patient accompaniment as enablers. (Figure 3)

> *"If one patient becomes better, she also advises others. Even if we don't know, the patient who becomes better asks their ill neighbor to call the ASHA for everything. Our mobile number is available in every household."* (ASHA)

> *"Sometimes, patients do not have any shared conveyance from their home. In such cases, patients are unable to come, and transport allowance or hiring an autorickshaw for, say 10 patients of the village, really helps. That way, they get consultation and lab tests at*

For peer review only

For peer review only - http://bmjopen.bmj.com/site/about/guidelines.xhtml

PHC, then chest x-ray at another diagnostic facility, and get dropped at their home.” (Field coordinator)

“ASHA’s income is from incentives, and if she gets incentives in time, she takes up those activities.” (Field coordinator)

barriers to access the first diagnostic evaluation

Barriers were coded from the interview and FGD transcripts into 5 categories and 27 codes. These barriers are listed in the Figure 3 and described below.

Category 1: Logistics-related

As per patients, public transport was not available in certain locations and some patients mentioned their inability to pay for it even where it was available. They also reported not knowing the location of the diagnostic center.

“I don’t know where the diagnostic center is. From my village, I have to walk for 3 – 4 km before I find any transport.” (65 years old woman)

The ASHA and FC corroborated that transport and insufficient support for certain patient population like disabled people was a key logistics-related challenge.

“Travel options are also limited for some areas. Either it is not readily available or you have to walk before anything can be found. They don’t have the money to reserve an entire shared vehicle. If the patient is disabled or elderly, they face even more problems.” (Field coordinator)

Category 2: Health system-related

According to patients, the care at PHC was slow and took a lot of time. They said either the PHC remained closed or they didn’t reach the PHC in working hours. At other times when they reached, the providers were not available at the facility.

“I had to go 2-3 times to the PHC before all the tests were completed and reports arrived. Only then I could get the medicines.” (17 years old female patient)

For peer review only

For peer review only - http://bmjopen.bmj.com/site/about/guidelines.xhtml

"We have to run-around a lot in the government hospital. There are huge buildings and it is difficult to figure out what happens where alone." (30 years old male patient)

The health workers reported unavailability of some diagnostic services in the PHC and the high cost of getting these in the private sector. ASHA's remuneration for providing TB care services was irregular and unpaid. They indicated that poor patient experience at the PHC also deterred others and that the public put a stronger faith in the private sector.

"Patient doesn't know where the registration desk is, where the doctor sits, where the labs and x-ray are done. We have to go with them to get everything completed quickly." (ASHA)

"I have provided treatment to 20 patients, but I haven't been paid for it. It is only in the recent 1-2 years that there has been some improvement. We are not told properly how to fill the document and what to submit for release of payments. All our papers were taken and nothing happened. Earlier we used to only counsel the patient to go for diagnostics, but now we come with them if we get money for helping in diagnosis." (ASHA)

"If patient come on a given day and their work doesn't get done, they won't come again. Coming again will mean foregoing another day's wage. They will say that ASHA cheated them and nothing happened at the hospital and nothing is available at the PHC ever. If they go to a private clinic, everything is done in one day and they get the medicine… They also prefer private." (ASHA)

Category 3: Health provider-related

Patients described health worker strikes in the field and at the PHC as a barrier. One patient also reported that ASHA doesn't come to their home.

"I took the sputum to the PHC, but nothing was done. PHC was on strike for 15 days. The unabated cough and fever didn't let me sleep. When I felt that I would die, I took an injection from the RMP and drank 5 cough syrups. Only then was there any relief." (65 years old male patient)

For peer review only - http://bmjopen.bmj.com/site/about/guidelines.xhtml

"What will happen after going to the PHC? I go to the hospital and come back with nothing. Even ASHA doesn't come. I am going to lose everything in this hustle and bustle." (35 years old female patient)

The health workers reported RMPs misguiding the patients, low morale of the workers, and poor health provider behavior at the PHC as barriers.

"RMPs misguide the patients a lot. They give medications and injections without any concern. They are not at risk even if the patient dies. If patient becomes serious and they have made money, they will refer them to hospital. They roam through the villages and people get medicine at their doorstep. Someone who is a laborer, their income for the day is saved." (ASHA)

"When we take the patient to the PHC, lab technician will return the sample and asks to come on another day... Doctor will also not write any medication and don't know the updated guidelines… We have to then listen from our patient." (ASHA)

Category 4: Family-related

Patients indicated not knowing the extent of available services in the PHS. Some described severe indebtedness of the family and not having any money for healthcare. According to them, insufficient family support and absence of a caretaker in the family were also barriers.

"I live with my two granddaughters and my son works as a watchman in New Delhi. The money is irregular. If I've no money, I won't go. In November, I didn't have money and couldn't go… For consulting with the RMP, I had to take a loan of □150 (~ USD 2.2). *After a week, I've been able to return only □50."* (65 years old female patient)

"Both my kids are alone. My husband has migrated for work. He says that take the kids to a senior doctor in a private hospital. It'll cost at least □3000 (~ USD 44) *and I am waiting for it… There is only my old mother-in-law in the house besides me. How will I leave them alone at home?"* (Mother of a 6 years old patient)

For peer review only

Health workers mentioned permission from the head of the family to visit facility, restricted mobility of women in the families as compared to men, and family's concern only if symptoms were severe as barriers.

> *"Patients say that we'll ask the head of the family and come only if they assent. Sometimes they are not sure of the quality of care in the PHS. We also counsel the guardian… This happens more often for women. Men are independent; they don't have to ask the head or the wife… It is also difficult for women. They have to complete household chores and care for the kids before leaving home for the facility. Often, by the time they reach, the facility will close."* (ASHA)

> *"If the symptoms are not severe, then patient takes some medicine from RMP at their home and doesn't want to come for a test."* (Field coordinator)

Category 5: Patient-related

Patients indicated denial of suffering from TB.

> *"I don't need any diagnostics. I didn't have TB ever in my family. This is all because of gas."* (65 years old patient)

As per health workers, some patients had a fear of TB. They also reported discrimination by the community against TB patients.

> *"We don't say TB at first. If we say it, people hesitate. Some are offended that we have said TB is a possible disease for them. We've to tell them gently. One sputum positive patient said that she was hurt that I said she has TB."* (ASHA)

> *"A lot of people prefer to keep their disease status hidden. TB is considered contagious and people don't want neighbors to know of it. Earlier, even patient's utensils and bedding were kept separate."* (ASHA)

DISCUSSION

This is the first such study to estimate the PDLFU in an ACF program while also investigating the barriers and enablers in accessing diagnostic evaluation. About 44% of the presumptive TB cases

For peer review only - http://bmjopen.bmj.com/site/about/guidelines.xhtml

couldn’t undergo diagnostic evaluation. Provision of transport allowance for patients, accompaniment and support of ASHA, incentives to ASHA, and knowledge of the PHS procedures emerged as key enablers. The major barriers were misinformation and stigma, insufficient family and health provider support, transport despite an allowance, inadequate health services, and poor faith in the public sector.

The strengths of the study are as follows. First, we used a mixed-methods design in which the quantitative and qualitative components complement each other. The qualitative component explored hitherto unresearched reasons for the PDLFU and recorded the perspectives of both patients and CHWs. Second, the study was conducted in an ACF program using routinely collected data, hence, reflecting the field reality.

There are several limitations as well. First, the ACF intervention was an addon to the RNTCP program with extra provisions like travel allowance. Since the project removed certain barriers in undergoing diagnostic evaluation, the analysis is not necessarily generalizable to routine care. Second, the CHWs and RMPs were likely filtering patients before they were referred to the program. Therefore, the true number of people screened would be higher than that reported in the study. Third, while the presumptive TB cases receiving a test was known, there was no information on cases reaching the facility but not receiving a test or people not completing the diagnostic process.

While 96.6% of the presumptive TB cases received a CXR in the mobile van in the ACF project in Myanmar, only 51.4% of the cases referred for sputum microscopy to the health facility followed through.[12] Similarly, in a ACF campaign in India, only 22% referred people made it to the DMC, but about 54% received sputum microscopy after provision of specimen transport.[13] In comparison, 41.1% of the presumptive TB cases in our study underwent diagnostic evaluation, which included 81.6% and 50.6% receiving a CXR and sputum microscopy respectively at a health facility. Referral with adequate support like that through ASHA accompaniment and community-oriented TB service delivery like using mobile vans can complement each other in diagnostic evaluation. In the case of mobile CXR, immediate availability of a diagnostic test for which people didn’t have to travel encouraged uptake. Furthermore, specimen transport helped in microbiological testing by moving the sample instead of the patient, but challenges like

For peer review only

For peer review only - http://bmjopen.bmj.com/site/about/guidelines.xhtml

refrigeration and sample preparation are aplenty.[25] Point-of-care diagnostic tests like the upcoming GeneXpert Omni and Truenat can improve uptake of microbiological testing in such scenarios.[26,27] The Zimbabwe study reported place of residence (rural) and type of facility (private) as important risk factor for pre-diagnostic LFU.[28] While we found association of PDLFU with various risk factors like age and previous history of TB, they are of limited public health or clinical importance.

Nonetheless, qualitative findings provide rich insights. The transport allowance, free diagnostic services, ASHA's accompaniment supported the patients. While covering the financial costs of the patient certainly helps, opportunity cost like lost wages also need to be considered.[29,30] In fact, barriers like indebtedness of family can exacerbate the opportunity cost, consequently, restricting the benefits of covering financial cost through transport allowance and free diagnostics.[31] Furthermore, systemic factors like unavailability of transport options limits the effectiveness of such solutions.[32,33] The health system and health provider issues like cumbersome processes in health facilities and poor provider behavior resonated in our findings.[34] These patient narratives add explanation to people's preference for private sector and informal providers even though affordable services are available in the PHS. People's trust in the provider is a crucial factor in determining their preferred provider, which was also corroborated by patients in our study.[35] The disparity is further accentuated by variability of ASHAs' quality.[36] In our study, a patient complained of their ASHA's lackadaisical attitude and another failed to find the diagnostic center indicating that their ASHA didn't help. This difference between ASHAs was also confirmed by the field coordinators: not all ASHAs are equally interested or equally capable. This will need attention in programs involving ASHA to encourage their active engagement.

From patients' perspective, stigma, misinformation, and insufficient family support emerged as important barriers. The support to patients is often dependent on their family and its situation. For instance, women whose husband migrated for work have to manage the household with limited finances and find it difficult to make time during the visiting hours of labs. Additional challenges of a patriarchal society like restricted mobility and family elder's permission compound the problem for women.[37] Similarly, older and disabled people may not even have

For peer review only

the essential family or systemic support. Furthermore, the misinformation and stigma in the community against TB force the patients to keep the diagnosis to themselves. Although the ASHAs reported that stigmatizing practices have improved, a thorough investigation needs to be undertaken to enable to further reduce the negative influence of stigma on TB care and treatment..

We suggest a two-pronged approach to improve the situation: a community-level effort focusing on stigma and patient support, and a systemic reform in the health system. A sustained campaign on awareness of the disease along with services available for such patients is a must. As the campaign corrects misinformation, it creates a viable climate to tackle the stigma. Delivering such a campaign effectively will need involvement of all sections of the community, in particular the opinion shapers and influencers in the region.[38] The campaigns will need contextualization based on the population's language and culture. Both informal and formal community association, like religious groups or self-help groups, can be a possible vehicle.[39] In fact, such groups along with patient support network can be leveraged to create a support system for patients too.[40] The second prong hinges on building a system of seamless care and improving quality in the public sector.[41,42] Private sector interventions in TB care have demonstrated seamless care through processes like social franchising for diagnostic tests.[43] Similarly, trusted informal providers deliver services at the patient's doorstep at a convenient time, thus, also reducing the opportunity costs. PHS needs to learn from the private and informal providers to be a competitive source of healthcare services.[44–46] It will require investment in taking healthcare closer to the community by steps like upgrading the CHWs, adequate supportive supervision, and instituting health outposts.[47] In addition, quality needs to be a core principle in the health system , both private and public. Involving people in assessing the local health system by social audits and patient feedback, and embedding quality in the performance indictor of the health system and health workers can be a starting step.

In conclusion, these findings are relevant to the broader primary health services in addition to the TB program. While improving care in the public health system is essential to make it competitive, involving the community and considering sociocultural factors will be critical to its success.

For peer review only - http://bmjopen.bmj.com/site/about/guidelines.xhtml

ACKNOWLEDGEMENT

We are grateful for the support of RNTCP, Government of Bihar, State TB Officer Dr. (Major) K. N. Sahai, Communicable Disease Officer Dr. Sree Ram Prasad, and District Health Society, Samastipur. We thank Sriram Selvaraju, Anthony D. Harries, Dick Menzies, and participants of the TB Research Methods course at McGill Summer Institute 2018 for their comments on the protocol, and Amol Dongre for his feedback on the qualitative results. The program couldn't have been implemented without the tireless efforts of ASHAs, health providers, and the team at Innovators In Health.

This research was conducted through the Structured Operational Research and Training Initiative (SORT IT), a global partnership led by the Special Programme for Research and Training in Tropical Diseases at the World Health Organization (WHO/TDR). The model is based on a course developed jointly by the International Union Against Tuberculosis and Lung Disease (The Union) and Medécins sans Frontières (MSF/Doctors Without Borders). The specific SORT IT programme which resulted in this publication was jointly developed and implemented by: The Union South-East Asia Office, New Delhi, India; the Centre for Operational Research, The Union, Paris, France; Department of Preventive and Social Medicine, Jawaharlal Institute of Postgraduate Medical Education and Research, Puducherry, India; Department of Community Medicine and School of Public Health, Postgraduate Institute of Medical Education and Research, Chandigarh, India; Department of Community Medicine, All India Institute of Medical Sciences, Nagpur, India; Dr. Rajendra Prasad Centre for Ophthalmic Sciences, All India Institute of Medical Sciences, New Delhi, India; Department of Community Medicine, Pondicherry Institute of Medical Science, Puducherry, India; Department of Community Medicine, Kalpana Chawla Medical College, Karnal, India; National Centre of Excellence and Advance Research on Anemia Control, All India Institute of Medical Sciences, New Delhi, India; Department of Community Medicine, Sri Manakula Vinayagar Medical College and Hospital, Puducherry, India; Department of Community Medicine, Velammal Medical College Hospital and Research Institute, Madurai, India; Department of Community Medicine, Yenepoya Medical College, Mangalore, India; Karuna Trust, Bangalore, India and National Institute for Research in Tuberculosis, Chennai, India.

For peer review only - http://bmjopen.bmj.com/site/about/guidelines.xhtml

COMPETING INTERESTS

None declared

FUNDING

This project was supported by the Stop TB Partnership's TB REACH initiative and was funded by the Government of Canada and the Bill & Melinda Gates Foundation. Manish Bhardwaj and Dyuti Sen are supported by a Grand Challenges Explorations grant number OPP1190735 and OPP1190905 respectively from the Bill & Melinda Gates Foundation. The training program, within which this paper was developed, was funded by the Department for International Development (DFID), UK. No funders had any role in study design, data collection and analysis, decision to publish, or preparation of the manuscript.

For peer review only

DATA AVAILABILITY

The deidentified data used in the study is available from the corresponding author on reasonable request. It contains information on patient demographics and characteristics, screening criteria, diagnostic test used, and final diagnosis. There is no additional data available beyond that.

ETHICS APPROVAL

Ethical approval was obtained from Institutional Ethics Committee, Emmanuel Hospital Association, New Delhi (Order 201) and the Ethics Advisory Group, International Union Against Tuberculosis and Lung Disease, Paris (EAG number 98/18). The committee waived the informed consent requirement for quantitative study. Informed consent was obtained from participants before the in-depth interviews and FGDs.

AUTHOR CONTRIBUTION

Conceptualization of intervention: TG, DS, MaB

Study design: TG, VG, DS, MV, MiB, RM, MaB

Data collection: TG, DS, RM

Data analysis and interpretation: TG, VG, DS, MV, RM, MaB

For peer review only - http://bmjopen.bmj.com/site/about/guidelines.xhtml

Writing — original draft: TG

Writing — review and edit: TG, VG, DS, MV, MiB, RM, MaB

REFERENCES

1 World Health Organization. Gear up to end TB: introducing the end TB strategy. World Health Organization 2015. https://apps.who.int/iris/handle/10665/156394?show=full (accessed 18 Nov 2019).

2 Reid MJA, Arinaminpathy N, Bloom A, *et al.* Building a tuberculosis-free world: The Lancet Commission on tuberculosis. *The Lancet* 2019;**393**:1331–84. doi:10/gfxc87

3 World Health Organization. *Global tuberculosis report 2019*. World Health Organization 2019. https://apps.who.int/iris/handle/10665/329368 (accessed 18 Oct 2019).

4 Lönnroth K, Corbett E, Golub J, *et al.* Systematic screening for active tuberculosis: rationale, definitions and key considerations [State of the art series. Active case finding/screening. Number 1 in the series]. *The International Journal of Tuberculosis and Lung Disease* 2013;**17**:289–98. doi:10/gd52dz

5 Storla DG, Yimer S, Bjune GA. A systematic review of delay in the diagnosis and treatment of tuberculosis. *BMC Public Health* 2008;**8**:15. doi:10/c6sz7r

6 Subbaraman R, Nathavitharana RR, Satyanarayana S, *et al.* The Tuberculosis Cascade of Care in India's Public Sector: A Systematic Review and Meta-analysis. *PLOS Medicine* 2016;**13**:e1002149. doi:10/f9b29f

7 Wells WA. Onions and prevalence surveys: how to analyze and quantify tuberculosis case-finding gaps. *The International Journal of Tuberculosis and Lung Disease* 2017;**21**:1101–13. doi:10/gb4g9c

8 Ho J, Fox GJ, Marais BJ. Passive case finding for tuberculosis is not enough. *International Journal of Mycobacteriology* 2016;**5**:374–8. doi:10/gcpfsf

For peer review only - http://bmjopen.bmj.com/site/about/guidelines.xhtml

9 Creswell J, Sahu S, Blok L, *et al.* A Multi-Site Evaluation of Innovative Approaches to Increase Tuberculosis Case Notification: Summary Results. *PLOS ONE* 2014;**9**:e94465. doi:10/gcz84n

10 Mhimbira FA, Cuevas, LE, Dacombe, R, Mkopi, A, Sinclair D. Interventions to increase tuberculosis case detection at primary healthcare or community-level services. *Cochrane Database of Systematic Reviews* Published Online First: 2017. doi:10/gcmjrv

11 Adejumo AO, Azuogu B, Okorie O, *et al.* Community referral for presumptive TB in Nigeria: a comparison of four models of active case finding. *BMC Public Health* 2016;**16**:177. doi:10/f8bjrx

12 Myint O, Saw S, Isaakidis P, *et al.* Active case-finding for tuberculosis by mobile teams in Myanmar: yield and treatment outcomes. *Infectious Diseases of Poverty* 2017;**6**:77. doi:10/gf38nz

13 Prasad BM, Satyanarayana S, Chadha SS, *et al.* Experience of active tuberculosis case finding in nearly 5 million households in India. *Public Health Action* 2016;**6**:15–8. doi:10/gd4v8q

14 Creswell JW, Clark VLP. *Designing and Conducting Mixed Methods Research*. SAGE 2011.

15 Registrar General of India. Census of India 2011. Office of the Registrar General Census Commissioner, India 2011.

16 Annual Health Survey 2011-12, Bihar Factsheet. New Delhi: : Office of the Registrar General & Census Commissioner http://www.censusindia.gov.in/vital_statistics/AHSBulletins/AHS_Factsheets_2011_12/Bihar_Factsheet_2011-12.pdf (accessed 4 Feb 2019).

17 Central TB Division. India TB Report 2018. New Delhi, India: : Directorate General of Health Services, Ministry of Health and Family Welfare, Government of India 2018. https://tbcindia.gov.in/showfile.php?lid=3314 (accessed 18 Nov 2019).

For peer review only - http://bmjopen.bmj.com/site/about/guidelines.xhtml

18 Uplekar M, Pathania V, Raviglione M. Private practitioners and public health: weak links in tuberculosis control. *The Lancet* 2001;**358**:912–6. doi:10/c8jf9s

19 National Informatics Centre, Patna. Health Department Geographical Information System, Bihar. http://gis.bih.nic.in/Depts/Health.aspx (accessed 14 Sep 2018).

20 Update on ASHA Programme. New Delhi: : National Health Mission, Ministy of Health and Family Welfare 2018. http://nhsrcindia.org/sites/default/files/asha%20reduced.pdf (accessed 29 Jul 2019).

21 Revised Technical & Operational Guidelines for Tuberculosis Control in India. New Delhi: : Central TB Division 2016. https://www.tbcindia.gov.in/WriteReadData/Revised%20Technical%20and%20Operational%20Guidelines/files/assets/basic-html/page-1.html# (accessed 4 Feb 2019).

22 Integrated Child Development Services Scheme. Ministry of Women and Child Development, Government of India. https://icds-wcd.nic.in/icds.aspx (accessed 17 Aug 2019).

23 Attride-Stirling J. Thematic networks: an analytic tool for qualitative research. *Qualitative research* 2001;**1**:385–405. doi:10/cg4grf

24 Lincoln Y, Guba E. Naturalistic Inquiry: Designing a naturalistic inquiry. *Beverly Hills: Sage Publications* 1985.

25 Mundy C. J. F, Harries A. D, Banerjee A, *et al.* Quality assessment of sputum transportation, smear preparation and AFB microscopy in a rural district in Malawi. *The International Journal of Tuberculosis and Lung Disease* 2002;**6**:47–54.

26 Drain PK, Garrett NJ. The arrival of a true point-of-care molecular assay—ready for global implementation? *The Lancet Global Health* 2015;**3**:e663–4. doi:10/ggdfph

For peer review only

For peer review only - http://bmjopen.bmj.com/site/about/guidelines.xhtml

27 Lee DJ, Kumarasamy N, Resch SC, *et al.* Rapid, point-of-care diagnosis of tuberculosis with novel Truenat assay: Cost-effectiveness analysis for India's public sector. *PLOS ONE* 2019;**14**:e0218890. doi:10/ggdfpj

28 Padingani M, Kumar A, Tripathy JP, *et al.* Does pre-diagnostic loss to follow-up among presumptive TB patients differ by type of health facility? An operational research from Hwange, Zimbabwe in 2017. *Pan African Medical Journal* 2018;**31**. doi:10.11604/pamj.2018.31.196.15848

29 Sarin R, Vohra V, Singla N, *et al.* Identifying costs contributing to catastrophic expenditure among TB patients registered under RNTCP in Delhi metro city in India. *Indian Journal of Tuberculosis* 2019;**66**:150–7. doi:10/ggdfpz

30 Tanimura T, Jaramillo E, Weil D, *et al.* Financial burden for tuberculosis patients in low- and middle-income countries: a systematic review. *European Respiratory Journal* 2014;**43**:1763–75. doi:10/f56btb

31 Sangar S, Dutt V, Thakur R. Economic burden, impoverishment and coping mechanisms associated with out-of-pocket health expenditure: analysis of rural-urban differentials in India. *J Public Health (Berl)* 2018;**26**:485–94. doi:10/ggdfpx

32 Tripathy JP, Srinath S, Naidoo P, *et al.* Is physical access an impediment to tuberculosis diagnosis and treatment? A study from a rural district in North India. *Public Health Action* 2013;**3**:235–9. doi:10/ggdfp8

33 Kasthuri A. Challenges to Healthcare in India - The Five A's. *Indian J Community Med* 2018;**43**:141–3.

34 Bhatnagar H. User-experience and patient satisfaction with quality of tuberculosis care in India: A mixed-methods literature review. *Journal of Clinical Tuberculosis and Other Mycobacterial Diseases* 2019;**17**:100127. doi:10/ggdfn3

For peer review only

For peer review only - http://bmjopen.bmj.com/site/about/guidelines.xhtml

612 35 Anand G, Chhajed D, Shah S, *et al.* Do qualifications matter? A qualitative study of how villagers decide their health care providers in a developing economy. *PLOS ONE* 2019;**14**:e0220316. doi:10/ggdfqr

615 36 Kumar S, Kaushik A, Kansal S. Factors Influencing the Work Performance of ASHA Under NRHM: A Cross Sectional Study from Eastern Uttar Pradesh. *Indian Journal of Community Health* 2012;**24**:325–31.

618 37 Roy K, Chaudhuri A. Influence of socioeconomic status, wealth and financial empowerment on gender differences in health and healthcare utilization in later life: evidence from India. *Social Science & Medicine* 2008;**66**:1951–62. doi:10/dhww4j

621 38 Heijnders M, Meij SVD. The fight against stigma: An overview of stigma-reduction strategies and interventions. *Psychology, Health & Medicine* 2006;**11**:353–63. doi:10/ddr4bb

623 39 Brody C, Hoop T de, Vojtkova M, *et al.* Economic Self-Help group Programs for Improving Women's Empowerment: A Systematic Review. *Campbell Systematic Reviews* 2015;**11**:1–182. doi:10/ggdfqv

626 40 Brody C, Hoop T de, Vojtkova M, *et al.* Can self-help group programs improve women's empowerment? A systematic review. *Journal of Development Effectiveness* 2017;**9**:15–40. doi:10/gf44gk

629 41 Balarajan Y, Selvaraj S, Subramanian S. Health care and equity in India. *The Lancet* 2011;**377**:505–15. doi:10/crkb78

631 42 Agins BD, Ikeda DJ, Reid MJA, *et al.* Improving the cascade of global tuberculosis care: moving from the "what" to the "how" of quality improvement. *The Lancet Infectious Diseases* doi:10/ggdfq6

634 43 Wells WA, Uplekar M, Pai M. Achieving Systemic and Scalable Private Sector Engagement in Tuberculosis Care and Prevention in Asia. *PLOS Medicine* 2015;**12**:e1001842. doi:10/ggdfq8

For peer review only - http://bmjopen.bmj.com/site/about/guidelines.xhtml

44 Gautham M, Shyamprasad KM, Singh R, *et al.* Informal rural healthcare providers in North and South India. *Health Policy Plan* 2014;**29**:i20–9. doi:10/f6cp72

45 Sudhinaraset M, Ingram M, Lofthouse HK, *et al.* What Is the Role of Informal Healthcare Providers in Developing Countries? A Systematic Review. *PLOS ONE* 2013;**8**:e54978. doi:10/ggdfq3

46 Ergler CR, Sakdapolrak P, Bohle H-G, *et al.* Entitlements to health care: Why is there a preference for private facilities among poorer residents of Chennai, India? *Social Science & Medicine* 2011;**72**:327–37. doi:10/bqs6px

47 Sinha P, Shenoi SV, Friedland GH. Opportunities for community health workers to contribute to global efforts to end tuberculosis. *Global Public Health* 2019;**0**:1–11. doi:10/ggdfqx

For peer review only

LEGEND FOR FIGURES

Figure 1: Operational definitions used in the program

CHW: Community Health Worker

ASHA: Accredited Social Health Activist

AWW: Anganwadi Worker

CXR: Chest X-ray

USG: Ultrasonography

Figure 2: TB care cascade between January 2018 and December 2018 from community referral to diagnostic evaluation in a community-based ACF program in Samastipur, India

All percentages are calculated as a proportion of the number of participants entering the previous step of the cascade.

* Includes 140 Presumptive TB cases undergoing diagnostic evaluation beyond 30 days from screening.

For peer review only - http://bmjopen.bmj.com/site/about/guidelines.xhtml

Figure 3: The enablers and barriers in diagnostic evaluation from patients', ashas', and field coordinators' perspective in an ACF TB program in Samastipur, India from January 2018 to December 2018

[a]: responses of patient

[b]: responses of ASHA

[c]: responses of FC

ASHA: Accredited Social Health Activist

PHS: Public Health System

PHC: Primary Health Centre

For peer review only

For peer review only - http://bmjopen.bmj.com/site/about/guidelines.xhtml

Term	Definition
Community referral	A person at risk of TB referred for TB screening to the program by a CHW (ASHA or AWW) or an informal provider or community during their routine work
Presumptive TB patient	A person identified with one or more of the following symptoms of TB in the screening process at their home: cough ≥ 2 weeks; sputum in cough, hemoptysis in last 6 months; chest pain in last 1 month; fever ≥ 2 weeks; night sweats ≥ 2 weeks; severe weight loss in last 3 months; swelling in a lymph node
Diagnostic evaluation	A presumptive TB patient tested using one or more of a sputum microscopy or CXR or GeneXpert or extrapulmonary TB testing (USG or FNAC) within 30 days of screening
Pre-diagnostic loss to follow-up	A presumptive TB patient not undergoing a diagnostic evaluation

For peer review only - http://bmjopen.bmj.com/site/about/guidelines.xhtml

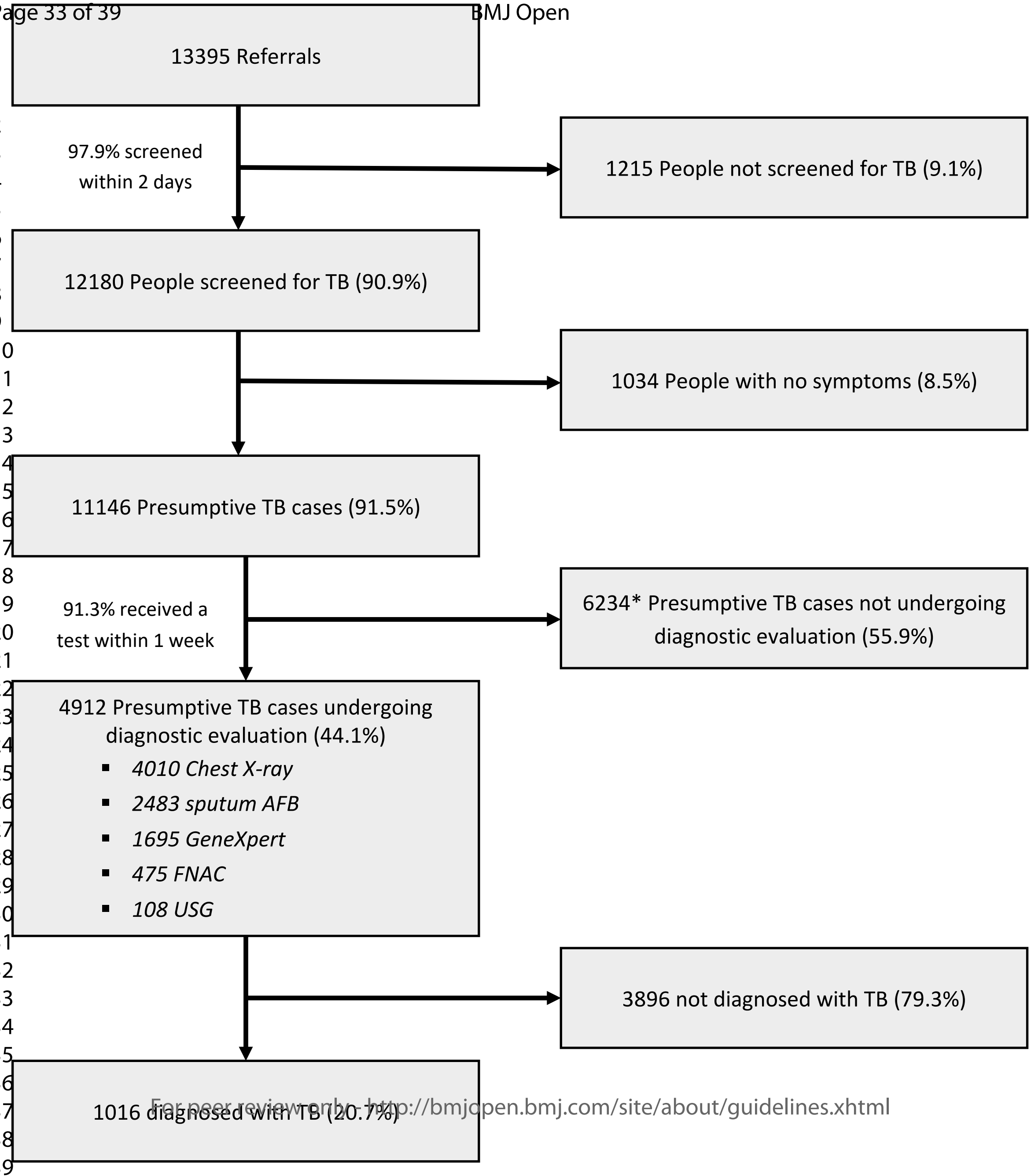

For peer review only - http://bmjopen.bmj.com/site/about/guidelines.xhtml

Enablers

Category 1: Logistics-related

- Transport allowance [a,b,c]

Category 2: Health system-related

- Free services in the PHS [a,b]
- Knowledge of the PHS procedures [a]
- Incentive to ASHA for TB work is vital [b,c]
- Previous positive patient experience in PHS [b,c]

Category 3: Health provider-related

- ASHA's assistance in the diagnostic process [a]
- ASHA's accompaniment to the health facility [a,c]
- ASHA's understanding of the PHS functioning [a]
- Satisfied people referred others to ASHA [b]

Barriers

Category 1: Logistics-related

- Unavailability of public transport from patient's village [a,c]
- No knowledge of the diagnostic center location [a]
- No transport support for the disabled patient [b]

Category 2: Health system-related

- Care at PHC was slow and time-taking [a,b,c]
- Not all diagnostics were available in the PHC [b]
- High cost of services not available in the PHC [b]
- Difficult to understand PHC system [a,b]
- PHC remained closed [a]
- Inability to reach PHC in working hours [a,b]
- Provider was unavailable at the PHC [a]
- Irregular and unpaid ASHA incentives [b]
- Faith in the private sector [b,c]
- Poor patient experience deterred others as well [b]

Category 3: Health provider-related

- Informal providers misguided the patient [b,c]
- Low morale of the health providers [b]
- Health worker strikes [a]
- Poor health provider behavior at PHC [b,c]
- ASHA didn't come to the patient's home [a]

Category 4: Family-related

- Poor awareness of TB care in PHS [a,c]
- Family concerned only when symptoms were severe [b]
- Insufficient family support [a]
- No caretaker in the family to help in reaching hospital [a]
- Family permission was necessary [b]
- Indebted, and no money for healthcare [a]
- Restricted mobility of women [b]

Category 5: Patient-related

- Denial of suffering from TB [a]
- Fear of TB [b]
- Discrimination against diseased patient [b,c]

For peer review only - http://bmjopen.bmj.com/site/about/guidelines.xhtml

SUPPLEMENTARY TABLE 1

Sensitivity analysis on including patient with negative diagnostic delay as zero day delay, after excluding patients with diagnostic evaluation before screening visit, and classifying patients with over 30 days delay in diagnostic evaluation as non-PDLFU

	Including patients with negative diagnostic delay as 0 days diagnostic delay[$]						Excluding patients with negative diagnostic delay[$]						Including patients with diagnostic delay of over 30 days as non-PDLFU					
Characteristics	Univariate analysis			Multivariate analysis			Univariate analysis			Multivariate analysis			Univariate analysis			Multivariate analysis		
	RR	95%CI	p-Value	aRR^	95%CI	p-Value	RR	95%CI	p-Value	aRR^	95%CI	p-Value	RR	95%CI	p-Value	aRR^	95%CI	p-Value
**Block**																		
Ujiarpur	1.0	(1.0-1.1)	0.022	-			1.1	(1.0-1.1)	0.004	-			1.0	(1.0-1.1)	0.04	-		
Bibhutipur	1.0	(1.0-1.1)	0.033	-			1.0	(1.0-1.1)	0.033	-			1.0	(1.0-1.1)	0.08	-		
Sarairanjan		Ref		-				Ref		-				Ref		-		
**Age**																		
<15	1.4	(1.3-1.4)	0.000	1.2	(1.2-1.3)	0.000	1.3	(1.3-1.4)	0.000	1.3	(1.2-1.3)	0.000	1.3	(1.2-1.4)	0.000	1.2	(1.1-1.3)	0.000
15-44	1.0	(1.0-1.1)	0.139	1.0	(1.0-1.1)	0.366	1.1	(1.0-1.1)	0.06	1.0	(1.0-1.1)	0.180	1.0	(1.0-1.1)	0.216	1.0	(1.0-1.1)	0.539
45-64	1.0	(1.0-1.1)	0.212	1.0	(1.0-1.1)	0.164	1.1	(1.0-1.1)	0.143	1.1	(1.0-1.1)	0.099	1.0	(1.0-1.1)	0.295	1.0	(1.0-1.1)	0.242
≥65		Ref		Ref				Ref			Ref			Ref			Ref	
**Gender**																		
Female		Ref			Ref			Ref			Ref			Ref			Ref	
Male	1.1	(1.0-1.1)	0.000	1.1	(1.0-1.1)	0.001	1.1	(1.0-1.1)	0.000	1.1	(1.0-1.1)	0.003	1.1	(1.0-1.1)	0.000	1.1	(1.0-1.1)	0.000
**Source of referral**																		
RMP[3]		Ref			Ref			Ref			Ref			Ref			Ref	
ASHA[1]	1.3	(1.2-1.4)	0.000	1.2	(1.1-1.3)	0.000	1.3	(1.2-1.4)	0.000	1.2	(1.1-1.3)	0.000	1.3	(1.2-1.4)	0.000	1.2	(1.1-1.4)	0.000
AWW[2]	1.3	(1.1-1.6)	0.003	1.2	(1.0-1.5)	0.025	1.3	(1.1-1.6)	0.005	1.2	(1.1-1.5)	0.030	1.4	(1.1-1.7)	0.000	1.3	(1.1-1.6)	0.012
Community	1.4	(1.3-1.5)	0.000	1.3	(1.2-1.4)	0.000	1.4	(1.3-1.5)	0.000	1.3	(1.2-1.4)	0.000	1.4	(1.3-1.6)	0.000	1.2	(1.2-1.5)	0.000
**Signs and symptoms**																		
Hemoptysis in last 6 months	0.6	(0.6-0.7)	0.000	0.7	(0.6-0.7)	0.000	0.6	(0.6-0.7)	0.000	0.8	(0.7-0.8)	0.000	0.6	(0.6-0.7)	0.000	0.7	(0.6-0.7)	0.000
Cough ≥ 2 weeks	0.7	(0.6-0.7)	0.000	-			0.7	(0.7-0.7)	0.000	-			0.7	(0.6-0.7)	0.000	-		
Sputum	0.6	(0.6-0.7)	0.000	-			**0.7**	**(0.6-0.7)**	0.000	-			0.6	(0.6-0.7)	0.000	-		
Chest pain in last 1 month	0.6	(0.6-0.7)	0.000	-			**0.7**	**(0.6-0.7)**	0.000	-			0.6	(0.6-0.7)	0.000	-		
Fever ≥ 2 weeks	0.8	(0.8-0.8)	0.000	-			0.8	(0.8-0.8)	0.000	-			0.8	(0.7-0.8)	0.000	-		
Night sweats ≥ 2 weeks	0.8	(0.7-0.8)	0.000	-			0.8	(0.7-0.8)	0.000	-			0.8	(0.7-0.8)	0.000	-		
Severe weight loss in last 3 months	0.7	(0.7-0.8)	0.000	-			0.7	(0.7-0.8)	0.000	-			0.7	(0.7-0.8)	0.000	-		
Swelling in a lymph node	1.4	(1.4-1.5)	0.000	-			1.4	(1.3-1.4)	0.000	-			1.4	(1.3-1.4)	0.000	-		

For peer review only - http://bmjopen.bmj.com/site/about/guidelines.xhtml

**Other factors**																-		
Previous history of anti-TB treatment	0.7	(0.6-0.7)	0.000	0.7	(0.7-0.8)	0.000	0.7	(0.7-0.7)	0.000	0.8	(0.7-0.8)	0.000	0.7	(0.6-0.7)	0.000	0.7	(0.7-0.8)	0.000
Alcohol user	1.0	(0.9-1.2)	0.862	-			1.0	(0.9-1.1)	0.980	-			1.0	(0.9-1.1)	0.882	-		
Tobacco user	0.8	(0.8-0.9)	0.000	-			0.9	(0.8-0.9)	0.000	-			0.8	(0.8-0.9)	0.000	-		

Bold variables indicate difference in RR

$ Diagnostic delay is calculated as difference between date of first diagnostic evaluation and date of screening (Figure 1)

^aRR: Adjusted Risk Ratio

[1]ASHA: Accredited Social Health Activist

[2]AWW: Anganwadi Worker

[3]RMP: Registered Medical Practitioner, an informal provider

p-value ≤ 0.05 considered significant

* While building the model for multivariate Poisson's regression analysis, all the signs and symptoms except hemoptysis were dropped because of high collinearity assessed on the basis of high VIF (Variance Inflation Factor). In addition, block and other factors like alcohol and tobacco use depicted no significant improvement in the model on the basis of likelihood ratio testing, hence, were also dropped.

For peer review only

COREQ (COnsolidated criteria for REporting Qualitative research) Checklist

A checklist of items that should be included in reports of qualitative research. You must report the page number in your manuscript where you consider each of the items listed in this checklist. If you have not included this information, either revise your manuscript accordingly before submitting or note N/A.

Topic	Item No.	Guide Questions/Description	Reported on Page No.
**Domain 1: Research team and reflexivity**			
Personal characteristics			
Interviewer/facilitator	1	Which author/s conducted the interview or focus group?	
Credentials	2	What were the researcher's credentials? E.g. PhD, MD	
Occupation	3	What was their occupation at the time of the study?	
Gender	4	Was the researcher male or female?	
Experience and training	5	What experience or training did the researcher have?	
Relationship with participants			
Relationship established	6	Was a relationship established prior to study commencement?	
Participant knowledge of the interviewer	7	What did the participants know about the researcher? e.g. personal goals, reasons for doing the research	
Interviewer characteristics	8	What characteristics were reported about the inter viewer/facilitator? e.g. Bias, assumptions, reasons and interests in the research topic	
**Domain 2: Study design**			
Theoretical framework			
Methodological orientation and Theory	9	What methodological orientation was stated to underpin the study? e.g. grounded theory, discourse analysis, ethnography, phenomenology, content analysis	
Participant selection			
Sampling	10	How were participants selected? e.g. purposive, convenience, consecutive, snowball	
Method of approach	11	How were participants approached? e.g. face-to-face, telephone, mail, email	
Sample size	12	How many participants were in the study?	
Non-participation	13	How many people refused to participate or dropped out? Reasons?	
Setting			
Setting of data collection	14	Where was the data collected? e.g. home, clinic, workplace	
Presence of non-participants	15	Was anyone else present besides the participants and researchers?	
Description of sample	16	What are the important characteristics of the sample? e.g. demographic data, date	
Data collection			
Interview guide	17	Were questions, prompts, guides provided by the authors? Was it pilot tested?	
Repeat interviews	18	Were repeat inter views carried out? If yes, how many?	
Audio/visual recording	19	Did the research use audio or visual recording to collect the data?	
Field notes	20	Were field notes made during and/or after the inter view or focus group?	
Duration	21	What was the duration of the inter views or focus group?	
Data saturation	22	Was data saturation discussed?	
Transcripts returned	23	Were transcripts returned to participants for comment and/or	

For peer review only

Topic	Item No.	Guide Questions/Description	Reported on Page No.
		correction?	
**Domain 3: analysis and findings**			
Data analysis			
Number of data coders	24	How many data coders coded the data?	
Description of the coding tree	25	Did authors provide a description of the coding tree?	
Derivation of themes	26	Were themes identified in advance or derived from the data?	
Software	27	What software, if applicable, was used to manage the data?	
Participant checking	28	Did participants provide feedback on the findings?	
Reporting			
Quotations presented	29	Were participant quotations presented to illustrate the themes/findings? Was each quotation identified? e.g. participant number	
Data and findings consistent	30	Was there consistency between the data presented and the findings?	
Clarity of major themes	31	Were major themes clearly presented in the findings?	
Clarity of minor themes	32	Is there a description of diverse cases or discussion of minor themes?	

Developed from: Tong A, Sainsbury P, Craig J. Consolidated criteria for reporting qualitative research (COREQ): a 32-item checklist for interviews and focus groups. *International Journal for Quality in Health Care*. 2007. Volume 19, Number 6: pp. 349 – 357

Once you have completed this checklist, please save a copy and upload it as part of your submission. DO NOT include this checklist as part of the main manuscript document. It must be uploaded as a separate file.

For peer review only

STROBE 2007 (v4) Statement—Checklist of items that should be included in reports of *cohort studies*

Section/Topic	Item #	Recommendation	Reported on page #
**Title and abstract**	1	(*a*) Indicate the study's design with a commonly used term in the title or the abstract	1
		(*b*) Provide in the abstract an informative and balanced summary of what was done and what was found	3, 4
**Introduction**			
Background/rationale	2	Explain the scientific background and rationale for the investigation being reported	5, 6
Objectives	3	State specific objectives, including any prespecified hypotheses	5, 6
**Methods**			
Study design	4	Present key elements of study design early in the paper	6
Setting	5	Describe the setting, locations, and relevant dates, including periods of recruitment, exposure, follow-up, and data collection	6 – 8
Participants	6	(*a*) Give the eligibility criteria, and the sources and methods of selection of participants. Describe methods of follow-up	8
		(*b*) For matched studies, give matching criteria and number of exposed and unexposed	Not applicable
Variables	7	Clearly define all outcomes, exposures, predictors, potential confounders, and effect modifiers. Give diagnostic criteria, if applicable	8, 9
Data sources/ measurement	8*	For each variable of interest, give sources of data and details of methods of assessment (measurement). Describe comparability of assessment methods if there is more than one group	8, 9
Bias	9	Describe any efforts to address potential sources of bias	9, 10
Study size	10	Explain how the study size was arrived at	8
Quantitative variables	11	Explain how quantitative variables were handled in the analyses. If applicable, describe which groupings were chosen and why	9, 10
Statistical methods	12	(*a*) Describe all statistical methods, including those used to control for confounding	9, 10
		(*b*) Describe any methods used to examine subgroups and interactions	9, 10
		(*c*) Explain how missing data were addressed	9
		(*d*) If applicable, explain how loss to follow-up was addressed	5 – 7, 9
		(*e*) Describe any sensitivity analyses	Not Applicable
**Results**			

For peer review only

Participants	13*	(a) Report numbers of individuals at each stage of study—eg numbers potentially eligible, examined for eligibility, confirmed eligible, included in the study, completing follow-up, and analysed	10 – 12
		(b) Give reasons for non-participation at each stage	10 – 17
		(c) Consider use of a flow diagram	Figure 2
Descriptive data	14*	(a) Give characteristics of study participants (eg demographic, clinical, social) and information on exposures and potential confounders	10 – 12
		(b) Indicate number of participants with missing data for each variable of interest	9
		(c) Summarise follow-up time (eg, average and total amount)	Figure 2
Outcome data	15*	Report numbers of outcome events or summary measures over time	Figure 2
Main results	16	(*a*) Give unadjusted estimates and, if applicable, confounder-adjusted estimates and their precision (eg, 95% confidence interval). Make clear which confounders were adjusted for and why they were included	Table 2
		(*b*) Report category boundaries when continuous variables were categorized	Table 2
		(*c*) If relevant, consider translating estimates of relative risk into absolute risk for a meaningful time period	Not Applicable
Other analyses	17	Report other analyses done—eg analyses of subgroups and interactions, and sensitivity analyses	Not Applicable
**Discussion**			
Key results	18	Summarise key results with reference to study objectives	17 – 18
**Limitations**			
Interpretation	20	Give a cautious overall interpretation of results considering objectives, limitations, multiplicity of analyses, results from similar studies, and other relevant evidence	19
Generalisability	21	Discuss the generalisability (external validity) of the study results	19
**Other information**			
Funding	22	Give the source of funding and the role of the funders for the present study and, if applicable, for the original study on which the present article is based	21

*Give information separately for cases and controls in case-control studies and, if applicable, for exposed and unexposed groups in cohort and cross-sectional studies.

Note: An Explanation and Elaboration article discusses each checklist item and gives methodological background and published examples of transparent reporting. The STROBE checklist is best used in conjunction with this article (freely available on the Web sites of PLoS Medicine at http://www.plosmedicine.org/, Annals of Internal Medicine at http://www.annals.org/, and Epidemiology at http://www.epidem.com/). Information on the STROBE Initiative is available at www.strobe-statement.org.

For peer review only

BMJ Open

Pre-diagnostic loss to follow-up in an active case finding tuberculosis program: a mixed-methods study from rural Bihar, India

Journal:	*BMJ Open*
Manuscript ID	bmjopen-2019-033706.R2
Article Type:	Original research
Date Submitted by the Author:	02-Mar-2020
Complete List of Authors:	Garg, Tushar; Innovators In Health, Research<br>Gupta, Vivek; All India Institute of Medical Sciences, Dr. R.P Centre for Ophthalmic Sciences<br>Sen, Dyuti; Innovators In Health, Operations<br>Verma, Madhur ; All India Institute of Medical Sciences, Department of Community and Family Medicine<br>Brouwer, Miranda; PHTB Consult, Consulting<br>Mishra, Rajeshwar; Innovators In Health, Research; Centre for Development of Human Initiatives<br>Bhardwaj, Manish; Innovators In Health, Operations
**Primary Subject Heading**:	Public health
Secondary Subject Heading:	Infectious diseases, Global health
Keywords:	pre-diagnostic loss to follow-up, cascade of care, ASHA, Accredited Social Health Activist, Community health worker, screening

SCHOLARONE™
Manuscripts

I, the Submitting Author has the right to grant and does grant on behalf of all authors of the Work (as defined in the below author licence), an exclusive licence and/or a non-exclusive licence for contributions from authors who are: i) UK Crown employees; ii) where BMJ has agreed a CC-BY licence shall apply, and/or iii) in accordance with the terms applicable for US Federal Government officers or employees acting as part of their official duties; on a worldwide, perpetual, irrevocable, royalty-free basis to BMJ Publishing Group Ltd ("BMJ") its licensees and where the relevant Journal is co-owned by BMJ to the co-owners of the Journal, to publish the Work in this journal and any other BMJ products and to exploit all rights, as set out in our licence.

The Submitting Author accepts and understands that any supply made under these terms is made by BMJ to the Submitting Author unless you are acting as an employee on behalf of your employer or a postgraduate student of an affiliated institution which is paying any applicable article publishing charge ("APC") for Open Access articles. Where the Submitting Author wishes to make the Work available on an Open Access basis (and intends to pay the relevant APC), the terms of reuse of such Open Access shall be governed by a Creative Commons licence – details of these licences and which Creative Commons licence will apply to this Work are set out in our licence referred to above.

Other than as permitted in any relevant BMJ Author's Self Archiving Policies, I confirm this Work has not been accepted for publication elsewhere, is not being considered for publication elsewhere and does not duplicate material already published. I confirm all authors consent to publication of this Work and authorise the granting of this licence.

For peer review only

TITLE

Pre-diagnostic loss to follow-up in an active case finding tuberculosis program: a mixed-methods study from rural Bihar, India

MANUSCRIPT TYPE

Research article

AUTHORS

Tushar Garg[1], Vivek Gupta[2], Dyuti Sen[1], Madhur Verma[3], Miranda Brouwer[4], Rajeshwar Mishra[1,5], Manish Bhardwaj[1]

1. Innovators In Health, Patna, India
2. Dr R P Centre for Ophthalmic Sciences, All India Institute of Medical Sciences, New Delhi, India
3. All India Institute of Medical Sciences, Bathinda, India
4. PHTB Consult, Tilburg, The Netherlands
5. Centre for Development of Human Initiatives, Jalpaiguri, India

ADDRESS FOR CORRESPONDENCE

Tushar Garg,
Innovators In Health, #176, Ground Floor, Road #2,
New Pataliputra Colony, Patna – 800013. Bihar. India.
Email: tgarg@innovatorsinhealth.org
Phone: +91 98286 09562

MANUSCRIPT DETAILS

Abstract: 293 words

Manuscript: 5001 words

Tables: 2 + 1 (supplementary)

For peer review only - http://bmjopen.bmj.com/site/about/guidelines.xhtml

For peer review only

Figures: 3

KEYWORDS

Pre-diagnostic loss to follow-up; cascade of care; screening; ASHA; Accredited Social Health Activist; community health worker

For peer review only

For peer review only - http://bmjopen.bmj.com/site/about/guidelines.xhtml

ABSTRACT

objective

To quantify the pre-diagnostic loss to follow-up (PDLFU) in an active case finding (ACF) tuberculosis (TB) program, and identify the barriers and enablers in undergoing diagnostic evaluation.

design

Explanatory mixed-methods design.

setting

A rural population of 1.02 million in the Samastipur district of Bihar, India.

participants

Based on their knowledge of health status of families, community health workers or CHWs (called Accredited Social Health Activist or ASHA locally) and informal providers referred people to the program. The field coordinators (FC) in the program screened the referrals for TB symptoms to identify presumptive TB cases. CHWs accompanied the presumptive TB patients to free diagnostic evaluation and a transport allowance was given to the patients. Thereafter, CHWs initiated and supported the treatment of confirmed cases. We included 13,395 community referrals received between January and December 2018. To understand the reasons of the PDLFU, we conducted in-depth interviews with patients who were evaluated (n = 3), patients who were not evaluated (n = 4), and focus group discussions with the CHWs (n = 2) and FCs (n = 1).

outcome measures

Proportion and characteristics of PDLFU, and association of demographic and symptom characteristics with diagnostic evaluation.

For peer review only - http://bmjopen.bmj.com/site/about/guidelines.xhtml

results

A total of 11,146 presumptive TB cases were identified between January and December 2018, out of which 4912 (44.1%) underwent diagnostic evaluation. In addition to the free TB services in the public sector, the key enablers were CHW accompaniment and support. The major barriers identified were misinformation and stigma, deficient family and health provider support, transport challenges, and poor services in the public health system.

conclusion

Finding the missing cases will require patient-centric diagnostic services and urgent reform in the health system. A community-oriented intervention focusing on stigma, misinformation, and patient support will be critical to its success.

STRENGTHS AND LIMITATIONS OF THIS STUDY

1. First such study to explore the reasons for pre-diagnostic loss to follow-up in a TB program.
2. A mixed-methods design which includes the views of both patients and CHWs.
3. The study utilized operational data from a routine programmatic setting.
4. Since intervention removed some barriers, all findings are not necessarily generalizable.
5. No record of the actual number of people screened before being referred to the program.

For peer review only

INTRODUCTION

The End TB strategy targets an 80% reduction in tuberculosis (TB) incidence by 2030.[1] However, as many as 4.1 million (39%) patients globally are not notified, indicating a mix of under-reporting and under-diagnosis.[2] With an incidence rate of 199 per 100,000 population in 2018, India alone has about a million such “missing” cases.[3]

Most TB patients are identified through passive case-finding where patients reach out to a provider.[4] However, it leads to delays in diagnosis, and loss to follow-up (LFU) during diagnostic and treatment phases.[5] In fact, the Indian public sector cascade estimates a 28% LFU at test access amongst incident cases.[6] The major challenges in diagnosis are poor geographical and financial access to healthcare, and failure to test when people do present at the facility.[7] Active case-finding (ACF) can solve some of these challenges by proactively taking health services to the people in the community.[2,8]

Diverse ACF strategies have demonstrated increased TB case detection with early diagnosis.[4,9–11] Nonetheless, multiple ACF interventions report a high pre-diagnostic loss to follow-up (PDLFU). An Indian study reported only 22% of the people with presumptive TB reaching a microscopy center, while a study in Myanmar estimated only 51.4% of patients with abnormal chest X-ray (CXR) getting their sputum examined.[12,13] Although the quantum of the PDLFU is known, its underlying causes are yet to be investigated.

We studied the PDLFU in a community-based ACF program implemented in India in 2018. The objectives were to assess proportion and characteristics of PDLFU cases, identify the risk factors, and understand the reasons for the PDLFU, including the barriers and enablers in accessing diagnostic evaluation.

METHODS

study design

We used an explanatory mixed-methods study design, where the quantitative phase (cohort analysis) was followed by a qualitative phase (descriptive design).[14]

For peer review only

For peer review only - http://bmjopen.bmj.com/site/about/guidelines.xhtml

study setting

General setting

The study was conducted in the Sarairanjan, Bibhutipur, and Ujiarpur blocks (equivalent to TB Unit or TU) of Samastipur district in Bihar with a combined population of 1,021,483. The highest earning member in 69.8% households earned less than INR 60,000 annually (~USD 852) and 63.5% population was literate.[15] Females literacy rate was 20% lower than males, and infant mortality rate (IMR) per 1000 live births was 60 for females against that of 48 for males.[16] In 2017, the public TB case notification rate in the district was 55 per 100,000 population with a pre-treatment loss to follow-up of 25%.[17] Furthermore, patients preferred private sector to access TB care.[18]

The population was serviced by 12 primary health centres (PHC) in the public health system (PHS).[19] Within the Revised National TB Control Program (RNTCP), 4 designated microscopy centres (DMC) run by laboratory technicians provided sputum microscopy. A Senior Treatment Supervisor (STS) managed each TU and a Senior Treatment Laboratory Supervisor (STLS) managed a group of DMCs.

The TB care in the community were provided by Accredited Social Health Activist (ASHA). ASHA is a community health worker (CHW) assigned for every 1000 population within the National Health Mission (NHM). Her role is to be a facilitator, mobilizer, and community service provider for various public health programs.[20] ASHA receives an activity-based remuneration of INR 1000 – 1500 (~USD 14 – 21) for drug-sensitive TB treatment support, and INR 5000 (~USD 72) for drug-resistant treatment support.[21]

Anganwadi workers (AWW) are a CHW in the Integrated Child Development Services program and receive a fixed monthly honorarium. Through Angadwadi Centres, they provide early childhood care and education for children up to 6 years. However, they are directly not involved in TB care.[22]

For peer review only - http://bmjopen.bmj.com/site/about/guidelines.xhtml

Specific setting

Supported by TB REACH, we implemented a community-based ACF project in collaboration with RNTCP and NHM. ACF was added to the routine RNTCP program with interventions in the community as well as the public health system. Its key components were community referral, symptom-based screening at patient's home, transport allowance to patients, free diagnostic evaluation, and diagnosis and treatment assisted by ASHAs. There were no consistent ACF campaigns prior to this program in our study population.

We engaged various health workers, including ASHA, AWW and RMP (informal providers known as Registered Medical Practitioner). A TB awareness meeting was organized in the community to kickstart the program. The health workers and laypersons were asked to refer people who may have TB to the program. Since they are aware of the health status of families in their community, they were well-placed to identify such people in their routine work. These community referrals were reported to a field coordinator (FC) of the project, who screened them at their home for TB symptoms. The presumptive TB cases were those with one or more of the following symptoms: cough of ≥ 2 weeks, sputum in cough, hemoptysis in last 6 months, chest pain in last 1 month, fever of ≥ 2 weeks, night sweats for ≥ 2 weeks, severe weight loss in the last 3 months, swelling in a lymph node.

The diagnostic algorithm in the study followed RNTCP's recommendations.[21] All presumptive TB cases were tested using sputum microscopy and chest X-ray (CXR). Patients with a positive smear, or those with a negative smear but abnormal CXR, or patients with a negative smear but clinically suspect were offered a GeneXpert test using sputum collection and transport mechanism. ASHAs assisted presumptive TB patients in reaching the diagnostic centres and accompanied them through the diagnostic process. Patients received a transport allowance and diagnostic tests were free of cost. If the PHS physician confirmed TB diagnosis, treatment was initiated under ASHA's supervision. ASHA delivered drugs to the patient, ensured follow-up tests and treatment adherence, and monitored for adverse effects.[Figure 1]

For each confirmed TB case, a conditional incentive of INR 200 (~USD 3) for community referral and INR 300 (~USD 5) for diagnostic support was given to the CHWs. These incentives to the CHW

For peer review only - http://bmjopen.bmj.com/site/about/guidelines.xhtml

were in addition to the RNTCP's treatment support incentives. The project's FCs received a fixed monthly salary.

The project used a Management Information System (MIS) developed in Microsoft Excel 2016® which maintained individual patient data electronically. On receipt of a community referral, the FC enlisted them in a referral register with a unique ID and a separate form was completed for screening. The status of each community referral was tracked using the referral register. For presumptive TB cases undergoing diagnostic evaluation, an individual folder with their forms and diagnostic reports was maintained. Data were entered weekly into the MIS and at least 20% entries were verified monthly in a two-person formation to assess data entry quality.

study population and study period

Quantitative

All community referrals between January 01, 2018 to December 31, 2018 in the 3 selected blocks of Samastipur district were included in the study. There was no sampling involved.

Qualitative

Presumptive TB cases referred for diagnostic evaluation were interviewed. In-depth interviews were conducted with 3 presumptive TB cases who were evaluated (2 females, 1 male, age range: 17 – 30 years) and 4 presumptive cases who were not evaluated (2 females, 2 males, age range: 6 – 65 years). A purposive sample of ASHAs and field coordinators was selected for the focus group discussions (FGD). Three FGDs were conducted with a group of 8 ASHAs (all females, age range: 24 – 49), 11 ASHAs (all females, age range: 22 – 54), and 9 field coordinators (6 females, 3 males, age range: 20 – 38). The average duration was 36 minutes (range 23 – 51 minutes). A diverse sample of males and females across age groups was purposively selected for diversity and interviewed till saturation was achieved.

For peer review only - http://bmjopen.bmj.com/site/about/guidelines.xhtml

data variables, sources of data and data collection

Quantitative

Data on the community referrals' characteristics (location, referral source, age, gender), screening criteria (date of screening, symptoms, outcome), and diagnostic evaluation (date of test, type of test) were extracted from the MIS into a structured proforma. The data extracted from the MIS was deidentified before its export for analysis. [Figure 1]

Qualitative

Presumptive TB cases were interviewed by TG (a male medical doctor trained in qualitative research) and DS (a female economist trained in qualitative research), and FGDs were conducted by TG and RM (a male professor of social psychology). All the three investigators have adequate experience of the socio-cultural context of the region, and understanding of TB programs. After obtaining a participant's consent, the interviews were conducted at a time and place convenient for the participant and their privacy was ensured. The regional vernacular was used for interactions and it was recorded after obtaining consent. The objectives of the study were explained to the participants. No participant denied permission for interview and there were no repeat interviews. A topic guide was used for FGDs and an interview guide for in-depth interviews to explore the enabling factors and barriers for diagnostic evaluation. Appropriate probes were used for clarity and to elicit information. The information was debriefed for participant validation after the interview, but the transcripts were not returned to them.

analysis and statistics

Quantitative

The data were analysed using EpiData Analysis (version 2.2.2.183, EpiData Association, Odense, Denmark) and Stata (version 15.1, StataCorp LLC, College Station, Texas, USA). Patients not undergoing a diagnostic test for TB within 30 days of referral were considered PDLFU. Patients with diagnostic test after 30 days (n = 140) were excluded from the diagnostic evaluation category. In the event that patients had a diagnostic test performed prior to community referral, the time to diagnostic visit was set as 0 days (n = 486). Age was missing for 242 community

For peer review only - http://bmjopen.bmj.com/site/about/guidelines.xhtml

referrals. As applicable, variables were summarized with mean (and standard deviation or SD) or median (and inter-quartile range or IQR) based on statistical distribution of data, or frequencies and percentages. Association of demographic and symptom characteristics with diagnostic evaluation was analyzed using χ2-square test and unadjusted relative risk (RR) with 95% confidence interval was calculated.

A multivariate analysis using Poisson's regression was used to calculate adjusted relative risk (aRR) with 95% confidence interval. Collinearity was assessed using variance inflation factors during model building. Likelihood ratio tests were used to identify factors that contributed significantly to the model. P-value ≤ 0.05 was considered statistically significant. A sensitivity analysis (univariate and multivariate) was performed by excluding patients whose diagnostic test was performed prior to screening (n = 486) and also by classifying patients who underwent diagnostic evaluation after 30 days as non-PDLFU (n = 140).

For peer review only

Qualitative

The transcripts were prepared on the same day using audio recording and field notes by TG. Manual descriptive content analysis was performed by two independent, trained researchers (TG, DS) to generate categories and themes.[23] Any discrepancies between the two were resolved through discussion. These were discussed and reviewed by RM to avoid subjective bias. The codes and themes were related back to the original data to ensure that the results reflect the data.[24]

patient and public involvement

Patients were neither involved in the study design nor in the interpretation of patient relevant outcomes. Nonetheless, patient's views were sought in the qualitative interviews and included in the results. The results of this study will be communicated to the patients and the public through a vernacular newsletter.

For peer review only - http://bmjopen.bmj.com/site/about/guidelines.xhtml

RESULTS

care cascade and characteristics of presumptive TB cases

We received a total of 13,395 community referrals, out of which 90.9% (n=12,180) were screened for symptoms. Of those screened, 91.5% (n=11,146) were presumptive TB cases, and referred for diagnostic evaluation. (Figure 2)

There was nearly equal representation of presumptive TB cases from all the 3 blocks, and ASHAs identified most of them (75.6%). The mean age of presumptive TB cases was 35 years with majority in the 15 – 44 age group (41.8%). There were more males (52.2%) than females. (Table 1)

Table 1. Demographic profile of referrals, screened cases, and presumptive TB cases identified between January 2018 and December 2018 in Samastipur, India.

Characteristics	Community referrals, n(%)		Screened for TB, n(%)		Presumptive TB cases, n(%)	
**Total**	13395		12180		11146	
**Block**						
Ujiarpur	4275	(31.9%)	3926	(32.2%)	3620	(32.5%)
Bibhutipur	4609	(34.4%)	3994	(32.8%)	3673	(33%)
Sarairanjan	4511	(33.7%)	4260	(35.0%)	3853	(34.5%)
**Age**						
<15	2847	(21.3%)	2611	(21.4%)	2385	(21.4%)
15-44	5641	(42.1%)	5147	(42.3%)	4661	(41.8%)
45-64	3330	(24.9%)	3032	(24.9%)	2789	(25.0%)
≥65	1335	(10.0%)	1243	(10.2%)	1169	(10.5%)
Missing	242	(1.8%)	147	(1.2%)	142	(1.3%)
**Gender**						
Male	6973	(52.1%)	6391	(52.5%)	5827	(52.2%)
Female	6422	(47.9%)	5789	(47.5%)	5319	(47.7%)
**Source of referral**						
ASHA[1]	10091	(75.3%)	9210	(75.6%)	8428	(75.6%)
AWW[2]	105	(0.8%)	101	(0.8%)	88	(0.8%)
RMP[3]	724	(5.4%)	661	(5.4%)	617	(5.5%)
Community	2475	(18.5%)	2208	(18.1%)	2013	(18.1%)

[1]ASHA: Accredited Social Health Activist

[2]AWW: Anganwadi Worker

For peer review only

For peer review only - http://bmjopen.bmj.com/site/about/guidelines.xhtml

[3]RMP: Registered Medical Practitioner, an informal provider

The most common symptoms among presumptive TB cases were cough of ≥ 2 weeks (79.8%), severe weight loss in the last 3 months (74.6%), fever of ≥ 2 weeks (73.9%), and chest pain in the last 1 month (63.4%), while hemoptysis in the last 6 months (12.1%) was the least common. Nearly one-fifth of the presumptive TB cases reported a previous history of anti-TB treatment (22.4%), and 20.5% were tobacco users. (Table 2)

Table 2: Characteristics of and risk factor for pre-diagnostic loss to follow up (PDLFU) among presumptive TB cases referred for diagnostic evaluation between January 2018 and December 2018 in Samastipur, India.

For peer review only

Characteristics	Presumptive TB cases, n	Not evaluated, n(%)	Unadjusted RR (95% CI)	p-value	Adjusted RR*(95% CI)	p-value
**Total**	11146	6234 (55.9%)				
**Block**						
Ujiarpur	3620	2060 (56.9%)	1.0 (1.0-1.1)	0.022	-	
Bibhutipur	3673	2083 (56.7%)	1.0 (1.0-1.1)	0.033	-	
Sarairanjan	3853	2091 (54.3%)	Ref		-	
**Age**						
<15	2385	1625 (68.1%)	1.4 (1.3-1.4)	<0.001	1.2 (1.2-1.3)	<0.001
15-44	4661	2459 (52.8%)	1.0 (1.0-1.1)	0.139	1.0 (1.0-1.1)	0.366
45-64	2789	1464 (52.5%)	1.0 (1.0-1.1)	0.212	1.0 (1.0-1.1)	0.164
≥65	1169	588 (50.3%)	Ref		Ref	
**Gender**						
Female	5827	3375 (57.9%)	Ref		Ref	
Male	5319	2859 (53.8%)	1.1 (1.0-1.1)	<0.001	1.1 (1.0-1.1)	0.001
**Source of referral**						
ASHA[1]	8428	4690 (55.7%)	1.3 (1.2-1.4)	<0.001	1.2 (1.1-1.3)	<0.001
AWW[2]	88	52 (59.1%)	1.3 (1.1-1.6)	0.003	1.2 (1.0-1.5)	0.025
RMP[3]	617	271 (43.9%)	Ref		Ref	
Community	2013	1221 (60.7%)	1.4 (1.3-1.5)	<0.001	1.3 (1.2-1.4)	<0.001
**Previous history of anti-TB treatment**						
Yes	2501	1034 (41.3%)	0.7 (0.6-0.7)	<0.001	0.7 (0.7-0.8)	<0.001
No	8645	5200 (60.2%)	Ref			
**Presence of Signs and symptoms**[#]						
Haemoptysis in last 6 months	1346	494 (36.7%)	0.6 (0.6-0.7)	<0.001	0.7 (0.6-0.7)	<0.001
Cough ≥ 2 weeks	8895	4514 (50.8%)	0.7 (0.6-0.7)	<0.001	-	

For peer review only - http://bmjopen.bmj.com/site/about/guidelines.xhtml

Sputum	6184	2779	(44.9%)	0.6	(0.6-0.7)	<0.001	-
Chest pain in last 1 month	7061	3260	(46.2%)	0.6	(0.6-0.7)	<0.001	-
Fever ≥ 2 weeks	8242	4289	(52.0%)	0.8	(0.8-0.8)	<0.001	-
Night sweats ≥ 2 weeks	3730	1724	(46.2%)	0.8	(0.7-0.8)	<0.001	-
Severe weight loss in last 3 months	8318	4254	(51.1%)	0.7	(0.7-0.8)	<0.001	-
Swelling in a lymph node	2067	1517	(73.4%)	1.4	(1.4-1.5)	<0.001	-
**Other factors[#]**							
Alcohol user	159	90	(56.6%)	1.0	(0.9-1.2)	0.862	-
Tobacco user	2280	1109	(48.6%)	0.8	(0.8-0.9)	<0.001	-

[1]ASHA: Accredited Social Health Activist

[2]AWW: Anganwadi Worker

[3]RMP: Registered Medical Practitioner, an informal provider

Age is missing for 142 presumptive cases and 98 not evaluated cases.

All % are row percentages.

P-value ≤ 0.05 considered significant

[*] While building the model for multivariate Poisson's regression analysis, all the signs and symptoms except hemoptysis were dropped because of high collinearity assessed on the basis of high Variance Inflation Factor (VIF). The final variables in the model were selected on the basis of likelihood ratio testing.

[#] For signs, symptoms and other factors, the absence of that characteristic sign and symptoms was considered as the reference category.

characteristics of pre-diagnostic LFU and associated risk factors

Nearly 44% (n=4912) of the presumptive TB cases received diagnostic evaluation. The pre-diagnostic LFU was highest among younger (< 15 year) presumptive TB cases (68.1%), while lesser among those who were referred by an RMP (43.9%) and complained of hemoptysis in the last 6 months (36.7%). The median time to diagnosis was 1 day (IQR = 3).

On multivariate analysis, presumptive TB cases who were < 15 years of age (aRR = 1.2, 95% CI: 1.2-1.3, p = 0.00) were more likely to be PDLFU. Previous history of Tuberculosis treatment (aRR = 0.7, 95% CI: 0.7-0.8, p = 0.00) and hemoptysis in last 6 months (aRR = 0.7, 95% CI: 0.6-0.7, p = 0.00) decreased the chances of PDLFU. (Table 2)

In the sensitivity analysis, no change was observed in association of the risk factors with PDLFU on either excluding patients who were diagnosed prior to screening or classifying patients who underwent diagnostic evaluation after 30 days as non-PDLFU. (Supplementary table 1)

For peer review only - http://bmjopen.bmj.com/site/about/guidelines.xhtml

For peer review only

Enablers to access the first diagnostic evaluation

In the interviews, patients reported the following enablers for diagnostic evaluation: transport allowance for travel to the hospital, free services in the PHS and knowledge of the PHS procedures, accompaniment of ASHA and her assistance in the diagnostic process, and ASHA's understanding of the PHS functioning. (Figure 3)

> *"I started facing financial problems after going to the private hospital… Had I known the quality of care in the government hospital earlier, I would have come here. It helped that I didn't have to spend any money. I went by a vehicle to the PHC and consulted the doctor for free of cost."* (23 years old male patient)

> *"I didn't have to go to the hospital after submitting the sample for diagnostics… ASHA took me to the hospital and now sends the medicine… I was bony and couldn't even move a step earlier… I became better only from these government drugs; rest were useless. Between RMP and private clinic, we spent nearly [illegible]20,000* (~ USD 292)*."* (17 years old female TB patient on medication)

> *"When I had chest pain, ASHA told me that we'll have to go to the PHC, tests will be free, and I'll get return fare from my home. I thought I'll get better and decided to go. I got a blood test and x-ray done. Reports were normal and I got some medicines. I felt better… If you know the hospital and workers there, it is easier to get things done. Else, you have to spend a lot of time."* (30 years old female patient)

The ASHAs and the Field Coordinators (FCs) expressed transport allowance, incentive to ASHA, positive patient experience, and patient accompaniment as enablers. (Figure 3)

> *"If one patient becomes better, she also advises others. Even if we don't know, the patient who becomes better asks their ill neighbor to call the ASHA for everything. Our mobile number is available in every household."* (ASHA)

> *"Sometimes, patients do not have any shared conveyance from their home. In such cases, patients are unable to come, and transport allowance or hiring an autorickshaw for, say 10 patients of the village, really helps. That way, they get consultation and lab tests at*

For peer review only

For peer review only - http://bmjopen.bmj.com/site/about/guidelines.xhtml

PHC, then chest x-ray at another diagnostic facility, and get dropped at their home." (Field coordinator)

"ASHA's income is from incentives, and if she gets incentives in time, she takes up those activities." (Field coordinator)

barriers to access the first diagnostic evaluation

Barriers were coded from the interview and FGD transcripts into 5 categories and 27 codes. These barriers are listed in the Figure 3 and described below.

Category 1: Logistics-related

As per patients, public transport was not available in certain locations and some patients mentioned their inability to pay for it even where it was available. They also reported not knowing the location of the diagnostic center.

"I don't know where the diagnostic center is. From my village, I have to walk for 3 – 4 km before I find any transport." (65 years old woman)

The ASHA and FC corroborated that transport and insufficient support for certain patient population like disabled people was a key logistics-related challenge.

"Travel options are also limited for some areas. Either it is not readily available or you have to walk before anything can be found. They don't have the money to reserve an entire shared vehicle. If the patient is disabled or elderly, they face even more problems." (Field coordinator)

Category 2: Health system-related

According to patients, the care at PHC was slow and took a lot of time. They said either the PHC remained closed or they didn't reach the PHC in working hours. At other times when they reached, the providers were not available at the facility.

"I had to go 2-3 times to the PHC before all the tests were completed and reports arrived. Only then I could get the medicines." (17 years old female patient)

For peer review only - http://bmjopen.bmj.com/site/about/guidelines.xhtml

> *"We have to run-around a lot in the government hospital. There are huge buildings and it is difficult to figure out what happens where alone."* (30 years old male patient)

The health workers reported unavailability of some diagnostic services in the PHC and the high cost of getting these in the private sector. ASHA's remuneration for providing TB care services was irregular and unpaid. They indicated that poor patient experience at the PHC also deterred others and that the public put a stronger faith in the private sector.

> *"Patient doesn't know where the registration desk is, where the doctor sits, where the labs and x-ray are done. We have to go with them to get everything completed quickly."* (ASHA)

> *"I have provided treatment to 20 patients, but I haven't been paid for it. It is only in the recent 1-2 years that there has been some improvement. We are not told properly how to fill the document and what to submit for release of payments. All our papers were taken and nothing happened. Earlier we used to only counsel the patient to go for diagnostics, but now we come with them if we get money for helping in diagnosis."* (ASHA)

> *"If patient come on a given day and their work doesn't get done, they won't come again. Coming again will mean foregoing another day's wage. They will say that ASHA cheated them and nothing happened at the hospital and nothing is available at the PHC ever. If they go to a private clinic, everything is done in one day and they get the medicine… They also prefer private." (ASHA)*

Category 3: Health provider-related

Patients described health worker strikes in the field and at the PHC as a barrier. One patient also reported that ASHA doesn't come to their home.

> *"I took the sputum to the PHC, but nothing was done. PHC was on strike for 15 days. The unabated cough and fever didn't let me sleep. When I felt that I would die, I took an injection from the RMP and drank 5 cough syrups. Only then was there any relief."* (65 years old male patient)

For peer review only

For peer review only - http://bmjopen.bmj.com/site/about/guidelines.xhtml

"What will happen after going to the PHC? I go to the hospital and come back with nothing. Even ASHA doesn't come. I am going to lose everything in this hustle and bustle." (35 years old female patient)

The health workers reported RMPs misguiding the patients, low morale of the workers, and poor health provider behavior at the PHC as barriers.

"RMPs misguide the patients a lot. They give medications and injections without any concern. They are not at risk even if the patient dies. If patient becomes serious and they have made money, they will refer them to hospital. They roam through the villages and people get medicine at their doorstep. Someone who is a laborer, their income for the day is saved." (ASHA)

"When we take the patient to the PHC, lab technician will return the sample and asks to come on another day... Doctor will also not write any medication and don't know the updated guidelines… We have to then listen from our patient." (ASHA)

For peer review only

Category 4: Family-related

Patients indicated not knowing the extent of available services in the PHS. Some described severe indebtedness of the family and not having any money for healthcare. According to them, insufficient family support and absence of a caretaker in the family were also barriers.

"I live with my two granddaughters and my son works as a watchman in New Delhi. The money is irregular. If I've no money, I won't go. In November, I didn't have money and couldn't go… For consulting with the RMP, I had to take a loan of ₹150 (~ USD 2.2). *After a week, I've been able to return only ₹50."* (65 years old female patient)

"Both my kids are alone. My husband has migrated for work. He says that take the kids to a senior doctor in a private hospital. It'll cost at least ₹3000 (~ USD 44) *and I am waiting for it… There is only my old mother-in-law in the house besides me. How will I leave them alone at home?"* (Mother of a 6 years old patient)

For peer review only - http://bmjopen.bmj.com/site/about/guidelines.xhtml

Health workers mentioned permission from the head of the family to visit facility, restricted mobility of women in the families as compared to men, and family's concern only if symptoms were severe as barriers.

"Patients say that we'll ask the head of the family and come only if they assent. Sometimes they are not sure of the quality of care in the PHS. We also counsel the guardian… This happens more often for women. Men are independent; they don't have to ask the head or the wife… It is also difficult for women. They have to complete household chores and care for the kids before leaving home for the facility. Often, by the time they reach, the facility will close." (ASHA)

"If the symptoms are not severe, then patient takes some medicine from RMP at their home and doesn't want to come for a test." (Field coordinator)

Category 5: Patient-related

Patients indicated denial of suffering from TB.

"I don't need any diagnostics. I didn't have TB ever in my family. This is all because of gas." (65 years old patient)

As per health workers, some patients had a fear of TB. They also reported discrimination by the community against TB patients.

"We don't say TB at first. If we say it, people hesitate. Some are offended that we have said TB is a possible disease for them. We've to tell them gently. One sputum positive patient said that she was hurt that I said she has TB." (ASHA)

"A lot of people prefer to keep their disease status hidden. TB is considered contagious and people don't want neighbors to know of it. Earlier, even patient's utensils and bedding were kept separate." (ASHA)

DISCUSSION

This is the first such study to estimate the PDLFU in an ACF program while also investigating the barriers and enablers in accessing diagnostic evaluation. About 44% of the presumptive TB cases

For peer review only - http://bmjopen.bmj.com/site/about/guidelines.xhtml

For peer review only

couldn't undergo diagnostic evaluation. Provision of transport allowance for patients, accompaniment and support of ASHA, incentives to ASHA, and knowledge of the PHS procedures emerged as key enablers. The major barriers were misinformation and stigma, insufficient family and health provider support, transport despite an allowance, inadequate health services, and poor faith in the public sector.

The strengths of the study are as follows. First, we used a mixed-methods design in which the quantitative and qualitative components complement each other. The qualitative component explored hitherto unresearched reasons for the PDLFU and recorded the perspectives of both patients and CHWs. Second, the study was conducted in an ACF program using routinely collected data, hence, reflecting the field reality.

There are several limitations as well. First, the ACF intervention was an addon to the RNTCP program with extra provisions like travel allowance. Since the project removed certain barriers in undergoing diagnostic evaluation, the analysis is not necessarily generalizable to routine care. Second, the CHWs and RMPs were likely filtering patients before they were referred to the program. Therefore, the true number of people screened would be higher than that reported in the study. Similarly, the proportion who were identified as presumptive TB cases in the study is likely much higher than in the overall community. Third, while the presumptive TB cases receiving a test was known, there was no information on cases reaching the facility but not receiving a test, or people not completing the diagnostic process.

While 96.6% of the presumptive TB cases received a CXR in the mobile van in the ACF project in Myanmar, only 51.4% of the cases referred for sputum microscopy to the health facility followed through.[12] Similarly, in an ACF campaign in India, only 22% referred people made it to the DMC, but about 54% received sputum microscopy with provision of specimen transport.[13] In comparison, 41.1% of the presumptive TB cases in our study underwent diagnostic evaluation, which included 81.6% and 50.6% receiving a CXR and sputum microscopy respectively at a health facility. Referral with adequate support like that through ASHA accompaniment and community-oriented TB service delivery like mobile vans can complement each other in diagnostic evaluation. In the case of mobile CXR, immediate availability of a diagnostic test for which people didn't have to travel encouraged uptake. Furthermore, specimen transport helped in

For peer review only - http://bmjopen.bmj.com/site/about/guidelines.xhtml

For peer review only

microbiological testing by moving the sample instead of the patient, but challenges like refrigeration and sample preparation are aplenty.[25] Point-of-care diagnostic tests like the upcoming GeneXpert Omni and Truenat can improve uptake of microbiological testing in such scenarios.[26,27] The Zimbabwe study reported place of residence (rural) and type of facility (private) as important risk factor for pre-diagnostic LFU.[28] While we found association of PDLFU with various risk factors like age and previous history of TB, they are of limited public health or clinical importance.

Nonetheless, qualitative findings provide rich insights. The transport allowance, free diagnostic services, ASHA's accompaniment supported the patients. While covering the financial costs of the patient certainly helps, opportunity cost like lost wages also need to be considered.[29,30] In fact, barriers like indebtedness of family can exacerbate the opportunity cost, consequently, restricting the benefits of covering financial cost through transport allowance and free diagnostics.[31] Furthermore, systemic factors like unavailability of transport options limits the effectiveness of such solutions.[32,33] The health system and health provider issues like cumbersome processes in health facilities and poor provider behavior resonated in our findings.[34] These patient narratives add explanation to people's preference for private sector and informal providers even though affordable services are available in the PHS. People's trust in the provider is a crucial factor in determining their preferred provider, which was also corroborated by patients in our study.[35] The disparity is further accentuated by variability of ASHAs' quality.[36] In our study, a patient complained of their ASHA's lackadaisical attitude and another failed to find the diagnostic center indicating that their ASHA didn't help. This difference between ASHAs was also confirmed by the field coordinators: not all ASHAs are equally interested or equally capable. This will need attention in programs involving ASHA to encourage their active engagement.

From patients' perspective, stigma, misinformation, and insufficient family support emerged as important barriers. The support to patients is often dependent on their family and its situation. For instance, women whose husband migrated for work have to manage the household with limited finances and find it difficult to make time during the labs' visiting hours. Additional challenges of a patriarchal society like restricted mobility and family elder's permission

For peer review only - http://bmjopen.bmj.com/site/about/guidelines.xhtml

compound the problem for women.[37] Similarly, older and disabled people may not even have the essential family or systemic support. Furthermore, the misinformation and stigma in the community against TB force the patients to keep the diagnosis to themselves. Although the ASHAs reported that stigmatizing practices have improved, a thorough investigation needs to be undertaken to further reduce the negative influence of stigma on TB care and treatment.

We suggest a two-pronged approach to improve the situation: a community-level effort focusing on stigma and patient support, and a systemic reform in the health system. A sustained campaign on awareness of the disease along with services available for such patients is a must. As the campaign corrects misinformation, it creates a viable climate to tackle the stigma. Delivering such a campaign effectively will need involvement of all sections of the community, in particular the opinion shapers and influencers in the region.[38] The campaigns will need contextualization based on the population's language and culture. Both informal and formal community association, like religious groups or self-help groups, can be a possible vehicle.[39] In fact, such groups along with patient support network can be leveraged to create a support system for patients as well.[40] The second prong hinges on building a system of seamless care and improving quality in the public sector.[41,42] Private sector interventions in TB care have demonstrated seamless care through processes like social franchising for diagnostic tests.[43] Similarly, trusted informal providers deliver services at the patient's doorstep at a convenient time, thus, also reducing the opportunity costs. PHS needs to learn from the private and informal providers to be a competitive source of healthcare services.[44–46] It will require investment in taking healthcare closer to the community through steps like upgrading the CHWs, adequate supportive supervision, and instituting health outposts.[47] In addition, quality needs to be a core principle in the health system, both private and public. Involving people in assessing the local health system by social audits and patient feedback, and embedding quality in the performance indictor of the health system and health workers can be a starting step.

In conclusion, these findings are relevant to the broader primary health services in addition to the TB program. While improving care in the public health system is essential to make it competitive, involving the community and considering sociocultural factors will be critical to its success.

For peer review only - http://bmjopen.bmj.com/site/about/guidelines.xhtml

470 **ACKNOWLEDGEMENT**

471 We are grateful for the support of RNTCP, Government of Bihar, State TB Officer Dr. (Major) K.
472 N. Sahai, Communicable Disease Officer Dr. Sree Ram Prasad, and District Health Society,
473 Samastipur. We thank Sriram Selvaraju, Anthony D. Harries, Dick Menzies, and participants of the
474 TB Research Methods course at McGill Summer Institute 2018 for their comments on the
475 protocol, and Amol Dongre for his feedback on the qualitative results. The program couldn't have
476 been implemented without the tireless efforts of ASHAs, health providers, and the team at
477 Innovators In Health.

478 This research was conducted through the Structured Operational Research and Training Initiative
479 (SORT IT), a global partnership led by the Special Programme for Research and Training in Tropical
480 Diseases at the World Health Organization (WHO/TDR). The model is based on a course
481 developed jointly by the International Union Against Tuberculosis and Lung Disease (The Union)
482 and Medécins sans Frontières (MSF/Doctors Without Borders). The specific SORT IT programme
483 which resulted in this publication was jointly developed and implemented by: The Union South-
484 East Asia Office, New Delhi, India; the Centre for Operational Research, The Union, Paris, France;
485 Department of Preventive and Social Medicine, Jawaharlal Institute of Postgraduate Medical
486 Education and Research, Puducherry, India; Department of Community Medicine and School of
487 Public Health, Postgraduate Institute of Medical Education and Research, Chandigarh, India;
488 Department of Community Medicine, All India Institute of Medical Sciences, Nagpur, India; Dr.
489 Rajendra Prasad Centre for Ophthalmic Sciences, All India Institute of Medical Sciences, New
490 Delhi, India; Department of Community Medicine, Pondicherry Institute of Medical Science,
491 Puducherry, India; Department of Community Medicine, Kalpana Chawla Medical College, Karnal,
492 India; National Centre of Excellence and Advance Research on Anemia Control, All India Institute
493 of Medical Sciences, New Delhi, India; Department of Community Medicine, Sri Manakula
494 Vinayagar Medical College and Hospital, Puducherry, India; Department of Community Medicine,
495 Velammal Medical College Hospital and Research Institute, Madurai, India; Department of
496 Community Medicine, Yenepoya Medical College, Mangalore, India; Karuna Trust, Bangalore,
497 India and National Institute for Research in Tuberculosis, Chennai, India.

For peer review only - http://bmjopen.bmj.com/site/about/guidelines.xhtml

COMPETING INTERESTS

None declared

FUNDING

This project was supported by the Stop TB Partnership's TB REACH initiative and was funded by the Government of Canada and the Bill & Melinda Gates Foundation. Manish Bhardwaj and Dyuti Sen are supported by a Grand Challenges Explorations grant number OPP1190735 and OPP1190905 respectively from the Bill & Melinda Gates Foundation. The training program, within which this paper was developed, was funded by the Department for International Development (DFID), UK. No funders had any role in study design, data collection and analysis, decision to publish, or preparation of the manuscript.

DATA AVAILABILITY

The deidentified data used in the study is available from the corresponding author on reasonable request. It contains information on patient demographics and characteristics, screening criteria, diagnostic test used, and final diagnosis. There is no additional data available beyond that.

ETHICS APPROVAL

Ethical approval was obtained from Institutional Ethics Committee, Emmanuel Hospital Association, New Delhi (Order 201) and the Ethics Advisory Group, International Union Against Tuberculosis and Lung Disease, Paris (EAG number 98/18). The committee waived the informed consent requirement for quantitative study. Informed consent was obtained from participants before the in-depth interviews and FGDs.

AUTHOR CONTRIBUTION

Conceptualization of intervention: TG, DS, MaB

Study design: TG, VG, DS, MV, MiB, RM, MaB

Data collection: TG, DS, RM

Data analysis and interpretation: TG, VG, DS, MV, RM, MaB

For peer review only

For peer review only - http://bmjopen.bmj.com/site/about/guidelines.xhtml

Writing — original draft: TG

Writing — review and edit: TG, VG, DS, MV, MiB, RM, MaB

REFERENCES

1 World Health Organization. Gear up to end TB: introducing the end TB strategy. World Health Organization 2015. https://apps.who.int/iris/handle/10665/156394?show=full (accessed 18 Nov 2019).

2 Reid MJA, Arinaminpathy N, Bloom A, *et al.* Building a tuberculosis-free world: The Lancet Commission on tuberculosis. *The Lancet* 2019;**393**:1331–84. doi:10/gfxc87

3 World Health Organization. *Global tuberculosis report 2019*. World Health Organization 2019. https://apps.who.int/iris/handle/10665/329368 (accessed 18 Oct 2019).

4 Lönnroth K, Corbett E, Golub J, *et al.* Systematic screening for active tuberculosis: rationale, definitions and key considerations [State of the art series. Active case finding/screening. Number 1 in the series]. *The International Journal of Tuberculosis and Lung Disease* 2013;**17**:289–98. doi:10/gd52dz

5 Storla DG, Yimer S, Bjune GA. A systematic review of delay in the diagnosis and treatment of tuberculosis. *BMC Public Health* 2008;**8**:15. doi:10/c6sz7r

6 Subbaraman R, Nathavitharana RR, Satyanarayana S, *et al.* The Tuberculosis Cascade of Care in India's Public Sector: A Systematic Review and Meta-analysis. *PLOS Medicine* 2016;**13**:e1002149. doi:10/f9b29f

7 Wells WA. Onions and prevalence surveys: how to analyze and quantify tuberculosis case-finding gaps. *The International Journal of Tuberculosis and Lung Disease* 2017;**21**:1101–13. doi:10/gb4g9c

8 Ho J, Fox GJ, Marais BJ. Passive case finding for tuberculosis is not enough. *International Journal of Mycobacteriology* 2016;**5**:374–8. doi:10/gcpfsf

9 Creswell J, Sahu S, Blok L, *et al.* A Multi-Site Evaluation of Innovative Approaches to Increase Tuberculosis Case Notification: Summary Results. *PLOS ONE* 2014;**9**:e94465. doi:10/gcz84n

10 Mhimbira FA, Cuevas, LE, Dacombe, R, Mkopi, A, Sinclair D. Interventions to increase tuberculosis case detection at primary healthcare or community-level services. *Cochrane Database of Systematic Reviews* Published Online First: 2017. doi:10/gcmjrv

11 Adejumo AO, Azuogu B, Okorie O, *et al.* Community referral for presumptive TB in Nigeria: a comparison of four models of active case finding. *BMC Public Health* 2016;**16**:177. doi:10/f8bjrx

For peer review only - http://bmjopen.bmj.com/site/about/guidelines.xhtml

12 Myint O, Saw S, Isaakidis P, *et al.* Active case-finding for tuberculosis by mobile teams in Myanmar: yield and treatment outcomes. *Infectious Diseases of Poverty* 2017;**6**:77. doi:10/gf38nz

13 Prasad BM, Satyanarayana S, Chadha SS, *et al.* Experience of active tuberculosis case finding in nearly 5 million households in India. *Public Health Action* 2016;**6**:15–8. doi:10/gd4v8q

14 Creswell JW, Clark VLP. *Designing and Conducting Mixed Methods Research*. SAGE 2011.

15 Registrar General of India. Census of India 2011. Office of the Registrar General Census Commissioner, India 2011.

16 Annual Health Survey 2011-12, Bihar Factsheet. New Delhi: : Office of the Registrar General & Census Commissioner http://www.censusindia.gov.in/vital_statistics/AHSBulletins/AHS_Factsheets_2011_12/Bihar_Factsheet_2011-12.pdf (accessed 3 Feb 2019).

17 Central TB Division. India TB Report 2018. New Delhi, India: : Directorate General of Health Services, Ministry of Health and Family Welfare, Government of India 2018. https://tbcindia.gov.in/showfile.php?lid=3314 (accessed 18 Nov 2019).

18 Uplekar M, Pathania V, Raviglione M. Private practitioners and public health: weak links in tuberculosis control. *The Lancet* 2001;**358**:912–6. doi:10/c8jf9s

19 National Informatics Centre, Patna. Health Department Geographical Information System, Bihar. http://gis.bih.nic.in/Depts/Health.aspx (accessed 14 Sep 2018).

20 Update on ASHA Programme. New Delhi: : National Health Mission, Ministy of Health and Family Welfare 2018. http://nhsrcindia.org/sites/default/files/asha%20reduced.pdf (accessed 29 Jul 2019).

21 Revised Technical & Operational Guidelines for Tuberculosis Control in India. New Delhi: : Central TB Division 2016. https://www.tbcindia.gov.in/WriteReadData/Revised%20Technical%20and%20Operational%20Guidelines/files/assets/basic-html/page-1.html# (accessed 4 Feb 2019).

22 Integrated Child Development Services Scheme. Ministry of Women and Child Development, Government of India. https://icds-wcd.nic.in/icds.aspx (accessed 17 Aug 2019).

23 Attride-Stirling J. Thematic networks: an analytic tool for qualitative research. *Qualitative research* 2001;**1**:385–405. doi:10/cg4grf

24 Lincoln Y, Guba E. Naturalistic Inquiry: Designing a naturalistic inquiry. *Beverly Hills: Sage Publications* 1985.

For peer review only - http://bmjopen.bmj.com/site/about/guidelines.xhtml

25 Mundy C. J. F, Harries A. D, Banerjee A, *et al.* Quality assessment of sputum transportation, smear preparation and AFB microscopy in a rural district in Malawi. *The International Journal of Tuberculosis and Lung Disease* 2002;**6**:47–54.

26 Drain PK, Garrett NJ. The arrival of a true point-of-care molecular assay—ready for global implementation? *The Lancet Global Health* 2015;**3**:e663–4. doi:10/ggdfph

27 Lee DJ, Kumarasamy N, Resch SC, *et al.* Rapid, point-of-care diagnosis of tuberculosis with novel Truenat assay: Cost-effectiveness analysis for India's public sector. *PLOS ONE* 2019;**14**:e0218890. doi:10/ggdfpj

28 Padingani M, Kumar A, Tripathy JP, *et al.* Does pre-diagnostic loss to follow-up among presumptive TB patients differ by type of health facility? An operational research from Hwange, Zimbabwe in 2017. *Pan African Medical Journal* 2018;**31**. doi:10.11604/pamj.2018.31.196.15848

29 Sarin R, Vohra V, Singla N, *et al.* Identifying costs contributing to catastrophic expenditure among TB patients registered under RNTCP in Delhi metro city in India. *Indian Journal of Tuberculosis* 2019;**66**:150–7. doi:10/ggdfpz

30 Tanimura T, Jaramillo E, Weil D, *et al.* Financial burden for tuberculosis patients in low- and middle-income countries: a systematic review. *European Respiratory Journal* 2014;**43**:1763–75. doi:10/f56btb

31 Sangar S, Dutt V, Thakur R. Economic burden, impoverishment and coping mechanisms associated with out-of-pocket health expenditure: analysis of rural-urban differentials in India. *J Public Health (Berl)* 2018;**26**:485–94. doi:10/ggdfpx

32 Tripathy JP, Srinath S, Naidoo P, *et al.* Is physical access an impediment to tuberculosis diagnosis and treatment? A study from a rural district in North India. *Public Health Action* 2013;**3**:235–9. doi:10/ggdfp8

33 Kasthuri A. Challenges to Healthcare in India - The Five A's. *Indian J Community Med* 2018;**43**:141–3.

34 Bhatnagar H. User-experience and patient satisfaction with quality of tuberculosis care in India: A mixed-methods literature review. *Journal of Clinical Tuberculosis and Other Mycobacterial Diseases* 2019;**17**:100127. doi:10/ggdfn3

35 Anand G, Chhajed D, Shah S, *et al.* Do qualifications matter? A qualitative study of how villagers decide their health care providers in a developing economy. *PLOS ONE* 2019;**14**:e0220316. doi:10/ggdfqr

36 Kumar S, Kaushik A, Kansal S. Factors Influencing the Work Performance of ASHA Under NRHM: A Cross Sectional Study from Eastern Uttar Pradesh. *Indian Journal of Community Health* 2012;**24**:325–31.

For peer review only

37 Roy K, Chaudhuri A. Influence of socioeconomic status, wealth and financial empowerment on gender differences in health and healthcare utilization in later life: evidence from India. *Social Science & Medicine* 2008;**66**:1951–62. doi:10/dhww4j

38 Heijnders M, Meij SVD. The fight against stigma: An overview of stigma-reduction strategies and interventions. *Psychology, Health & Medicine* 2006;**11**:353–63. doi:10/ddr4bb

39 Brody C, Hoop T de, Vojtkova M, *et al.* Economic Self-Help group Programs for Improving Women's Empowerment: A Systematic Review. *Campbell Systematic Reviews* 2015;**11**:1–182. doi:10/ggdfqv

40 Brody C, Hoop T de, Vojtkova M, *et al.* Can self-help group programs improve women's empowerment? A systematic review. *Journal of Development Effectiveness* 2017;**9**:15–40. doi:10/gf44gk

41 Balarajan Y, Selvaraj S, Subramanian S. Health care and equity in India. *The Lancet* 2011;**377**:505–15. doi:10/crkb78

42 Agins BD, Ikeda DJ, Reid MJA, *et al.* Improving the cascade of global tuberculosis care: moving from the "what" to the "how" of quality improvement. *The Lancet Infectious Diseases* doi:10/ggdfq6

43 Wells WA, Uplekar M, Pai M. Achieving Systemic and Scalable Private Sector Engagement in Tuberculosis Care and Prevention in Asia. *PLOS Medicine* 2015;**12**:e1001842. doi:10/ggdfq8

44 Gautham M, Shyamprasad KM, Singh R, *et al.* Informal rural healthcare providers in North and South India. *Health Policy Plan* 2014;**29**:i20–9. doi:10/f6cp72

45 Sudhinaraset M, Ingram M, Lofthouse HK, *et al.* What Is the Role of Informal Healthcare Providers in Developing Countries? A Systematic Review. *PLOS ONE* 2013;**8**:e54978. doi:10/ggdfq3

46 Ergler CR, Sakdapolrak P, Bohle H-G, *et al.* Entitlements to health care: Why is there a preference for private facilities among poorer residents of Chennai, India? *Social Science & Medicine* 2011;**72**:327–37. doi:10/bqs6px

47 Sinha P, Shenoi SV, Friedland GH. Opportunities for community health workers to contribute to global efforts to end tuberculosis. *Global Public Health* 2019;**0**:1–11. doi:10/ggdfqx

For peer review only - http://bmjopen.bmj.com/site/about/guidelines.xhtml

651 **LEGEND FOR FIGURES**

652 **Figure 1: Operational definitions used in the program**

653 CHW: Community Health Worker

654 ASHA: Accredited Social Health Activist

655 AWW: Anganwadi Worker

656 CXR: Chest X-ray

657 USG: Ultrasonography

658 **Figure 2: TB care cascade between January 2018 and December 2018 from community referral**
659 **to diagnostic evaluation in a community-based ACF program in Samastipur, India**

660 All percentages are calculated as a proportion of the number of participants entering the previous
661 step of the cascade.

662 * Includes 140 Presumptive TB cases undergoing diagnostic evaluation beyond 30 days from
663 screening.

664 **Figure 3: The enablers and barriers in diagnostic evaluation from patients', ashas', and field**
665 **coordinators' perspective in an ACF TB program in Samastipur, India from January 2018 to**
666 **December 2018**

667 [a]: responses of patient

668 [b]: responses of ASHA

669 [c]: responses of FC

670 ASHA: Accredited Social Health Activist

671 PHS: Public Health System

672 PHC: Primary Health Centre

For peer review only - http://bmjopen.bmj.com/site/about/guidelines.xhtml

For peer review only

Term	Definition
Community referral	A person at risk of TB referred for TB screening to the program by a CHW (ASHA or AWW) or an informal provider or community during their routine work
Presumptive TB patient	A person identified with one or more of the following symptoms of TB in the screening process at their home: cough ≥ 2 weeks; sputum in cough, hemoptysis in last 6 months; chest pain in last 1 month; fever ≥ 2 weeks; night sweats ≥ 2 weeks; severe weight loss in last 3 months; swelling in a lymph node
Diagnostic evaluation	A presumptive TB patient tested using one or more of a sputum microscopy or CXR or GeneXpert or extrapulmonary TB testing (USG or FNAC) within 30 days of screening
Pre-diagnostic loss to follow-up	A presumptive TB patient not undergoing a diagnostic evaluation

For peer review only - http://bmjopen.bmj.com/site/about/guidelines.xhtml

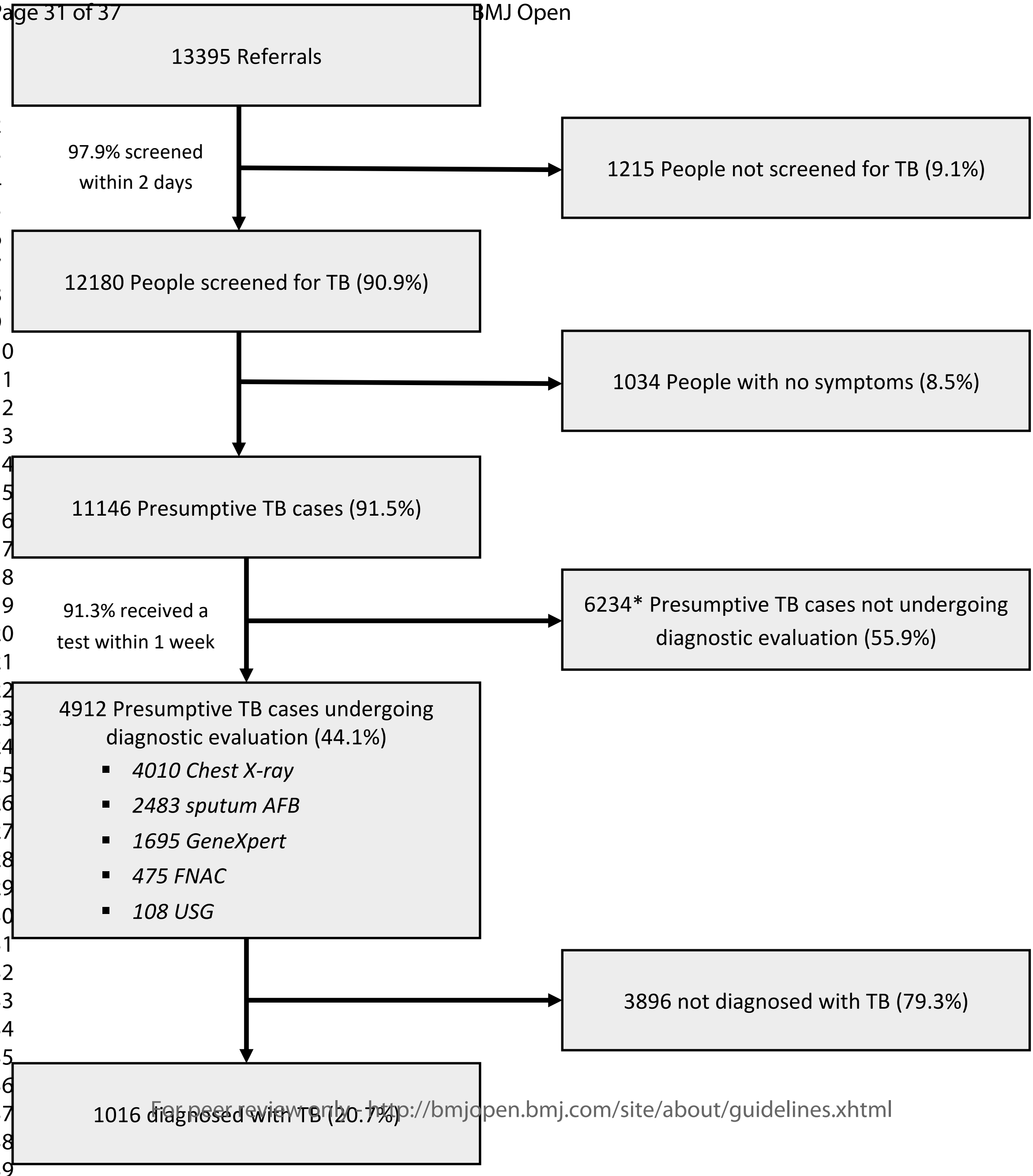

For peer review only - http://bmjopen.bmj.com/site/about/guidelines.xhtml

Enablers

Category 1: Logistics-related
- Transport allowance [a,b,c]

Category 2: Health system-related
- Free services in the PHS [a,b]
- Knowledge of the PHS procedures [a]
- Incentive to ASHA for TB work is vital [b,c]
- Previous positive patient experience in PHS [b,c]

Category 3: Health provider-related
- ASHA's assistance in the diagnostic process [a]
- ASHA's accompaniment to the health facility [a,c]
- ASHA's understanding of the PHS functioning [a]
- Satisfied people referred others to ASHA [b]

Barriers

Category 1: Logistics-related
- Unavailability of public transport from patient's village [a,c]
- No knowledge of the diagnostic center location [a]
- No transport support for the disabled patient [b]

Category 2: Health system-related
- Care at PHC was slow and time-taking [a,b,c]
- Not all diagnostics were available in the PHC [b]
- High cost of services not available in the PHC [b]
- Difficult to understand PHC system [a,b]
- PHC remained closed [a]
- Inability to reach PHC in working hours [a,b]
- Provider was unavailable at the PHC [a]
- Irregular and unpaid ASHA incentives [b]
- Faith in the private sector [b,c]
- Poor patient experience deterred others as well [b]

Category 3: Health provider-related
- Informal providers misguided the patient [b,c]
- Low morale of the health providers [b]
- Health worker strikes [a]
- Poor health provider behavior at PHC [b,c]
- ASHA didn't come to the patient's home [a]

Category 4: Family-related
- Poor awareness of TB care in PHS [a,c]
- Family concerned only when symptoms were severe [b]
- Insufficient family support [a]
- No caretaker in the family to help in reaching hospital [a]
- Family permission was necessary [b]
- Indebted, and no money for healthcare [a]
- Restricted mobility of women [b]

Category 5: Patient-related
- Denial of suffering from TB [a]
- Fear of TB [b]
- Discrimination against diseased patient [b,c]

For peer review only - http://bmjopen.bmj.com/site/about/guidelines.xhtml

SUPPLEMENTARY TABLE 1

Sensitivity analysis on including patient with negative diagnostic delay as zero day delay, after excluding patients with diagnostic evaluation before screening visit, and classifying patients with over 30 days delay in diagnostic evaluation as non-PDLFU

	Including patients with negative diagnostic delay as 0 days diagnostic delay[$]						Excluding patients with negative diagnostic delay[$]						Including patients with diagnostic delay of over 30 days as non-PDLFU					
Characteristics	Univariate analysis			Multivariate analysis			Univariate analysis			Multivariate analysis			Univariate analysis			Multivariate analysis		
	RR	95%CI	p-Value	aRR^	95%CI	p-Value	RR	95%CI	p-Value	aRR^	95%CI	p-Value	RR	95%CI	p-Value	aRR^	95%CI	p-Value
**Block**																		
Ujiarpur	1.0	(1.0-1.1)	0.022	-			1.1	(1.0-1.1)	0.004	-			1.0	(1.0-1.1)	0.04	-		
Bibhutipur	1.0	(1.0-1.1)	0.033	-			1.0	(1.0-1.1)	0.033	-			1.0	(1.0-1.1)	0.08	-		
Sarairanjan		Ref		-				Ref		-				Ref		-		
**Age**																		
<15	1.4	(1.3-1.4)	<0.001	1.2	(1.2-1.3)	<0.001	1.3	(1.3-1.4)	<0.001	1.3	(1.2-1.3)	<0.001	1.3	(1.2-1.4)	<0.001	1.2	(1.1-1.3)	<0.001
15-44	1.0	(1.0-1.1)	0.139	1.0	(1.0-1.1)	0.366	1.1	(1.0-1.1)	0.06	1.0	(1.0-1.1)	0.180	1.0	(1.0-1.1)	0.216	1.0	(1.0-1.1)	0.539
45-64	1.0	(1.0-1.1)	0.212	1.0	(1.0-1.1)	0.164	1.1	(1.0-1.1)	0.143	1.1	(1.0-1.1)	0.099	1.0	(1.0-1.1)	0.295	1.0	(1.0-1.1)	0.242
≥65		Ref			Ref			Ref			Ref			Ref			Ref	
**Gender**																		
Female		Ref			Ref			Ref			Ref			Ref			Ref	
Male	1.1	(1.0-1.1)	<0.001	1.1	(1.0-1.1)	0.001	1.1	(1.0-1.1)	<0.001	1.1	(1.0-1.1)	0.003	1.1	(1.0-1.1)	<0.001	1.1	(1.0-1.1)	<0.001
**Source of referral**																		
RMP[3]		Ref			Ref			Ref			Ref			Ref			Ref	
ASHA[1]	1.3	(1.2-1.4)	<0.001	1.2	(1.1-1.3)	<0.001	1.3	(1.2-1.4)	<0.001	1.2	(1.1-1.3)	<0.001	1.3	(1.2-1.4)	<0.001	1.2	(1.1-1.4)	<0.001
AWW[2]	1.3	(1.1-1.6)	0.003	1.2	(1.0-1.5)	0.025	1.3	(1.1-1.6)	0.005	1.2	(1.1-1.5)	0.030	1.4	(1.1-1.7)	<0.001	1.3	(1.1-1.6)	0.012
Community	1.4	(1.3-1.5)	<0.001	1.3	(1.2-1.4)	<0.001	1.4	(1.3-1.5)	<0.001	1.3	(1.2-1.4)	<0.001	1.4	(1.3-1.6)	<0.001	1.2	(1.2-1.5)	<0.001
**Signs and symptoms**																		
Haemoptysis in last 6 months	0.6	(0.6-0.7)	<0.001	0.7	(0.6-0.7)	<0.001	0.6	(0.6-0.7)	<0.001	0.8	(0.7-0.8)	<0.001	0.6	(0.6-0.7)	<0.001	0.7	(0.6-0.7)	<0.001
Cough ≥ 2 weeks	0.7	(0.6-0.7)	<0.001	-			0.7	(0.7-0.7)	<0.001	-			0.7	(0.6-0.7)	<0.001	-		
Sputum	0.6	(0.6-0.7)	<0.001	-			**0.7**	**(0.6-0.7)**	<0.001	-			0.6	(0.6-0.7)	<0.001	-		
Chest pain in last 1 month	0.6	(0.6-0.7)	<0.001	-			**0.7**	**(0.6-0.7)**	<0.001	-			0.6	(0.6-0.7)	<0.001	-		
Fever ≥ 2 weeks	0.8	(0.8-0.8)	<0.001	-			0.8	(0.8-0.8)	<0.001	-			0.8	(0.7-0.8)	<0.001	-		
Night sweats ≥ 2 weeks	0.8	(0.7-0.8)	<0.001	-			0.8	(0.7-0.8)	<0.001	-			0.8	(0.7-0.8)	<0.001	-		
Severe weight loss in last 3 months	0.7	(0.7-0.8)	<0.001	-			0.7	(0.7-0.8)	<0.001	-			0.7	(0.7-0.8)	<0.001	-		
Swelling in a lymph node	1.4	(1.4-1.5)	<0.001	-			1.4	(1.3-1.4)	<0.001	-			1.4	(1.3-1.4)	<0.001	-		

For peer review only

**Other factors**																		
Previous history of anti-TB treatment	0.7	(0.6-0.7)	<0.001	0.7	(0.7-0.8)	<0.001	0.7	(0.7-0.7)	<0.001	0.8	(0.7-0.8)	<0.001	0.7	(0.6-0.7)	<0.001	0.7	(0.7-0.8)	<0.001
Alcohol user	1.0	(0.9-1.2)	0.862	-			1.0	(0.9-1.1)	0.980	-			1.0	(0.9-1.1)	0.882	-		
Tobacco user	0.8	(0.8-0.9)	<0.001	-			0.9	(0.8-0.9)	<0.001	-			0.8	(0.8-0.9)	<0.001	-		

Bold variables indicate difference in RR

[$] Diagnostic delay is calculated as difference between date of first diagnostic evaluation and date of screening (Figure 1)

^aRR: Adjusted Risk Ratio

[1]ASHA: Accredited Social Health Activist

[2]AWW: Anganwadi Worker

[3]RMP: Registered Medical Practitioner, an informal provider

p-value ≤ 0.05 considered significant

* While building the model for multivariate Poisson's regression analysis, all the signs and symptoms except hemoptysis were dropped because of high collinearity assessed on the basis of high VIF (Variance Inflation Factor). In addition, block and other factors like alcohol and tobacco use depicted no significant improvement in the model on the basis of likelihood ratio testing, hence, were also dropped.

For peer review only

COREQ (COnsolidated criteria for REporting Qualitative research) Checklist

A checklist of items that should be included in reports of qualitative research. You must report the page number in your manuscript where you consider each of the items listed in this checklist. If you have not included this information, either revise your manuscript accordingly before submitting or note N/A.

Topic	Item No.	Guide Questions/Description	Reported on Page No.
**Domain 1: Research team and reflexivity**			
Personal characteristics			
Interviewer/facilitator	1	Which author/s conducted the interview or focus group?	
Credentials	2	What were the researcher's credentials? E.g. PhD, MD	
Occupation	3	What was their occupation at the time of the study?	
Gender	4	Was the researcher male or female?	
Experience and training	5	What experience or training did the researcher have?	
Relationship with participants			
Relationship established	6	Was a relationship established prior to study commencement?	
Participant knowledge of the interviewer	7	What did the participants know about the researcher? e.g. personal goals, reasons for doing the research	
Interviewer characteristics	8	What characteristics were reported about the inter viewer/facilitator? e.g. Bias, assumptions, reasons and interests in the research topic	
**Domain 2: Study design**			
Theoretical framework			
Methodological orientation and Theory	9	What methodological orientation was stated to underpin the study? e.g. grounded theory, discourse analysis, ethnography, phenomenology, content analysis	
Participant selection			
Sampling	10	How were participants selected? e.g. purposive, convenience, consecutive, snowball	
Method of approach	11	How were participants approached? e.g. face-to-face, telephone, mail, email	
Sample size	12	How many participants were in the study?	
Non-participation	13	How many people refused to participate or dropped out? Reasons?	
Setting			
Setting of data collection	14	Where was the data collected? e.g. home, clinic, workplace	
Presence of non-participants	15	Was anyone else present besides the participants and researchers?	
Description of sample	16	What are the important characteristics of the sample? e.g. demographic data, date	
Data collection			
Interview guide	17	Were questions, prompts, guides provided by the authors? Was it pilot tested?	
Repeat interviews	18	Were repeat inter views carried out? If yes, how many?	
Audio/visual recording	19	Did the research use audio or visual recording to collect the data?	
Field notes	20	Were field notes made during and/or after the inter view or focus group?	
Duration	21	What was the duration of the inter views or focus group?	
Data saturation	22	Was data saturation discussed?	
Transcripts returned	23	Were transcripts returned to participants for comment and/or	

For peer review only

Topic	Item No.	Guide Questions/Description	Reported on Page No.
		correction?	
**Domain 3: analysis and findings**			
Data analysis			
Number of data coders	24	How many data coders coded the data?	
Description of the coding tree	25	Did authors provide a description of the coding tree?	
Derivation of themes	26	Were themes identified in advance or derived from the data?	
Software	27	What software, if applicable, was used to manage the data?	
Participant checking	28	Did participants provide feedback on the findings?	
Reporting			
Quotations presented	29	Were participant quotations presented to illustrate the themes/findings? Was each quotation identified? e.g. participant number	
Data and findings consistent	30	Was there consistency between the data presented and the findings?	
Clarity of major themes	31	Were major themes clearly presented in the findings?	
Clarity of minor themes	32	Is there a description of diverse cases or discussion of minor themes?	

Developed from: Tong A, Sainsbury P, Craig J. Consolidated criteria for reporting qualitative research (COREQ): a 32-item checklist for interviews and focus groups. *International Journal for Quality in Health Care*. 2007. Volume 19, Number 6: pp. 349 – 357

Once you have completed this checklist, please save a copy and upload it as part of your submission. DO NOT include this checklist as part of the main manuscript document. It must be uploaded as a separate file.

For peer review only

STROBE 2007 (v4) Statement—Checklist of items that should be included in reports of *cohort studies*

Section/Topic	Item #	Recommendation	Reported on page #
**Title and abstract**	1	(*a*) Indicate the study's design with a commonly used term in the title or the abstract	1
		(*b*) Provide in the abstract an informative and balanced summary of what was done and what was found	3, 4
**Introduction**			
Background/rationale	2	Explain the scientific background and rationale for the investigation being reported	5, 6
Objectives	3	State specific objectives, including any prespecified hypotheses	5, 6
**Methods**			
Study design	4	Present key elements of study design early in the paper	6
Setting	5	Describe the setting, locations, and relevant dates, including periods of recruitment, exposure, follow-up, and data collection	6 – 8
Participants	6	(*a*) Give the eligibility criteria, and the sources and methods of selection of participants. Describe methods of follow-up	8
		(*b*) For matched studies, give matching criteria and number of exposed and unexposed	Not applicable
Variables	7	Clearly define all outcomes, exposures, predictors, potential confounders, and effect modifiers. Give diagnostic criteria, if applicable	8, 9
Data sources/ measurement	8*	For each variable of interest, give sources of data and details of methods of assessment (measurement). Describe comparability of assessment methods if there is more than one group	8, 9
Bias	9	Describe any efforts to address potential sources of bias	9, 10
Study size	10	Explain how the study size was arrived at	8
Quantitative variables	11	Explain how quantitative variables were handled in the analyses. If applicable, describe which groupings were chosen and why	9, 10
Statistical methods	12	(*a*) Describe all statistical methods, including those used to control for confounding	9, 10
		(*b*) Describe any methods used to examine subgroups and interactions	9, 10
		(*c*) Explain how missing data were addressed	9
		(*d*) If applicable, explain how loss to follow-up was addressed	5 – 7, 9
		(*e*) Describe any sensitivity analyses	Not Applicable
**Results**			

For peer review only

Participants	13*	(a) Report numbers of individuals at each stage of study—eg numbers potentially eligible, examined for eligibility, confirmed eligible, included in the study, completing follow-up, and analysed	10 – 12
		(b) Give reasons for non-participation at each stage	10 – 17
		(c) Consider use of a flow diagram	Figure 2
Descriptive data	14*	(a) Give characteristics of study participants (eg demographic, clinical, social) and information on exposures and potential confounders	10 – 12
		(b) Indicate number of participants with missing data for each variable of interest	9
		(c) Summarise follow-up time (eg, average and total amount)	Figure 2
Outcome data	15*	Report numbers of outcome events or summary measures over time	Figure 2
Main results	16	(*a*) Give unadjusted estimates and, if applicable, confounder-adjusted estimates and their precision (eg, 95% confidence interval). Make clear which confounders were adjusted for and why they were included	Table 2
		(*b*) Report category boundaries when continuous variables were categorized	Table 2
		(*c*) If relevant, consider translating estimates of relative risk into absolute risk for a meaningful time period	Not Applicable
Other analyses	17	Report other analyses done—eg analyses of subgroups and interactions, and sensitivity analyses	Not Applicable
**Discussion**			
Key results	18	Summarise key results with reference to study objectives	17 – 18
**Limitations**			
Interpretation	20	Give a cautious overall interpretation of results considering objectives, limitations, multiplicity of analyses, results from similar studies, and other relevant evidence	19
Generalisability	21	Discuss the generalisability (external validity) of the study results	19
**Other information**			
Funding	22	Give the source of funding and the role of the funders for the present study and, if applicable, for the original study on which the present article is based	21

*Give information separately for cases and controls in case-control studies and, if applicable, for exposed and unexposed groups in cohort and cross-sectional studies.

Note: An Explanation and Elaboration article discusses each checklist item and gives methodological background and published examples of transparent reporting. The STROBE checklist is best used in conjunction with this article (freely available on the Web sites of PLoS Medicine at http://www.plosmedicine.org/, Annals of Internal Medicine at http://www.annals.org/, and Epidemiology at http://www.epidem.com/). Information on the STROBE Initiative is available at www.strobe-statement.org.

For peer review only
